# Enantioselective Addition of Dialkyl Malonates to β-Arylethenesulfonyl Fluorides under High-Pressure Conditions

**DOI:** 10.1021/acs.orglett.3c02302

**Published:** 2023-09-01

**Authors:** Michał Kopyt, Michał Tryniszewski, Michał Barbasiewicz, Piotr Kwiatkowski

**Affiliations:** † Faculty of Chemistry, University of Warsaw, Pasteura 1, 02-093 Warsaw, Poland; ‡ Biological and Chemical Research Centre, University of Warsaw, Żwirki i Wigury 101, 02-089 Warsaw, Poland

## Abstract

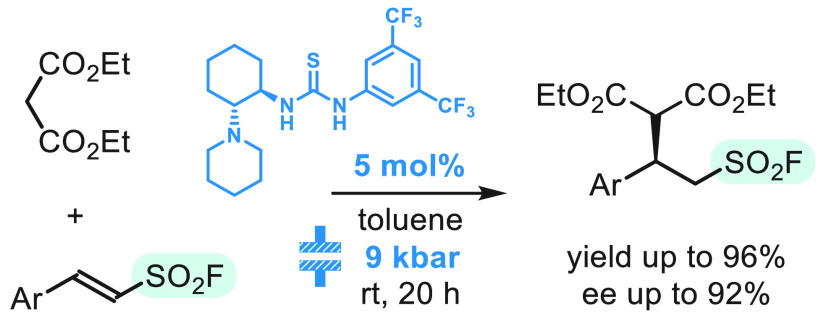

Application of high-pressure conditions enables enantioselective Michael-type addition of dialkyl malonates to β-arylethenesulfonyl fluorides. The reaction is efficiently catalyzed with 5 mol % of tertiary amino-thiourea at 9 kbar. Chiral alkanesulfonyl fluorides are formed in yields of up to 96% and enantioselectivities of up to 92%. Functionalization of the adducts via sulfur fluoride exchange (SuFEx) reaction and desulfonylative cyclization is demonstrated.

Sulfonyl fluorides^1^ are valuable class of compounds applied as protein inhibitors,^2^ polymer building blocks, and substrates for organic transformations.^3^ Their unique combination of stability and reactivity triggered with hydrogen bonds or silicon species enables “click”-type transformations known as the sulfur fluoride exchange (SuFEx) reaction.^4^ As activation of the sulfonyl group toward substitution is a key aspect of studies, both “actor” and “spectator” reactions in which the fluoride is being replaced or remains intact are of particular interest. For β-arylethenesulfonyl fluorides (ArCH=CHSO_2_F), both types of the processes are known and give, for example, sulfonates with aryl silyl ethers, conjugate addition products with secondary amines, or cyclic sultams, via sequence of addition–substitution with hydrazines.^5^ In contrast to ethenesulfonyl fluoride (CH_2_=CHSO_2_F, ESF),^6^ known as “the most perfect Michael-acceptor ever found,” β-arylsubstituted analogues are ∼4.5 orders of magnitude less electrophilic,^7^ thus, their reactions with C-nucleophiles remain a challenging task. Key literature examples of such processes are presented in Scheme 1. Lupton et al. reported NHC-catalyzed addition of silyl ethers of dimedone, which gave δ-sultones.^8,9^ Qin et al. tested similar DBU-promoted reactions with pyrazolones and 1,3-diketones, whereas diethyl malonate was shown to react with 4-nitrosubstituted substrate by following a pathway of addition–elimination of the fluorosulfonyl group.^10^ The same author also reported reactions with malononitrile and cyanomalonate, which resulted in substituted cyclopropanes.^11^ Only recently has water-accelerated phosphazene-catalyzed addition of di*thio*malonates been demonstrated in a racemic variant.^12^ However, under these conditions, an adduct with dibenzyl malonate was formed in only trace amount (<3% of yield), despite presence of advantageous biphasic medium and superbase catalysis. Interestingly, the “on-water” acceleration^13^ postulated by Cheong and Bae et al.^12,14^ was attributed to confined water-embraced organic cages formed upon vigorous stirring, which display a pressure-like effect.^15^ In our report, we present addition of dialkyl malonates to a series of β-arylethenesulfonyl fluorides in an *enantioselective*(16) organocatalyzed variant under homogeneous *high-pressure* conditions. We conclude about action of the catalytic system on the basis of product configuration and demonstrate transformations of the chiral adducts via SuFEx and desulfonylative cyclization.

**Scheme 1:**
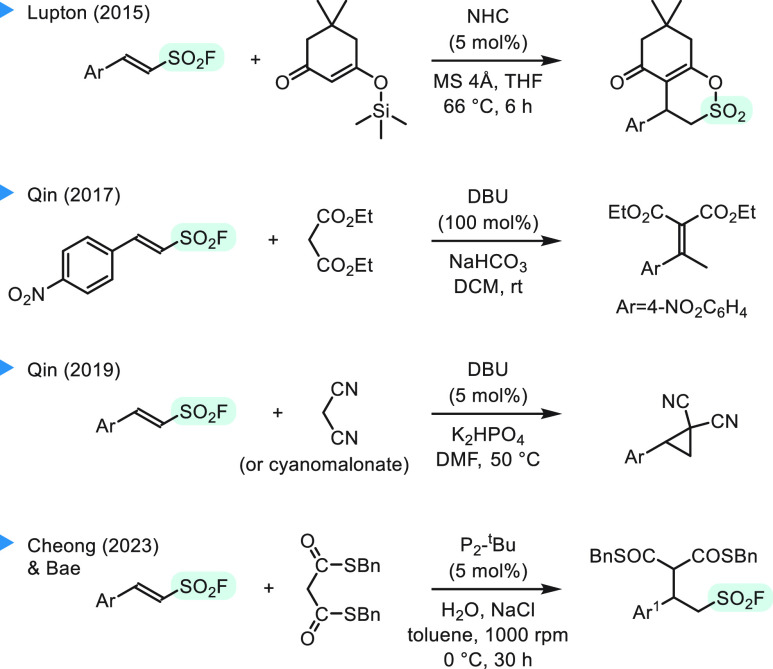
Literature Examples of Michael-type Additions of Enolates to β-Arylethenesulfonyl Fluorides

Recently, we reported synthesis of ArCH=CHSO_2_F via olefination^17^ of arylaldehydes with methanedisulfonyl fluoride (FSO_2_CH_2_SO_2_F, MDSF).^18^ With the set of substrates in hand, we tested a model reaction of β-phenylethenesulfonyl fluoride (**1a**) with diethyl malonate (Table 1), thereby giving adduct **2a** in the presence of chiral thiourea, urea, and squaramide organocatalysts **3a**–**l**^19^ (Figure 1).

**Table 1:**
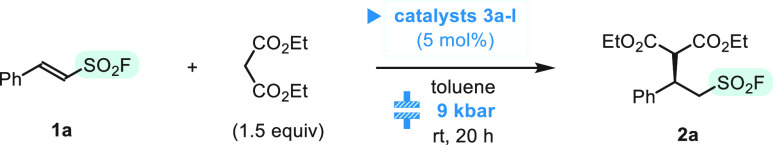
Organocatalyst Screening and Optimization of Model Reaction between β-Phenylethenesulfonyl Fluoride and Diethyl Malonate^a^

entry	catalyst	*c*_**1a**_ [mol/dm^3^]	variations of conditions	conversion (NMR)^b^	ee [%]^c^
1	**3a**	1.0		99%	78 (*R*)
2	**3b**	1.0		97%	73 (*R*)
3	**3c**	1.0		>99%	82 (*R*)
4	**3d**	1.0		>99%	84 (*R*)
5	**3e**	1.0		94%	75 (*R*)
6	**3f**	1.0		>99%	75 (*R*)
7	**3g**^d^	1.0		<1%	
8	**3h**	1.0		24%	79 (*R*)
9	**3i**	1.0		90%	20 (*R*)
10	**3j**	1.0		91%	82 (*R*)
11	**3k**	1.0		99%	83 (*S*)
12	**3l**	1.0		41%	11 (*R*)
13	**3d**	1.0	DCM	82%	78 (*R*)
14	**3d**	1.0	THF	95%	71 (*R*)
15	**3d**	1.0	6 kbar	96%	86 (*R*)
16	**3d**	1.0	6 kbar, 2 mol % **3d**	49%	85 (*R*)
17	**3d**	0.5	2 h	98%	89 (*R*)
18	**3d**	0.5	2 mol % **3d**	93%	89 (*R*)
19	**3d**	0.5		>99% (94%)^e^	89 (*R*)
20	**3d**	0.5	1 bar, 7 d	∼4%	

a Reaction conditions, unless stated otherwise: **1a** (0.2 mmol), diethyl malonate (0.3 mmol), catalyst **3** (0.01 mmol), toluene (∼0.15 mL), 9 kbar, rt, 20 h.

b NMR analysis of the crude reaction mixtures revealed presence of **1a** and **2a**, exclusively.

c Determined by HPLC analysis using Chiralpak IC column.

d Catalyst **3g** was poorly soluble in toluene.

e Isolated yield.

**Figure 1:**
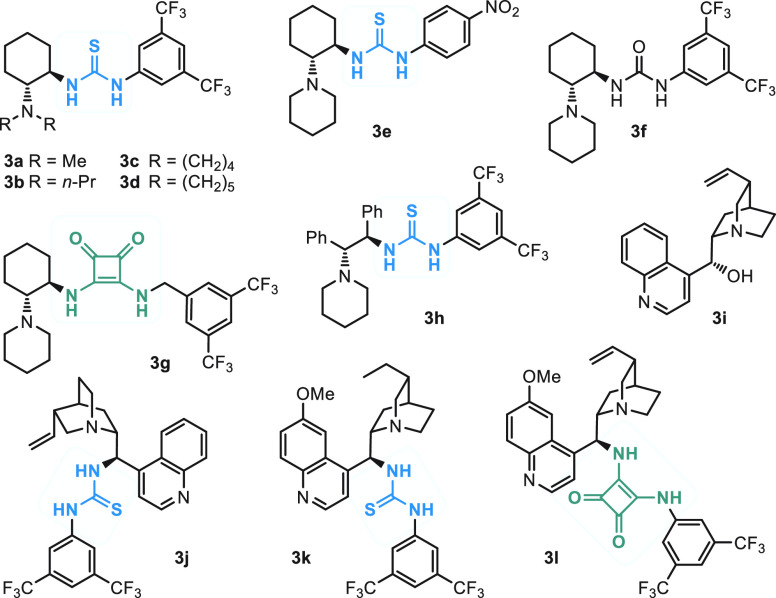
Organocatalysts **3a**–**l** tested in screening presented in Table 1 (thiourea and squaramide fragments are highlighted in blue and green, respectively).

First, screening of the catalysts in toluene solutions at 9 kbar led to identification of thioureas **3d** and **3k** as the most promising variants in terms of enantioselectivity and conversion (Table 1, entries 1–12).^20^ Changes of the solvent (entries 4, 13, and 14) also controlled the reaction course, with toluene combining the highest ee values and reasonable solubility of substrates (model reaction mixture at *c*_**1a**_ = 1.0 M formed a quasi-saturated solution). When the process was run at a lower pressure of 6 kbar (entry 15) and for a shortened time (2 h, entry 17), it remained highly efficient, and substrate conversion decreased only when catalyst loading was lowered to 2 mol % (entries 16 and 18). Finally, we observed that in a more diluted solution (*c*_**1a**_ = 0.5 M) enantioselectivity was improved, and in preparative experiment, adduct **2a** was isolated in 94% and 89% ee (entry 19).^20^ Importantly, the same reaction repeated under atmospheric pressure gave only traces of **2a** (∼4% after 7 days, entry 20), which clearly demonstrated effect of the high pressure on the reaction rate. It is noteworthy that despite hydrogen bond complexation with the organocatalyst, the fluorosulfonyl group remained perfectly stable toward substitution on the reaction course. The observation corroborates literature data on sulfonyl fluorides^1a,4^ which under the high-pressure conditions, yet unprecedented for SuFEx chemistry, fully support the term ‘sleeping beauties’.^3^

Next, we studied scope of the reaction for various β-arylethenesulfonyl fluorides, **1a**–**r** (Scheme 2). After 20 h, we observed practically complete conversion^21^ of all sulfonyl fluorides, except **1p** and **1r**, which were less soluble in toluene. Although the problem of solubility can be overcome by using less concentrated reaction mixtures, this approach is not recommended because of limited working volume of the high-pressure reactors and demand for use of higher catalyst and nucleophile loading.^20^ In turn, use of a solvent in which solubility is better, e.g., THF, resulted in a lower enantiomeric purity of the adduct (60% ee for **2r**). In the remaining substrate series, presence of substituents at the aromatic ring was well-tolerated, and enantioselectivity ranged from 82 to 92%. Only for adduct **2o**, which bears two fluorine atoms at the ortho positions, was the ee lowered (73%). Moreover, a very good result was obtained with dimethyl malonate, where product **4** was isolated in 95% yield and 92% ee.

**Scheme 2:**
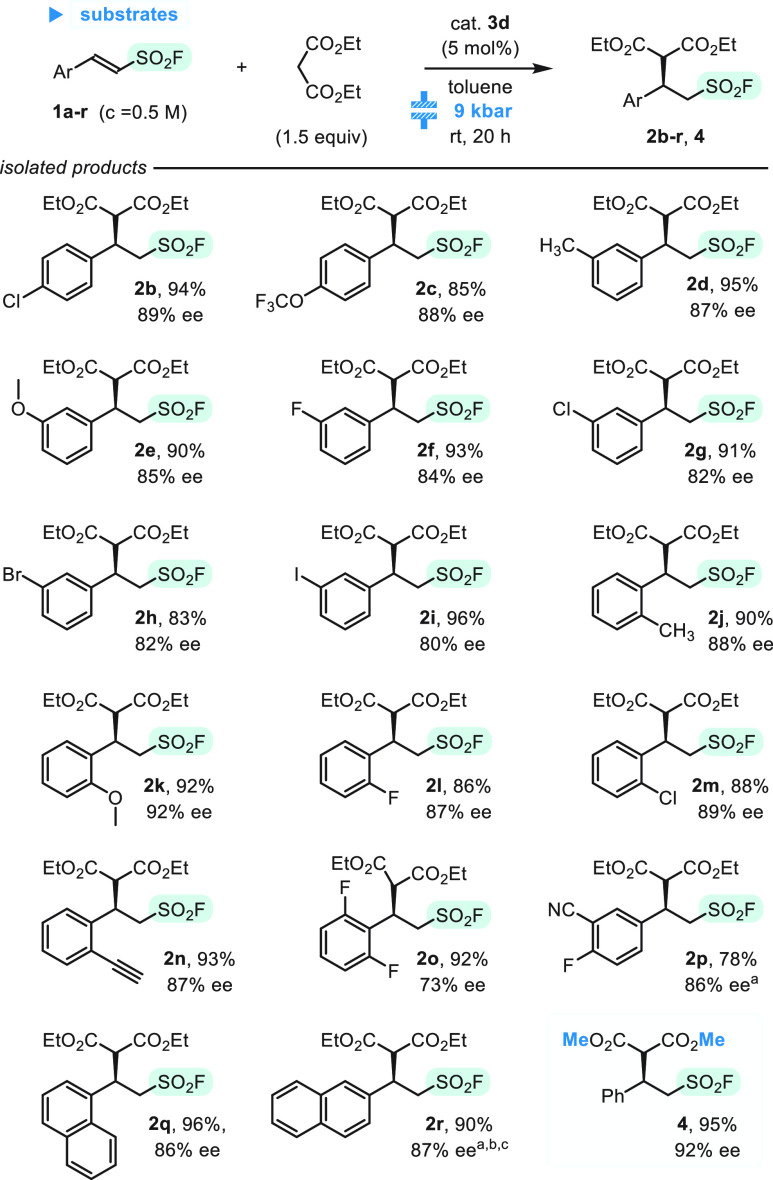
High-Pressure Organocatalytic Addition of Malonates to β-Arylethenesulfonyl Fluorides (**1a**–**r**) Substrates **1p** and **1r** were less soluble in toluene. The reaction was carried out at *c*_**1r**_ = 0.15 M with 10 mol % of **3d** and 3 equiv of diethyl malonate. The reaction repeated under standard conditions in THF afforded **2r**, with an isolated yield of 83% and 60% ee.^20^

The structure and absolute configuration of representative adduct **2g** was established using X-ray crystallography^20^ (Scheme 3, left). Accordingly, use of catalyst (1*R*,2*R*)-**3d** afforded enantiomerically enriched product (*R*)-**2g**. The result shed some light on mechanism of the asymmetric induction by comparison with course of similar reactions, described in the literature (*vide infra*). Chiral thioureas containing a tertiary amino group are well-known organocatalysts applied to, for example, the addition of malonates to ω-nitrostyrenes (ArCH=CHNO_2_) with the parent dimethylamino derivative **3a** introduced by Takemoto et al. (Figure 1).^22^ Importantly, the same direction of asymmetric induction with (1*R*,2*R*)-**3a** is observed in the addition of malonates to ω-nitrostyrenes^22b^ and to β-arylethenesulfonyl fluorides (Table 1, entry 1), which suggests a similar mode of complexation and mechanism of action. In this context, NO_2_ and SO_2_ groups can be considered as *functional isosteres*(23) in accordance with early crystallographic studies,^24^ addition reactions to α,β-unsaturated sulfones,^25^ and enantioselective bond formation between ESF and indolone derivatives.^26^ Therefore, on the basis of mechanistic studies by Pápai et al.^27^ and Vetticat et al.^28^ on conjugate addition to nitroalkenes,^22b^ we reckon that bifunctional catalyst **3d** complexes malonate anion with two hydrogen bonds of the thiourea motif, whereas protonated amino group interacts with oxygen atom of the SO_2_F (Scheme 3, right).

**Scheme 3:**
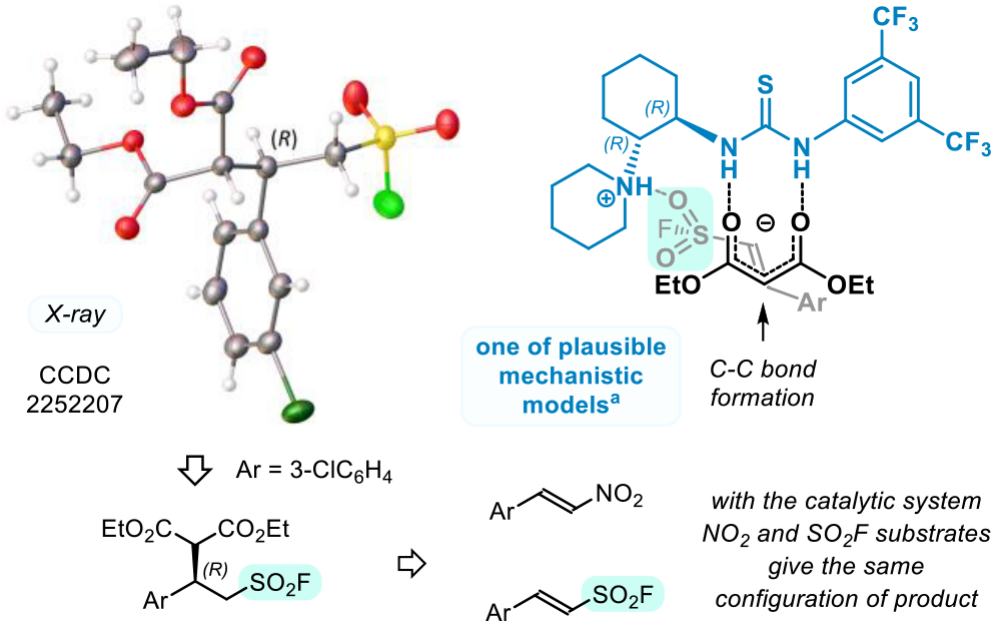
X-ray Structure of Adduct **2g** (Left) and One of the Plausible Mechanistic Models of the Observed Asymmetric Induction (Right) The mechanistic model was based on theoretical studies of enolate addition to ω-nitrostyrenes by Pápai et al.^27^ and Vetticat et al.^28^

In the last part of the project, we focused on atmospheric pressure addition reactions and postsynthetic functionalizations of chiral adduct **2a**. First, we tested model reaction of **1a** with diethyl malonate under atmospheric pressure and more vigorous conditions (Scheme 4, top). A stoichiometric amount of NEt_3_ in 0.5 M toluene solution was used at rt for 4 days, and *rac*-**2a** was formed in 34% yield, whereas the yield increased to a reasonable level of 70% at 60 °C. In turn, an organocatalyzed variant was tested with 20 mol % of thiourea **3d**, and the reaction time was extended to 14 days. In this case, we isolated adduct **2a** in 55% yield and 81% ee, but at a higher temperature (60 °C), the enantioselectivity decreased to 70% ee. The data demonstrate that malonate adducts can be synthesized under atmospheric pressure using high catalyst loading and prolonged reaction time. However, yields and enantioselectivities of products are questionable (considering, e.g., cost and molecular mass of **3d**), and substrate conversions remain incomplete in all cases.

**Scheme 4:**
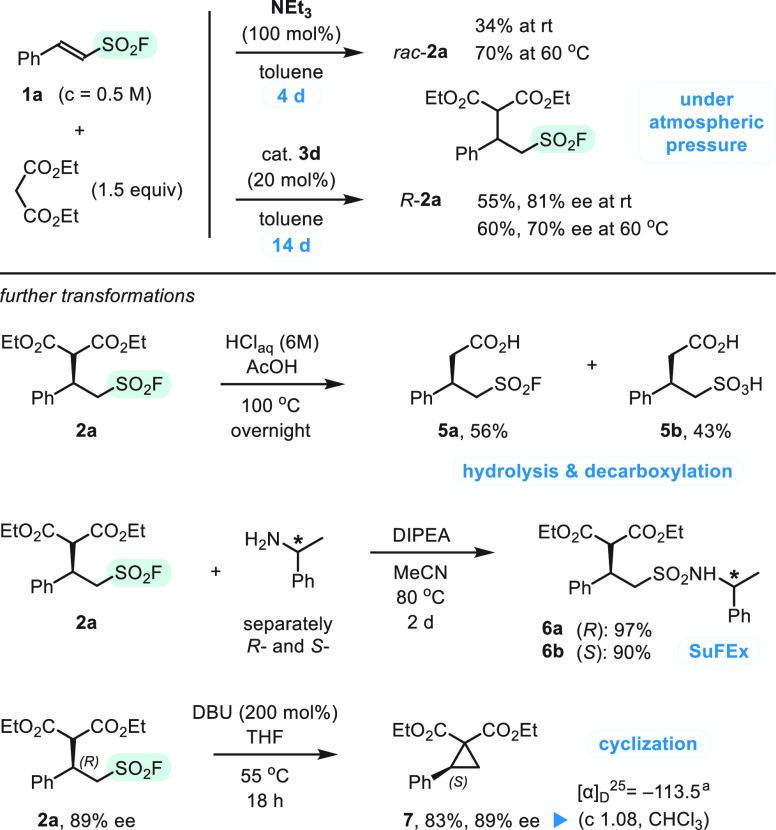
Atmospheric Pressure Addition Experiments (Top) and Follow-up Studies (Bottom) Optical rotation of **7** was consistent with the literature data of *S*-enantiomer.^30^

Then, we studied transformations of model adduct (*R*)-**2a** (Scheme 4, bottom). An attempt at decarboxylation with aqueous HCl in acetic acid solution at 100 °C gave a mixture of sulfonyl fluoride (**5a**, 56%) and sulfonic acid (**5b**, 43%), with both bearing a free carboxyl group. Synthesis of sulfonamides via SuFEx with (*R*)- and (*S*)-1-phenylethylamines (DIPEA, MeCN, 80 °C) gave diastereoisomeric sulfonamides **6a**,**b** isolated in 97% and 90% yield, respectively. Finally, inspired by a report of Qin et al.,^11^ we tested DBU-promoted cyclization via substitution of the fluorosulfonyl group.^29^ Interestingly, diethyl 2-phenylcyclopropane-1,1-dicarboxylate (**7**) was isolated in 83%, and its enantiomeric excess was essentially the same as that of the substrate **2a** (89% ee). Optical rotation of **7** was consistent with the literature data of *S*-enantiomer,^30^ thereby supporting a mechanism of nucleophilic substitution in which adjacent stereogenic center remains intact. The synthesis of chiral cyclopropane supplements metal-catalyzed carbene addition methods^30,31^ and is of particular interest because of numerous applications of the small ring structures in stereospecific expansion reactions.^32^

In conclusion, we presented reactions of β-arylethenesulfonyl fluorides with dialkyl malonates catalyzed with chiral amino-thiourea under high-pressure conditions (9 kbar). A set of 19 enantioenriched products was isolated in yields up to 96% and ee values up to 92%. Although unsaturated sulfonyl fluorides are less electrophilic than nitroalkenes^7,33^ and react at reasonable rates only under high-pressure conditions, mechanism of action of the organocatalyst is likely the same for both classes of the substrates. Importantly, the enantioselective addition of malonates to ArCH=CHSO_2_F delivers products potent for further transformations, such as SuFEx and cyclization to cyclopropanes.

## Data Availability

The data underlying this study are available in the published article and it is Supporting Information.

## Supporting Information Available

Experimental procedures, characterization data, and HPLC chromatograms and NMR spectra reproductions of the synthesized compounds (PDF)
