# Supplementary material for: Enantioselective Addition of Dialkyl Malonates to β-Arylethenesulfonyl Fluorides under High-Pressure Conditions
Source: Org Lett. 2023 Sep 1;25(37):6818–22. doi: 10.1021/acs.orglett.3c02302 (PMC10521026; doi:10.1021/acs.orglett.3c02302)

Enantioselective Addition of Dialkyl Malonates to β-Arylethenesulfonyl Fluorides under High-Pressure Conditions

Michał Kopyt,[1,2] Michał Tryniszewski,[1] Michał Barbasiewicz[1,*]
and Piotr Kwiatkowski[1,2,*]

[1]Faculty of Chemistry, University of Warsaw, Pasteura 1, 02-093 Warsaw, Poland
[2]Biological and Chemical Research Centre, University of Warsaw, Żwirki i Wigury 101, 02-089 Warsaw, Poland

Supporting Information

Table of Contents

General information 3

Preparation of substrates 4

Complete set of the optimization data with catalyst **3d** 6

Experiments carried out for a shortened time (2 h) 7

General Procedure 8

Analytical data of adducts **2b-r**, **4** 9

Products of further transformations of adduct **2a** 28

Single-crystal X-Ray diffraction analysis of **2g** (CCDC 2252207) 31

Reproductions of ^{1}H, ^{13}C, and ^{19}F NMR spectra 33

General information

All solvents were commercially-available and used as received, unless noted otherwise. Purification of products was performed using flash chromatography on silica gel (Merck Kieselgel 60, 230-400 mesh) with mixtures of hexanes : ethyl acetate, as eluent (typically hexanes to hexanes : ethyl acetate 85 : 15 gradient elution), unless noted otherwise. Thin-layer chromatography (TLC) was performed on silica gel plates (Merck Kieselgel 60 F_{254}). Visualization of the developed TLC plates was accomplished using UV light or *p*-anisaldehyde stain.

All NMR spectra were recorded in $CDCl_3$ or DMSO-d_6 using Agilent 400 MHz spectrometer. Chemical shifts of ^{1}H NMR and ^{13}C NMR are reported as δ values relative to TMS (δ=0.00) and $CDCl_3$ (δ=77.0), respectively. ^{19}F NMR spectra were recorded using hexafluorobenzene, as an internal standard (δ= -161.64 ppm), except for spectra of **1p**, which used trichlorofluoromethane, as a standard (δ=0.00). The following abbreviations are used to indicate multiplicity: s - singlet, d - doublet, t - triplet, q - quartet, m - multiplet**.**

Mass spectra were measured on a QExactive unit equipped with Orbitrap mass analyzer, using HR ESI technique. Optical rotation was recorded on a Perkin Elmer 241 polarimeter. Enantiomeric ratios were determined using high performance liquid chromatography (HPLC) techniques. HPLC analyses were performed on a Merck LabChrom chromatograph equipped with the diode-array detector (DAD) and Chiralpak® IB (25 cm × 0.46 cm, 5 μm) or Chiralpak® IC (25 cm × 0.46 cm, 5 μm) columns eluted with isopropanol : hexane. Comparative racemic samples of adducts **2a-r**, **4** for enantiomeric excess analysis were prepared using racemic catalyst **3d**.

High pressure experiments were performed at room temperature using a direct, single-stage piston-cylinder apparatus with a hydraulic press from Unipress (Warsaw, Poland) equipped with a liquid piston vessel LV/30/16 and laboratory hydraulic press U101. Experiments were conducted in 0.2, 0.5, 1.2, 2.1, and 3.0 mL Teflon ampoules inserted into the high-pressure vessel filled with hexanes, as a transmission medium.

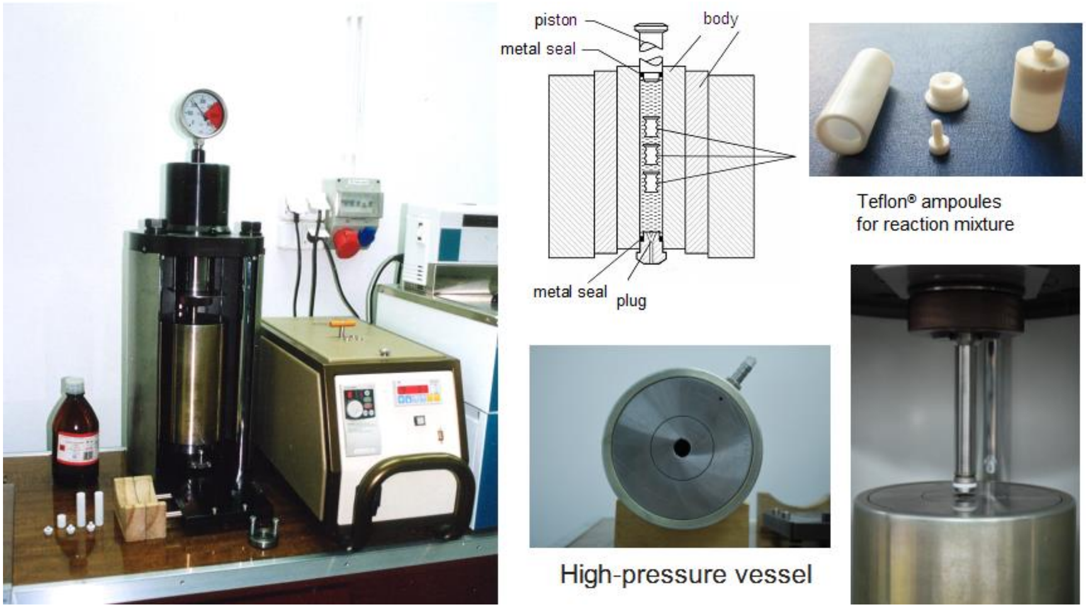

Preparation of substrates

Preparation β-arylethenesulfonyl fluorides, except of **1b**, **1m**, and **1p**, was reported in: M. Tryniszewski, D. Basiak, M. Barbasiewicz. *Org. Lett.* **2022**, *24*, 4270-4274.

3-Cyano-4-fluorophenylethenesulfonyl fluoride (**1p**) was obtained using the same method, but was not reported previosly. Analytical data is given below.

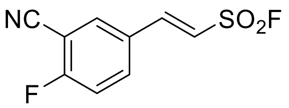

1p, (*E*)-2-(3-cyano-4-fluorophenyl)ethenesulfonyl fluoride, yield 29%, white solid, m.p. 121.5-122.5°C.

^{1}H NMR (400 MHz, $CDCl_3$) δ 7.86 (ddd, *J*=5.9, 2.4, 0.4 Hz, 1H), 7.82 (ddd, *J*=8.7, 4.9, 2.4 Hz, 1H), 7.76 (dd, *J*=15.6, 0.6 Hz, 1H), 7.38 (dd, *J*=8.7, 8.2 Hz, 1H), 6.90 (ddd, *J*=15.6, 2.6, 0.6 Hz, 1H).

^{19}F NMR (376 MHz, $CDCl_3$) δ 61.6 (d, *J*=2.7 Hz), -100.0 (m).

^{13}C NMR (100 MHz, $CDCl_3$) δ 164.8 (d, *J*=267 Hz), 144.7 (d, *J*=2.6 Hz), 135.1 (d, *J*=9.2 Hz), 134.1 (d, *J*=1.2 Hz), 128.3 (dd, *J*=4.0, 1.3 Hz), 120.8 (dd, *J*=29.5, 2.4 Hz), 118.0 (d, *J*=20.6 Hz), 112.5, 103.4 (d, *J*=16.6 Hz).

HRMS (ESI) $C_9H_5F_2NO_2S$ calcd 211.0103 [M+], found 211.0094 [M+].

4-Chlorophenyl- and (2-chlorophenyl)ethenesulfonyl fluorides (**1b**, and **1m**, respectively) were prepared via chlorosulfonation of styrenes, followed by halogen exchange (Cl→F). The procedures were referenced in: M. Tryniszewski, D. Basiak, M. Barbasiewicz. *Org. Lett.* **2022**, *24*, 4270-4274. Analytical data are given below.

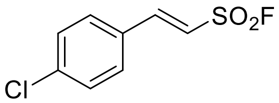

1b, (*E*)-2-(4-chlorophenyl)ethenesulfonyl fluoride, white crystals, m.p. 126.5-127.0°C (lit. 129-130°C; G.-F. Zha, Q. Zheng, J. Leng, P. Wu, H.-L. Qin, K. B. Sharpless. *Angew. Chem. Int. Ed.* **2017**, *56*, 4849-4852).

^{1}H NMR (400 MHz, $CDCl_3$) δ 7.74 (d, *J*=15.5 Hz, 1H), 7.51-7.40 (m, 4H), 6.84 (dd, *J*=15.5, 2.5 Hz, 1H).

^{19}F NMR (376 MHz, $CDCl_3$) δ 61.8.

^{13}C NMR (100 MHz, $CDCl_3$) δ 147.4 (d, *J*=2.7 Hz), 138.9, 130.2, 129.8, 129.4, 118.4 (d, *J*=28.4 Hz).

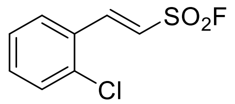

1m, (*E*)-2-(2-chlorophenyl)ethenesulfonyl fluoride, yellow oil.

^{1}**H NMR** (400 MHz, $CDCl_3$) δ 8.23 (ddd, *J*=15.6, 1.2, 0.6 Hz, 1H), 7.61 (dd, *J*=7.8, 1.6 Hz, 1H), 7.52-7.43 (m, 2H), 7.37 (tdd, *J*=7.2, 1.5, 0.6 Hz, 1H), 6.94 (dd, *J*=15.6, 2.5 Hz, 1H).

^{19}**F NMR** (376 MHz, $CDCl_3$) δ 61.5.

^{13}**C NMR** (100 MHz, $CDCl_3$) δ 144.6 (d, *J*=3.0 Hz), 135.9, 133.3, 130.8, 129.3 (d, *J*=1.4 Hz), 128.6, 127.5, 120.5 (d, *J*=28.7 Hz).

The ^{1}H, ^{13}C and ^{19}F NMR spectra were consistent with those described in the literature: X. Nie, T. Xu, J. Song, A. Devaraj, B. Zhang, Y. Chen, S. Liao. *Angew. Chem. Int. Ed.* **2021**, *60*, 3956-3960.

Complete set of the optimization data with catalyst 3d

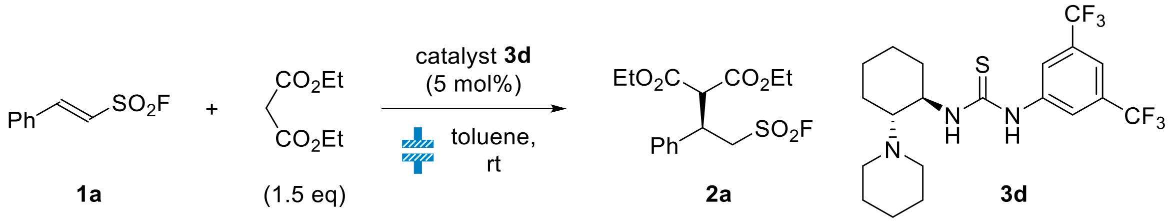

1a (1.5 eq) **2a** **3d**

Entry	mol% of cat. **3d**	Conc. of **1a** (mol/L)	Pressure	Time (h)	Conversion [%] (isolated yield)	ee [%] (HPLC)
1	5	1.0	9 kbar	20	>99%	84%
2	5	1.0	6 kbar	20	96%	86%
3	5	0.5	9 kbar	20	>99% (94%)	89%
4	5	0.5	9 kbar	2	98%	89%
5	5	0.5	6 kbar	2	39%	86%
6	5	0.5	1 bar	168	3.6%	-
7	5	0.2	9 kbar	20	89%	90%
8	2	1.0	9 kbar	20	>99%	85%
9	2	0.5	9 kbar	20	93%	89%
10	2	1.0	6 kbar	20	49%	85%

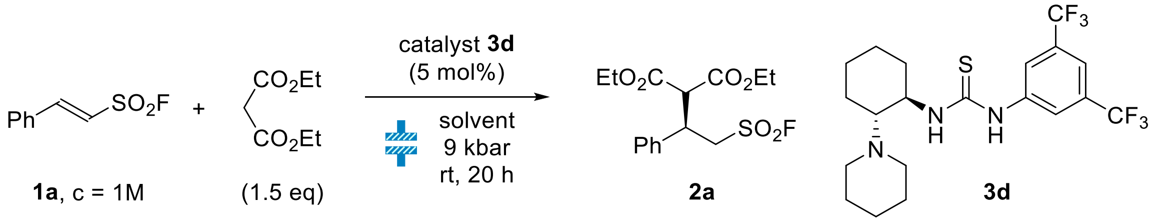

1a, c = 1M (1.5 eq) **2a** **3d**

Entry	Solvent	Conversion (NMR)	ee [%] (HPLC)
1	toluene	>99%	83 (*R*)
2	DCM	82%	78 (*R*)
3	THF	95%	71 (*R*)
4	2-MeTHF	98%	78 (*R*)
5	$PhCF_3$	78%	73 (*R*)

Experiments carried out for a shortened time (2 h)

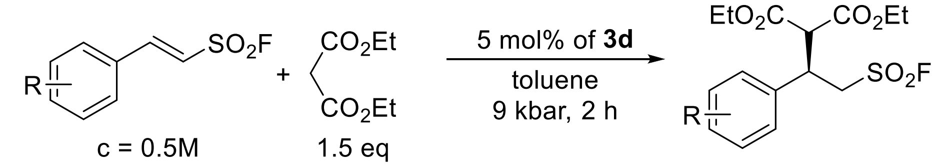

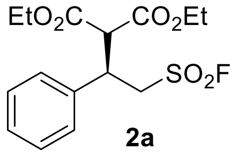

2a
98% conv., 89% ee

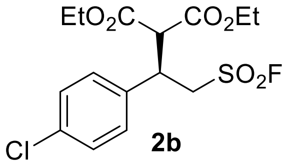

2b
94% conv., 89% ee

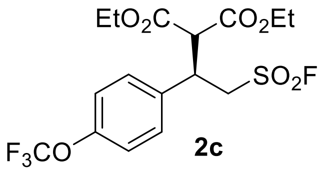

2c
97% conv., 88% ee

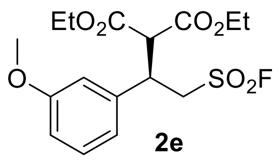

2e
93% conv., 85% ee

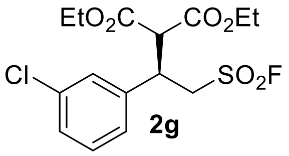

2g
>99% conv., 82% ee

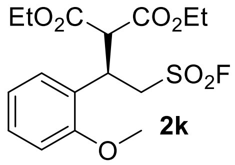

2k
91% conv., 91% ee

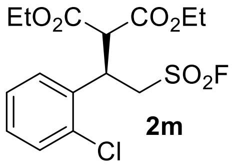

2m
>99% conv., 87% ee

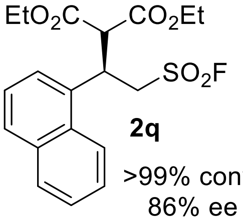

2q
>99% conv.
86% ee

General Procedure

A 2.1 ml teflon ampoule was loaded with thiourea catalyst **3d** (24 mg, 0.053 mmol, 5 mol%), sulfonyl fluoride **1a** (195 mg, 1.05 mmol) and diethyl malonate (252 mg, 1.57 mmol, 1.5 equiv), filled up with toluene, and subjected to 9 kbar of hydrostatic pressure for 20 h at rt. After decompression, the reaction mixture was directly purified using column chromatography on silica gel (hexanes : ethyl acetate 100 : 0 to 85 : 15) to obtain adduct **2a** (342 mg, 0.99 mmol, 94%, 89% ee), as a white solid.

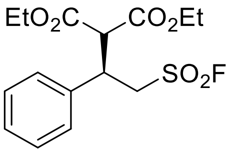

Compound **2a**

^{1}H NMR (400 MHz, $CDCl_3$) δ 7.37-7.25 (m, 5H), 4.23 (q, *J*=7.1 Hz, 1H), 4.21 (q, *J*=7.1 Hz, 1H), 4.13 (ddd, *J*=14.1, 6.6, 2.7 Hz, 1H), 4.08-3.92 (m, 4H), 3.77 (d, *J*=8.2 Hz, 1H), 1.24 (t, *J*=7.1 Hz, 3H), 1.06 (t, *J*=7.1 Hz, 3H).

^{19}F NMR (376 MHz, $CDCl_3$) δ 59.2 (dd, *J*=6.5, 1.5 Hz).

^{13}C NMR (100 MHz, $CDCl_3$) δ 167.4, 166.7, 136.5, 128.9, 128.5, 128.1, 62.2, 62.0, 56.4 (d, *J*=1.7 Hz), 53.9 (d, *J*=14.8 Hz), 40.6, 13.9, 13.7.

HRMS (ESI) $C_{15}H_{19}FO_6S$ calcd 347.0959 [M+H], found 347.0959 [M+H].

$[\alpha]_D^{25}$ = -0.29 (c 1.05, $CHCl_3$, 89% ee).

Enantiomeric excess was determined by HPLC analysis using a Chiralpak® IC column (hexane : i-PrOH 95 : 5, flow rate 1.0 mL/min, λ = 219 nm): ent-(S) t_r = 11.01 min and ent-(R) t_r = 17.92 min.

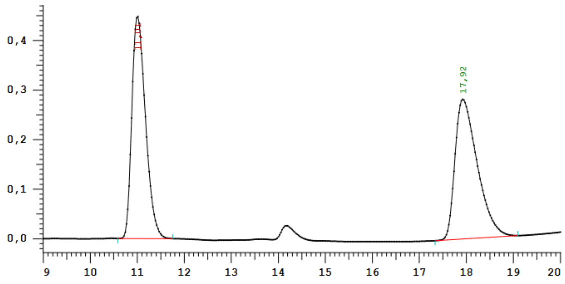

No.	RT	Area	Conc 1	BC
1	11,01	4521359	49,239	MC
2	17,92	4661161	50,761	BB
		9182520	100,000	

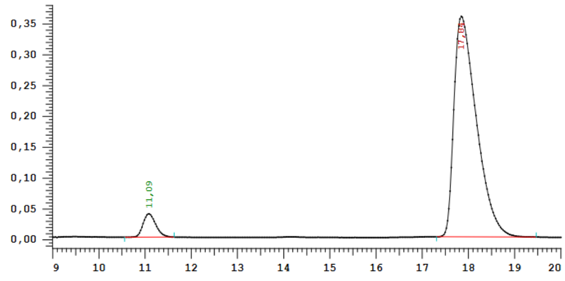

No.	RT	Area	Conc 1	BC
1	11,09	360882	5,607	BB
2	17,84	6075584	94,393	MC
		6436466	100,000	

Analytical data of adducts 2b-r, 4

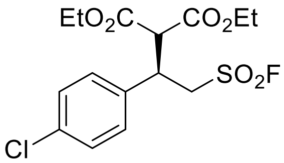

Product **2b** (412 mg, 1.08 mmol, 94%, 89% ee) was prepared according to the general procedure from sulfonyl fluoride **1b** (255 mg, 1.16 mmol), and isolated using column chromatography on silica gel (hexanes : ethyl acetate 100 : 0 to 85 : 15), as a white solid.

^{1}H NMR (400 MHz, $CDCl_3$) δ 7.34-7.30 (m, 2H), 7.25-7.21 (m, 2H), 4.23 (q, *J*=7.2 Hz, 1H), 4.21 (q, *J*=7.1 Hz, 1H), 4.14-3.99 (m, 4H), 3.92 (ddd, *J*=14.3, 10.2, 1.7 Hz, 1H), 3.73 (d, *J*=8.1 Hz, 1H), 1.25 (t, *J*=7.1 Hz, 3H), 1.11 (t, *J*=7.1 Hz, 3H).

^{19}F NMR (376 MHz, $CDCl_3$) δ 59.4 (dd, *J*=6.0, 2.0 Hz).

^{13}C NMR (100 MHz, $CDCl_3$) δ 167.2, 166.5, 135.0, 134.5, 129.5, 129.2, 62.3, 62.2, 56.2 (d, *J*=1.6 Hz), 53.7 (d, *J*=15.2 Hz), 40.0, 13.9, 13.8.

HRMS (ESI) $C_{15}H_{18}ClFO_6S$ calcd 381.0569 [M+H], found 381.0567 [M+H].

$[\alpha]_D^{25}$ = +2.1 (c 1.19, $CHCl_3$, 89% ee).

Enantiomeric excess was determined by HPLC analysis using a Chiralpak® IC column (hexane : i-PrOH 95 : 5, flow rate 1.0 mL/min, λ = 219 nm): ent-(S) t_r = 9.55 min and ent-(R) t_r = 14.21 min.

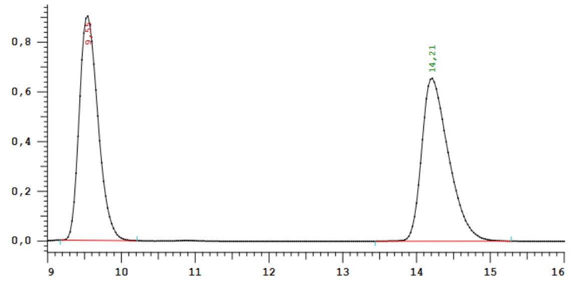

No.	RT	Area	Conc 1	BC
1	9,55	7967473	49,011	MC
2	14,21	8289073	50,989	BB
		16256546	100,000	

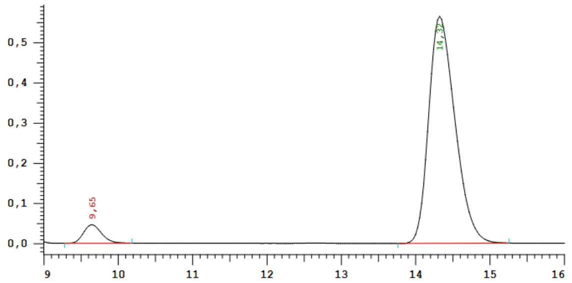

No.	RT	Area	Conc 1	BC
1	9,65	387298	5,301	MC
2	14,32	6918700	94,699	BB
		7305998	100,000	

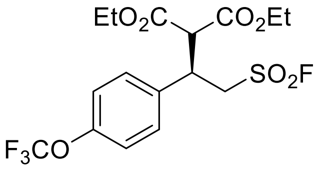

Product **2c** (203 mg, 0.47 mmol, 85%, 88% ee) was prepared according to the general procedure from sulfonyl fluoride **1c** (150 mg, 0.55 mmol), and isolated using column chromatography on silica gel (hexanes : ethyl acetate 100 : 0 to 85 : 15), as a yellow solid.

^{1}H NMR (400 MHz, $CDCl_3$) δ 7.35-7.31 (m, 2H), 7.21-7.16 (m, 2H), 4.27-3.99 (m, 6H), 3.93 (ddd, *J*=14.1, 9.9, 1.7 Hz, 1H), 3.75 (d, *J*=8.2 Hz, 1H), 1.23 (t, *J*=7.1 Hz, 3H), 1.05 (t, *J*=7.1 Hz, 3H).

^{19}F NMR (376 MHz, $CDCl_3$) δ 59.5 (dd, *J*=5.9, 2.0 Hz), -57.8.

^{13}C NMR (100 MHz, $CDCl_3$) δ 167.1, 166.5, 149.1 (q, *J*=1.7 Hz), 135.2, 129.8, 121.2, 120.3 (q, *J*=258 Hz), 62.3, 62.1, 56.2 (d, *J*=1.5 Hz), 53.8 (d, *J*=15.2 Hz), 40.0, 13.8, 13.6.

HRMS (ESI) $C_{16}H_{18}F_4O_7S$ calcd 431.0782 [M+H], found 431.0785 [M+H].

Enantiomeric excess was determined by HPLC analysis using a Chiralpak® IC column (hexane : i-PrOH 95 : 5, flow rate 1.0 mL/min, λ = 219 nm): ent-(S) t_r = 7.09 min and ent-(R) t_r = 10.75 min.

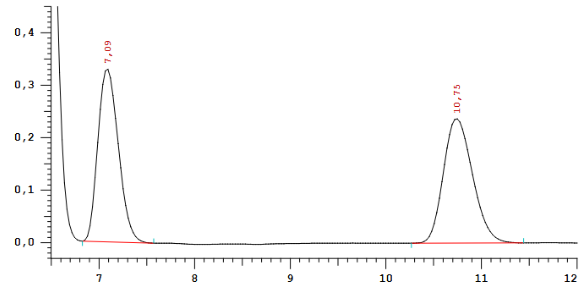

No.	RT	Area	Conc 1	BC
1	7,09	2459309	49,512	MC
2	10,75	2507833	50,488	MC

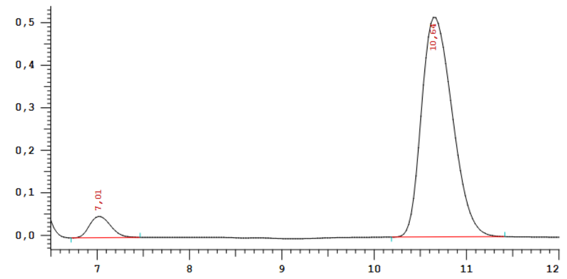

No.	RT	Area	Conc 1	BC
1	7,01	394572	6,184	MC
2	10,64	5986408	93,816	MC

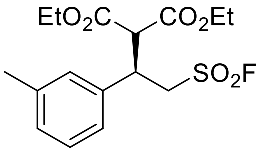

Product **2d** (362 mg, 1.00 mmol, 95%, 87% ee) was prepared according to the general procedure from sulfonyl fluoride **1d** (212 mg, 1.06 mmol), and isolated using column chromatography on silica gel (hexanes : ethyl acetate 100 : 0 to 85 : 15), as a white solid.

^{1}H NMR (400 MHz, $CDCl_3$) δ 7.25-7.20 (m, 1H), 7.13-7.05 (m, 3H), 4.22 (q, *J*=7.1 Hz, 1H), 4.21 (q, *J*=7.1 Hz, 1H), 4.15-3.92 (m, 5H), 3.76 (d, *J*=7.9 Hz, 1H), 2.33 (d, *J*=0.8 Hz, 3H), 1.25 (t, *J*=7.1 Hz, 3H), 1.09 (t, *J*=7.1 Hz, 3H).

^{19}F NMR (376 MHz, $CDCl_3$) 59.1 (dd, *J*=5.6, 1.5 Hz).

^{13}C NMR (100 MHz, $CDCl_3$) δ 167.4, 166.7, 138.6, 136.5, 129.2, 128.80, 128.78, 125.0, 62.1, 62.0, 56.5 (d, *J*=1.6 Hz), 53.9 (d, *J*=14.8 Hz), 40.5, 21.4, 13.9, 13.7.

HRMS (ESI) $C_{16}H_{21}FO_6S$ calcd 361.1116 [M+H], found 361.1115 [M+H].

$[\alpha]_D^{25}$ = +1.7 (c 1.11, $CHCl_3$, 87% ee).

Enantiomeric excess was determined by HPLC analysis using a Chiralpak® IC column (hexane : i-PrOH 95 : 5, flow rate 1.0 mL/min, λ = 219 nm): ent-(S) t_r = 9.95 min and ent-(R) t_r = 14.35 min.

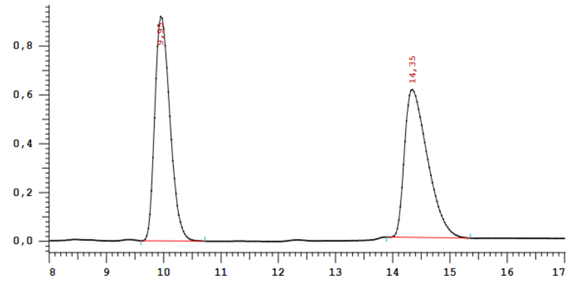

No.	RT	Area	Conc 1	BC
1	9,95	8493608	49,964	MC
2	14,35	8505798	50,036	MC
		16999406	100,000	

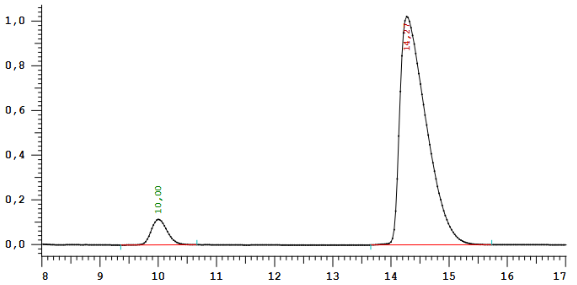

No.	RT	Area	Conc 1	BC
1	10,00	1090402	6,343	BB
2	14,27	16099774	93,657	MC
		17190176	100,000	

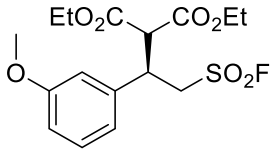

Product **2e** (354 mg, 0.94 mmol, 90%, 85% ee) was prepared according to the general procedure from sulfonyl fluoride **1e** (226 mg, 1.05 mmol), and isolated using column chromatography on silica gel (hexanes : ethyl acetate 100 : 0 to 80 : 20), as a white solid.

^{1}H NMR (400 MHz, $CDCl_3$) δ 7.26-7.23 (m, 1H), 6.88-6.79 (m, 3H), 4.23 (q, *J*=7.1 Hz, 1H), 4.21 (q, *J*=7.1 Hz, 1H), 4.15-3.92 (m, 5H), 3.78 (s, 3H), 3.76 (d, *J*=7.8 Hz, 1H), 1.25 (t, *J*=7.1 Hz, 3H), 1.10 (t, *J*=7.1 Hz, 3H).

^{19}F NMR (376 MHz, $CDCl_3$) δ 59.1 (dd, *J*=6.6, 1.0 Hz).

^{13}C NMR (100 MHz, $CDCl_3$) δ 167.4, 166.7, 159.8, 138.1, 130.0, 120.1, 114.2, 113.6, 62.2, 62.0, 56.4 (d, *J*=1.6 Hz), 55.2, 53.9 (d, *J*=15.0 Hz), 40.5, 13.9, 13.8.

HRMS (ESI) $C_{16}H_{21}FO_7S$ calcd 377.1065 [M+H], found 377.1064 [M+H].

$[\alpha]_D^{25}$ = +4.1 (c 1.03, $CHCl_3$, 85% ee).

Enantiomeric excess was determined by HPLC analysis using a Chiralpak® IB column (hexane : i-PrOH 95 : 5, flow rate 1.0 mL/min, λ = 219 nm): ent-(R) t_r = 11.55 min and ent-(S) t_r = 14.64 min.

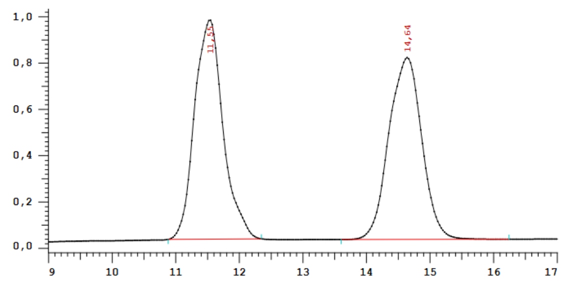

No.	RT	Area	Conc 1	BC
1	11,55	14706385	50,984	MC
2	14,64	14138798	49,016	MC
		28845183	100,000	

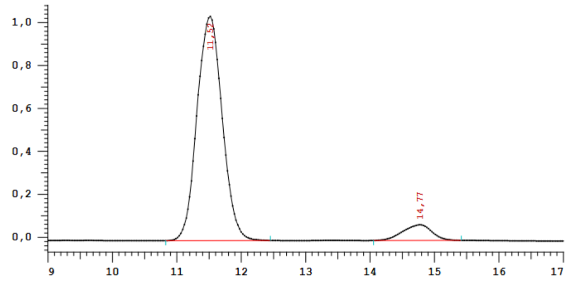

No.	RT	Area	Conc 1	BC
1	11,52	14175913	92,587	MC
2	14,77	1134973	7,413	MC
		15310886	100,000	

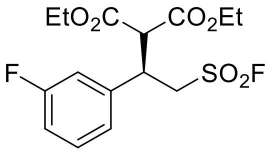

Product **2f** (355 mg, 0.97 mmol, 93%, 84% ee) was prepared according to the general procedure from sulfonyl fluoride **1f** (214 mg, 1.05 mmol), and isolated using column chromatography on silica gel (hexanes : ethyl acetate 100 : 0 to 85 : 15), as a white solid.

^{1}H NMR (400 MHz, $CDCl_3$) δ 7.33 (ddd, *J*=9.0, 7.7, 5.9 Hz, 1H), 7.08 (ddd, *J*=7.8, 1.6, 1.0 Hz, 1H), 7.05-6.99 (m, 2H), 4.24 (q, *J*=7.2 Hz, 1H), 4.23 (q, *J*=7.1 Hz, 1H), 4.16-4.02 (m, 4H), 3.98-3.90 (m, 1H), 3.76 (d, *J*=8.0 Hz, 1H), 1.25 (t, *J*=7.1 Hz, 3H), 1.12 (t, *J*=7.1 Hz, 3H).

^{19}F NMR (376 MHz, $CDCl_3$) δ 59.1 (dd, *J*=6.2, 1.8 Hz), -111.6 (td, *J*=8.9, 5.8 Hz).

^{13}C NMR (100 MHz, $CDCl_3$) δ 167.2, 166.5, 162.8 (d, *J*=247 Hz), 139.0 (d, *J*=7.0 Hz), 130.6 (d, *J*=8.3 Hz), 123.9 (d, *J*=3.0 Hz), 115.6 (d, *J*=21.0 Hz), 115.3 (d, *J*=22.3 Hz), 62.4, 62.2, 56.2 (d, *J*=1.6 Hz), 53.7 (d, *J*=15.3 Hz), 40.2 (d, *J*=1.9 Hz), 13.9, 13.8.

HRMS (ESI) $C_{15}H_{18}F_2O_6S$ calcd 365.0865 [M+H], found 365.0864 [M+H].

Enantiomeric excess was determined by HPLC analysis using a Chiralpak® IC column (hexane : i-PrOH 95 : 5, flow rate 1.0 mL/min, λ = 219 nm): ent-(S) t_r = 9.23 min and ent-(R) t_r = 12.91 min.

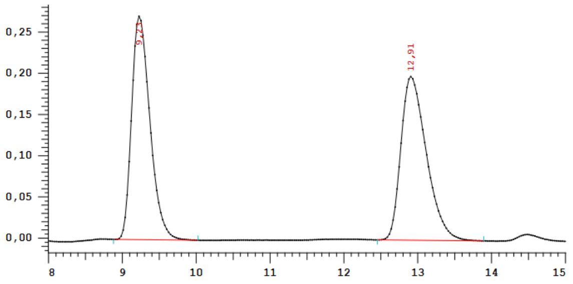

No.	RT	Area	Conc 1	BC
1	9,23	2334691	49,483	MC
2	12,91	2383444	50,517	MC
		4718135	100,000	

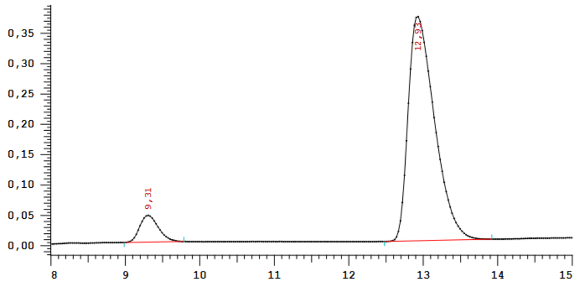

No.	RT	Area	Conc 1	BC
1	9,31	383358	7,758	MC
2	12,93	4557970	92,242	MC
		4941328	100,000	

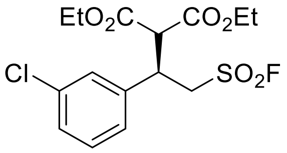

Product **2g** (213 mg, 0.56 mmol, 91%, 82% ee) was prepared according to the general procedure from sulfonyl fluoride **1g** (135 mg, 0.61 mmol), and isolated using column chromatography on silica gel (hexanes : ethyl acetate 100 : 0 to 85 : 15), as a white solid.

^{1}H NMR (400 MHz, $CDCl_3$) δ 7.31-7.28 (m, 3H), 7.21-7.17 (m, 1H), 4.24 (q, *J*=7.2 Hz, 1H), 4.23 (q, *J*=7.1 Hz, 1H), 4.16-3.91 (m, 5H), 3.75 (d, *J*=7.8 Hz, 1H), 1.26 (t, *J*=7.1 Hz, 3H), 1.12 (t, *J*=7.1 Hz, 3H).

^{19}F NMR (376 MHz, $CDCl_3$) δ 59.3 (dd, *J*=6.5, 1.9 Hz).

^{13}C NMR (100 MHz, $CDCl_3$) δ 167.1, 166.5, 138.6, 134.8, 130.2, 128.8, 128.3, 126.4, 62.4, 62.2, 56.1 (d, *J*=1.5 Hz), 53.6 (d, *J*=15.3 Hz), 40.2, 13.9, 13.8 (CH_3).

HRMS (ESI) $C_{15}H_{18}ClFO_6S$ calcd 381.0569 [M+H], found 381.0571 [M+H].

$[\alpha]_D^{25}$ = +1.35 (c 0.96, $CHCl_3$, 82% ee).

Enantiomeric excess was determined by HPLC analysis using a Chiralpak® IC column (hexane : i-PrOH 95 : 5, flow rate 1.0 mL/min, λ = 219 nm): ent-(S) t_r = 8.56 min and ent-(R) t_r = 10.85 min.

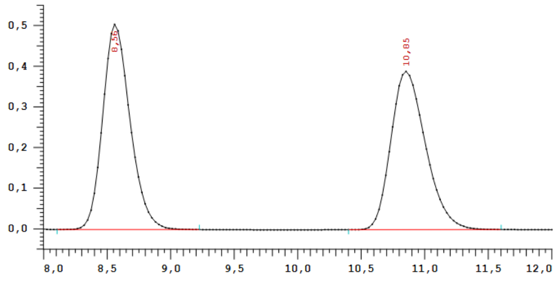

No.	RT	Area	Conc 1
1	8,56	3799398	50,680
2	10,85	3697407	49,320
		7496805	100,000

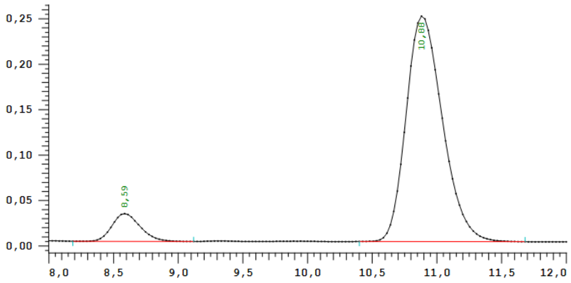

No.	RT	Area	Conc 1
1	8,59	232337	8,728
2	10,88	2429679	91,272
		2662016	100,000

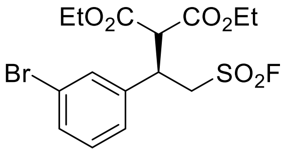

Product **2h** (194 mg, 0.46 mmol, 83%, 82% ee) was prepared according to the general procedure from sulfonyl fluoride **1h** (145 mg, 0.55 mmol), and isolated using column chromatography on silica gel (hexanes : ethyl acetate 100 : 0 to 85 : 15), as a white solid.

^{1}H NMR (400 MHz, $CDCl_3$) δ 7.48-7.42 (m, 2H), 7.26-7.21 (m, 2H), 4.24 (q, *J*=7.2 Hz, 1H), 4.23 (q, *J*=7.1 Hz, 1H), 4.16-3.91 (m, 5H), 3.75 (d, *J*=7.7 Hz, 1H), 1.26 (t, *J*=7.1 Hz, 3H), 1.12 (t, *J*=7.1 Hz, 3H).

^{19}F NMR (376 MHz, $CDCl_3$) δ 59.2 (d, *J*=6.3 Hz).

^{13}C NMR (100 MHz, $CDCl_3$) δ 167.1, 166.4, 138.9, 131.7, 131.2, 130.5, 126.9, 122.9, 62.4, 62.2, 56.1 (d, *J*=1.5 Hz), 53.5 (d, *J*=15.4 Hz), 40.2, 13.9, 13.8.

HRMS (ESI) $C_{15}H_{18}BrFO_6S$ calcd 425.0064 [M+H], 427.0044 [M+H], found 425.0069 [M+H], 427.0050 [M+H].

Enantiomeric excess was determined by HPLC analysis using a Chiralpak® IC column (hexane : i-PrOH 95 : 5, flow rate 1.0 mL/min, λ = 219 nm): ent-(S) t_r = 8.91 min and ent-(R) t_r = 11.23 min.

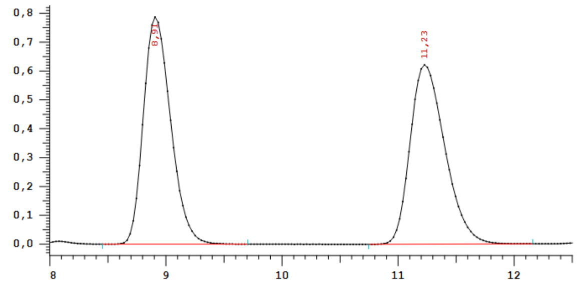

No.	RT	Area	Conc 1	BC
1	8,91	6426292	49,870	MC
2	11,23	6459693	50,130	MC
		12885985	100,000	

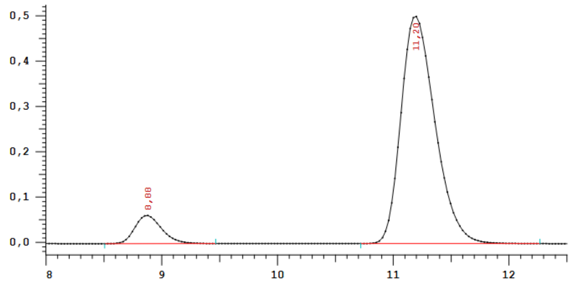

No.	RT	Area	Conc 1	BC
1	8,88	497669	8,827	MC
2	11,20	5140231	91,173	MC
		5637900	100,000	

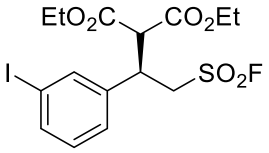

Product **2i** (476 mg, 1.01 mmol, 96%, 80% ee) was prepared according to the general procedure from sulfonyl fluoride **1i** (327 mg, 1.05 mmol), and isolated using column chromatography on silica gel (hexanes : ethyl acetate 100 : 0 to 85 : 15), as a white solid.

^{1}H NMR (400 MHz, $CDCl_3$) δ 7.68-7.60 (m, 2H), 7.29-7.26 (m, 1H), 7.09 (td, *J*=7.8, 0.4 Hz, 1H), 4.24 (q, *J*=7.2 Hz, 1H), 4.23 (q, *J*=7.1 Hz, 1H), 4.14-4.04 (m, 3H), 4.01-3.89 (m, 2H), 3.76-3.72 (m, 1H), 1.26 (t, *J*=7.1 Hz, 3H), 1.13 (t, *J*=7.1 Hz, 3H).

^{19}F NMR (376 MHz, $CDCl_3$) δ 59.2 (dd, *J*=5.9, 2.2 Hz).

^{13}C NMR (100 MHz, $CDCl_3$) δ 167.1, 166.5, 138.9, 137.6, 137.0, 130.6, 127.5, 94.6, 62.4, 62.2, 56.1 (d, *J*=1.5 Hz), 53.5 (d, *J*=15.4 Hz), 40.0, 14.0, 13.8.

HRMS (ESI) $C_{15}H_{18}FIO_6S$ calcd 472.9926 [M+H], found 472.9930 [M+H].

Enantiomeric excess was determined by HPLC analysis using a Chiralpak® IC column (hexane : i-PrOH 95 : 5, flow rate 1.0 mL/min, λ = 219 nm): ent-(S) t_r = 10.19 min and ent-(R) t_r = 13.63 min.

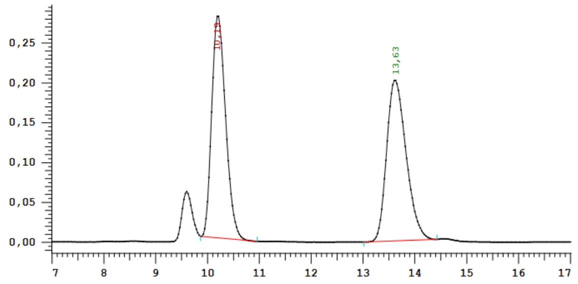

No.	RT	Area	Conc 1	BC
1	10,19	2592936	49,700	MC
2	13,63	2624287	50,300	BB
		5217223	100,000	

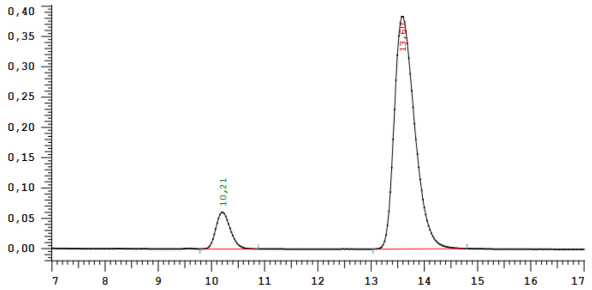

No.	RT	Area	Conc 1	BC
1	10,21	570491	10,034	BB
2	13,60	5114941	89,966	MC
		5685432	100,000	

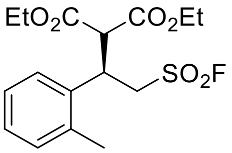

Product **2j** (195 mg, 0.54 mmol, 90%, 88% ee) was prepared according to the general procedure from sulfonyl fluoride **1j** (120 mg, 0.60 mmol), and isolated using column chromatography on silica gel (hexanes : ethyl acetate 100 : 0 to 85 : 15), as a yellow oil.

^{1}H NMR (400 MHz, $CDCl_3$) δ 7.20-7.17 (m, 4H), 4.39 (ddd, *J*=10.6, 8.7, 2.9 Hz, 1H), 4.24 (q, *J*=7.1 Hz, 1H), 4.23 (q, *J*=7.1 Hz, 1H), 4.11 (ddd, *J*=14.8, 6.5, 2.9 Hz, 1H), 4.06-3.90 (m, 3H), 3.71 (d, *J*=8.7 Hz, 1H), 2.44 (s, 3H), 1.26 (t, *J*=7.1 Hz, 3H), 1.04 (t, *J*=7.1 Hz, 3H).

^{19}F NMR (376 MHz, $CDCl_3$) δ 58.7 (dd, *J*=6.9, 1.7 Hz).

^{13}C NMR (100 MHz, $CDCl_3$) δ 167.5, 166.7, 136.9, 134.9, 131.2, 128.1, 126.5, 126.3, 62.2, 62.0, 56.2 (d, *J*=1.7 Hz), 54.0 (d, *J*=14.6 Hz), 35.3, 19.5, 13.9, 13.6.

HRMS (ESI) $C_{16}H_{21}FO_6S$ calcd 361.1116 [M+H], found 361.1115 [M+H].

Enantiomeric excess was determined by HPLC analysis using a Chiralpak® IC column (hexane : i-PrOH 95 : 5, flow rate 1.0 mL/min, λ = 219 nm): ent-(S) t_r = 8.75 min and ent-(R) t_r = 13.97 min.

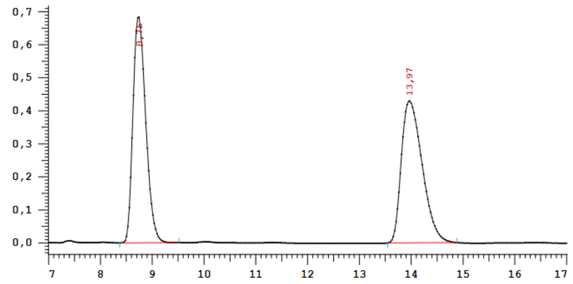

No.	RT	Area	Conc 1	BC
1	8,75	5870922	49,290	MC
2	13,97	6039954	50,710	MC
		11910876	100,000	

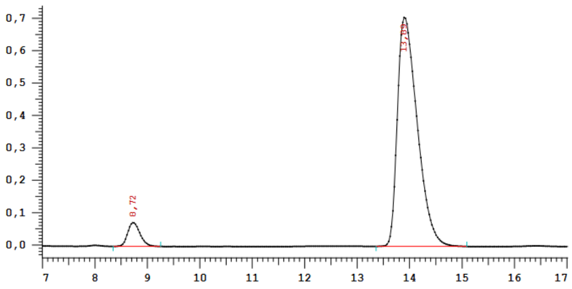

No.	RT	Area	Conc 1	BC
1	8,72	600942	6,039	MC
2	13,89	9349912	93,961	MC
		9950854	100,000	

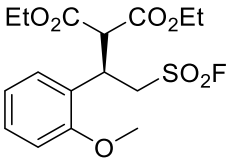

Product **2k** (361 mg, 0.96 mmol, 92%, 92% ee) was prepared according to the general procedure from sulfonyl fluoride **1k** (226 mg, 1.05 mmol), and isolated using column chromatography on silica gel (hexanes : ethyl acetate 100 : 0 to 80 : 20), as a yellow solid.

^{1}H NMR (400 MHz, $CDCl_3$) δ 7.31-7.24 (m, 1H), 7.20-7.16 (m, 1H), 6.92-6.86 (m, 2H), 4.28-4.17 (m, 3H), 4.13-4.09 (m, 2H), 4.02-3.95 (m, 1H), 3.93-3.86 (m, 5H), 1.26 (t, *J*=7.1 Hz, 3H), 0.97 (t, *J*=7.1 Hz, 3H).

^{19}F NMR (376 MHz, $CDCl_3$) δ 58.5 (dd, *J*=6.9, 2.2 Hz).

^{13}C NMR (100 MHz, $CDCl_3$) δ 167.8, 166.8, 157.5, 131.6, 129.9, 123.4, 120.8, 111.1, 62.1, 61.6, 55.4, 53.9 (d, *J*=1.6 Hz), 52.0 (d, *J*=14.5 Hz), 38.8, 14.0, 13.7.

HRMS (ESI) $C_{16}H_{21}FO_7S$ calcd 377.1065 [M+H], found 377.1064 [M+H].

$[\alpha]_D^{25}$ = -28.2 (c 1.10, $CHCl_3$, 92% ee).

Enantiomeric excess was determined by HPLC analysis using a Chiralpak® IC column (hexane : i-PrOH 90 : 10, flow rate 1.0 mL/min, λ = 219 nm): ent-(S) t_r = 8.72 min and ent-(R) t_r = 13.60 min.

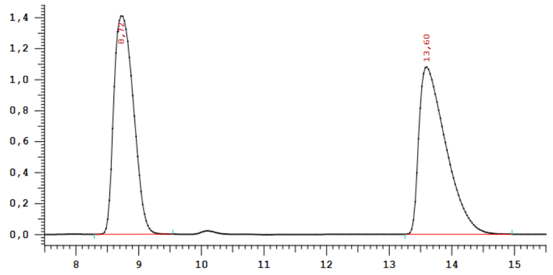

No.	RT	Area	Conc 1	BC
1	8,72	15385936	48,813	MC
2	13,60	16134268	51,187	MC
		31520204	100,000	

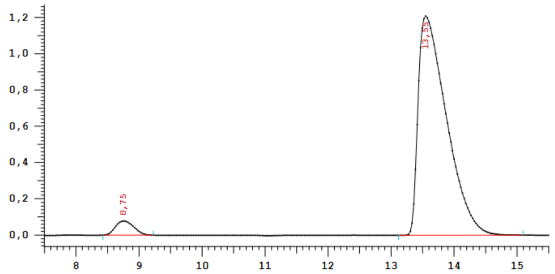

No.	RT	Area	Conc 1	BC
1	8,75	794622	4,040	MC
2	13,55	18873524	95,960	MC
		19668146	100,000	

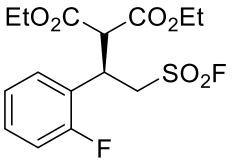

Product **2l** (188 mg, 0.52 mmol, 86%, 87% ee) was prepared according to the general procedure from sulfonyl fluoride **1l** (122 mg, 0.60 mmol), and isolated using column chromatography on silica gel (hexanes : ethyl acetate 100 : 0 to 85 : 15), as a yellow solid.

^{1}H NMR (400 MHz, $CDCl_3$) δ 7.35-7.26 (m, 2H), 7.15-7.04 (m, 2H), 4.30-4.17 (m, 3H), 4.09 (ddd, *J*=14.8, 6.0, 3.3 Hz, 1H), 4.05-3.95 (m, 3H), 3.92 (d, *J*=9.7 Hz, 1H), 1.27 (td, *J*=7.1, 0.4 Hz, 3H), 1.03 (td, *J*=7.1, 0.4 Hz, 3H).

^{19}F NMR (376 MHz, $CDCl_3$) δ 58.7 (dd, *J*=5.7, 2.5 Hz), -115.4 – -115.5 (m).

^{13}C NMR (100 MHz, $CDCl_3$) δ 167.2, 166.4, 161.1 (d, *J*=247 Hz), 131.2 (d, *J*=4.3 Hz), 130.5 (d, *J*=8.8 Hz), 124.6 (d, *J*=3.4 Hz), 123.0 (d, *J*=12.9 Hz), 116.0 (d, *J*=21.8 Hz), 62.4, 62.0, 54.6 (t, *J*=2.0 Hz), 52.5 (dd, *J*=15.6, 3.2 Hz), 36.8, 13.9, 13.7.

HRMS (ESI) $C_{15}H_{18}F_2O_6S$ calcd 365.0865 [M+H], found 365.0865 [M+H].

Enantiomeric excess was determined by HPLC analysis using a Chiralpak® IC column (hexane : i-PrOH 95 : 5, flow rate 1.0 mL/min, λ = 206 nm): ent-(S) t_r = 9.76 min and ent-(R) t_r = 14.48 min.

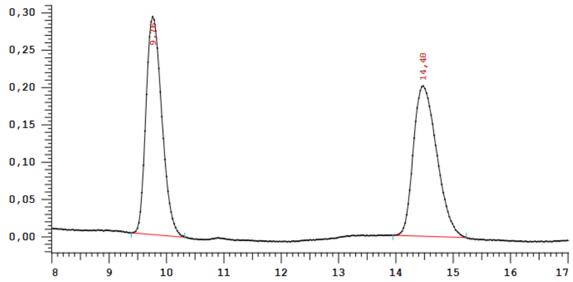

No.	RT	Area	Conc 1	BC
1	9,76	2799117	49,461	MC
2	14,48	2860112	50,539	MC
		5659229	100,000	

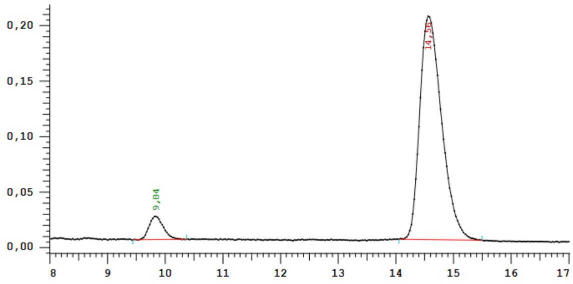

No.	RT	Area	Conc 1	BC
1	9,84	179023	6,324	BB
2	14,56	2651836	93,676	MC
		2830859	100,000	

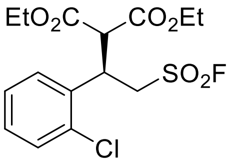

Product **2m** (350 mg, 0.92 mmol, 88%, 89% ee) was prepared according to the general procedure from sulfonyl fluoride **1m** (231 mg, 1.05 mmol), and isolated using column chromatography on silica gel (hexanes : ethyl acetate 100 : 0 to 85 : 15), as a yellow oil.

^{1}H NMR (400 MHz, $CDCl_3$) δ 7.43-7.39 (m, 1H), 7.35-7.32 (m, 1H), 7.26 (dd, *J*=6.0, 3.4 Hz, 2H), 4.58 (d, *J*=12.9 Hz, 1H), 4.29-4.16 (m, 3H), 4.15-4.02 (m, 4H), 1.24 (t, *J*=7.1 Hz, 3H), 1.11 (t, *J*=7.1 Hz, 3H).

^{19}F NMR (376 MHz, $CDCl_3$) δ 58.8 (t, *J*=4.0 Hz).

^{13}C NMR (100 MHz, $CDCl_3$) δ 167.3, 166.6, 133.9, 133.6, 130.5, 129.7, 127.2, 62.3, 62.1, 54.2, 51.9 (d, *J*=15.6 Hz), 37.3, 13.9, 13.7.

HRMS (ESI) $C_{15}H_{18}ClFO_6S$ calcd 381.0569 [M+H], found 381.0570 [M+H].

Enantiomeric excess was determined by HPLC analysis using a Chiralpak® IC column (hexane : i-PrOH 95 : 5, flow rate 1.0 mL/min, λ = 206 nm): ent-(S) t_r = 9.28 min and ent-(R) t_r = 19.84 min.

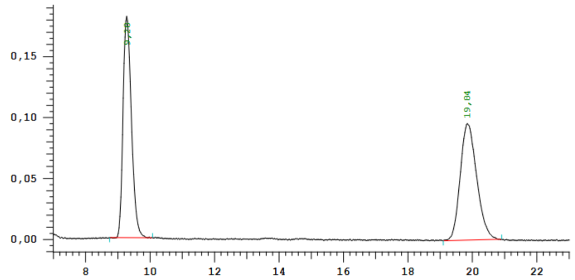

No.	RT	Area	Conc 1	BC
1	9,28	1647008	49,138	BB
2	19,84	1704765	50,862	BB
		3351773	100,000	

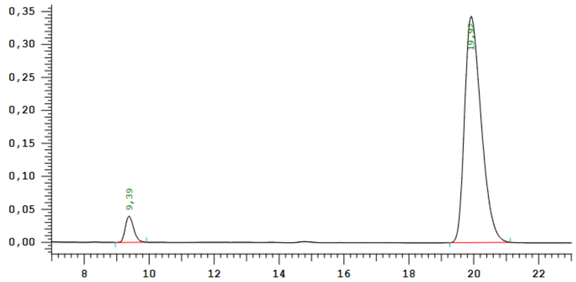

No.	RT	Area	Conc 1	BC
1	9,39	351618	5,418	BB
2	19,92	6138324	94,582	BB
		6489942	100,000	

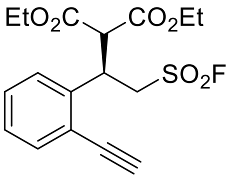

Product **2n** (205 mg, 0.55 mmol, 93%, 87% ee) was prepared according to the general procedure from sulfonyl fluoride **1n** (125 mg, 0.60 mmol), and isolated using column chromatography on silica gel (hexanes : ethyl acetate 100 : 0 to 85 : 15), as a white solid.

^{1}H NMR (400 MHz, $CDCl_3$) δ 7.54 (ddd, *J*=7.5, 1.5, 0.7 Hz, 1H), 7.37-7.26 (m, 3H), 4.57-4.48 (m, 1H), 4.36 (ddd, *J*=14.9, 10.3, 2.9 Hz, 1H), 4.29-4.16 (m, 3H), 4.12 (ddd, *J*=15.0, 5.8, 2.9 Hz, 1H), 4.04 (q, *J*=7.1 Hz, 1H), 4.03 (q, *J*=7.1 Hz, 1H), 3.47 (s, 1H), 1.24 (t, *J*=7.1 Hz, 3H), 1.08 (t, *J*=7.1 Hz, 3H).

^{19}F NMR (376 MHz, $CDCl_3$) δ 58.6 (dd, *J*=5.7, 3.0 Hz).

^{13}C NMR (100 MHz, $CDCl_3$) δ 167.5, 166.7, 138.1, 134.1, 129.3, 128.2, 121.5, 83.9, 81.1, 62.2, 62.0, 54.5, 52.2 (d, *J*=15.5 Hz), 39.0, 13.9, 13.7.

HRMS (ESI) $C_{17}H_{19}FO_6S$ calcd 371.0959 [M+H], found 371.0960 [M+H].

$[\alpha]_D^{25}$ = -8.3 (c 1.17, $CHCl_3$, 87% ee).

Enantiomeric excess was determined by HPLC analysis using a Chiralpak® IC column (hexane : i-PrOH 95 : 5, flow rate 1.0 mL/min, λ = 219 nm): ent-(S) t_r = 9.68 min and ent-(R) t_r = 18.45 min.

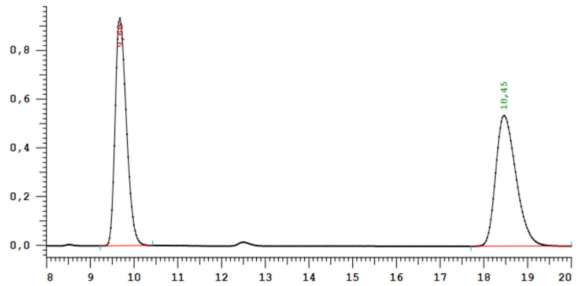

No.	RT	Area	Conc 1	BC
1	9,68	8450360	49,378	MC
2	18,45	8663141	50,622	BB
		17113501	100,000	

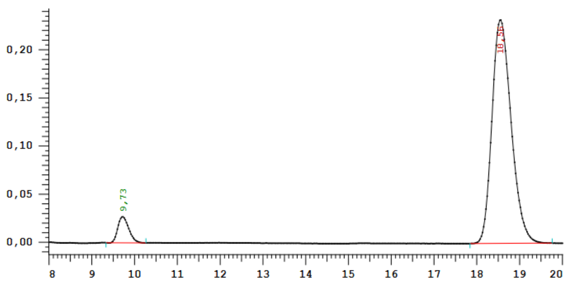

No.	RT	Area	Conc 1	BC
1	9,73	240534	6,223	BB
2	18,56	3624488	93,777	MC
		3865022	100,000	

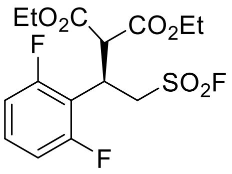

Product **2o** (213 mg, 0.56 mmol, 92%, 73% ee) was prepared according to the general procedure from sulfonyl fluoride **1o** (134 mg, 0.60 mmol), and isolated using column chromatography on silica gel (hexanes : ethyl acetate 100 : 0 to 85 : 15), as an yellow oil.

^{1}H NMR (400 MHz, $CDCl_3$) δ 7.34-7.27 (m, 1H), 6.92 (t, *J*=8.5 Hz, 2H), 4.56 (dddt, *J*=11.6, 10.6, 2.9, 1.4 Hz, 1H), 4.29 (q, *J*=7.2 Hz, 1H), 4.27 (q, *J*=7.1 Hz, 1H), 4.09 (ddd, *J*=14.7, 4.9, 3.0 Hz, 1H), 4.02-3.89 (m, 4H), 1.30 (t, *J*=7.1 Hz, 3H), 1.04 (t, *J*=7.1 Hz, 3H).

^{19}F NMR (376 MHz, $CDCl_3$) δ 57.2 (t, *J*=4.4 Hz), -112.0 – -112.1 (m).

^{13}C NMR (100 MHz, $CDCl_3$) δ 167.0, 166.1, 161.3 (d, *J*=249 Hz), 130.7 (t, *J*=10.8 Hz), 112.3 (t, *J*=17.1 Hz), 112.1-111.9 (m), 111.8-111.7 (m, CH), 62.5, 62.1, 54.0 (d, *J*=1.9 Hz), 51.7 (d, *J*=16.6 Hz), 30.2 (t, *J*=1.6 Hz), 13.9, 13.6.

HRMS (ESI) $C_{15}H_{17}F_3O_6S$ calcd 383.0771 [M+H], found 383.0770 [M+H].

Enantiomeric excess was determined by HPLC analysis using a Chiralpak® IC column (hexane : i-PrOH 95 : 5, flow rate 1.0 mL/min, λ = 206 nm): ent-(S) t_r = 12.43 min and ent-(R) t_r = 13.55 min.

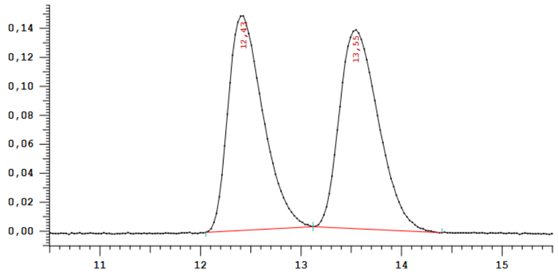

No.	RT	Area	Conc 1	BC
1	12,43	1791578	49,137	MC
2	13,55	1854526	50,863	MC
		3646104	100,000	

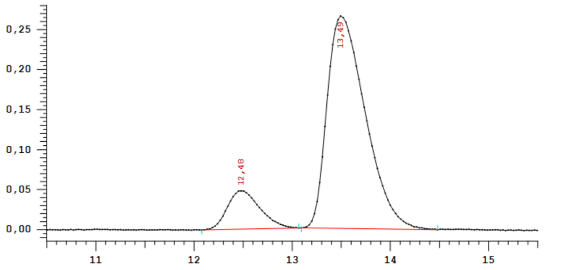

No.	RT	Area	Conc 1	BC
1	12,48	551745	13,270	MC
2	13,49	3605969	86,730	MC
		4157714	100,000	

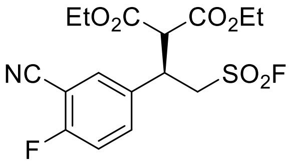

Product **2p** (185 mg, 0.48 mmol, 78%, 86% ee) was prepared according to the general procedure from sulfonyl fluoride **1p** (139 mg, 0.61 mmol), and isolated using column chromatography on silica gel (hexanes : ethyl acetate 100 : 0 to 80 : 20), as a white solid.

^{1}H NMR (400 MHz, $CDCl_3$) δ 7.65-7.56 (m, 2H), 7.28-7.21 (m, 1H), 4.30-4.19 (m, 2H), 4.16-4.03 (m, 4H), 3.99-3.90 (m, 1H), 3.76 (d, *J*=7.8 Hz, 1H), 1.27 (t, *J*=7.1 Hz, 3H), 1.15 (t, *J*=7.1 Hz, 3H).

^{19}F NMR (376 MHz, $CDCl_3$) δ 59.6 (dd, *J*=5.4, 2.2 Hz), -105.9 – -106.0 (m).

^{13}C NMR (100 MHz, $CDCl_3$) δ 166.8, 166.2, 162.8 (d, *J*=261 Hz), 135.3 (d, *J*=8.5 Hz), 133.9 (d, *J*=3.9 Hz), 133.4, 117.2 (d, *J*=19.9 Hz), 113.3, 102.2 (d, *J*=15.9 Hz), 62.6, 62.5, 55.8, 53.4 (d, *J*=15.9 Hz), 39.6, 13.9, 13.8.

HRMS (ESI) $C_{16}H_{17}F_2NO_6S$ calcd 390.0817 [M+H] 390.0816 [M+H].

$[\alpha]_D^{25}$ = +2.0 (c 1.20, $CHCl_3$, 86% ee).

Enantiomeric excess was determined by HPLC analysis using a Chiralpak® IC column (hexane : i-PrOH 90 : 10, flow rate 1.0 mL/min, λ = 219 nm): ent-(S) t_r = 16.51 min and ent-(R) t_r = 17.87 min.

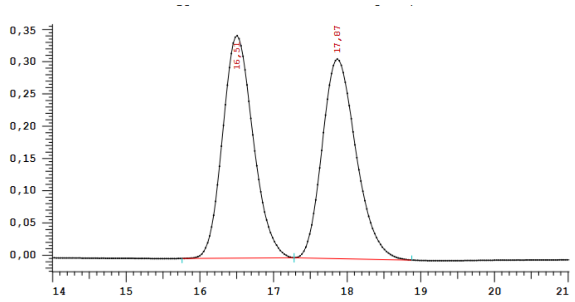

No.	RT	Area	Conc 1	BC
1	16,51	4984055	50,134	MC
2	17,87	4957332	49,866	MC
		9941387	100,000	

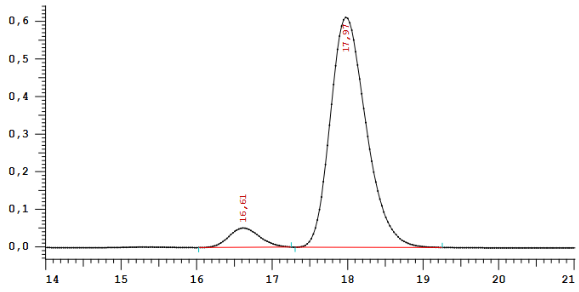

No.	RT	Area	Conc 1	BC
1	16,61	732336	6,752	MC
2	17,97	10114015	93,248	MC
		10846351	100,000	

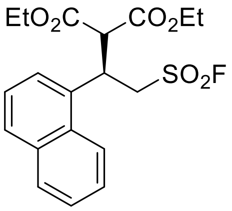

Product **2q** (401 mg, 1.01 mmol, 96%, 86% ee) was prepared according to the general procedure from sulfonyl fluoride **1q** (249 mg, 1.05 mmol), and isolated using column chromatography on silica gel (hexanes : ethyl acetate 100 : 0 to 85 : 15), as a colourless oil.

^{1}H NMR (400 MHz, $CDCl_3$) δ 8.16 (d, *J*=8.6 Hz, 1H), 7.88 (ddd, *J*=8.1, 1.4, 0.7 Hz, 1H), 7.85-7.80 (m, 1H), 7.61 (ddd, *J*=8.5, 6.8, 1.5 Hz, 1H), 7.55-7.44 (m, 3H), 5.05 (s, 1H), 4.31-4.27 (m, 2H), 4.25-4.15 (m, 2H), 3.99 (dt, *J*=16.5, 10.1 Hz, 3H), 1.20 (t, *J*=7.1 Hz, 3H), 0.98 (t, *J*=7.4 Hz, 3H).

^{19}F NMR (376 MHz, $CDCl_3$) δ 59.3.

^{13}C NMR (100 MHz, $CDCl_3$) δ 167.5, 166.8, 134.0, 132.5, 130.9, 129.2, 129.0, 127.0, 126.0, 125.0, 124.6, 122.2, 62.1, 55.9, 53.4 (d, *J*=15.1 Hz), 34.1, 13.9, 13.6.

HRMS (ESI) $C_{19}H_{21}FO_6S$ calcd 397.1116 [M+H], found 397.1115 [M+H].

Enantiomeric excess was determined by HPLC analysis using a Chiralpak® IC column (hexane : i-PrOH 90 : 10, flow rate 1.0 mL/min, λ = 219 nm): ent-(S) t_r = 7.87 min and ent-(R) t_r = 14.83 min.

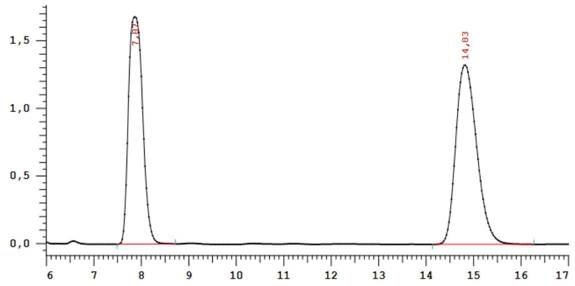

No.	RT	Area	Conc 1	BC
1	7,87	17454656	46,087	MC
2	14,83	20418358	53,913	MC
		37873014	100,000	

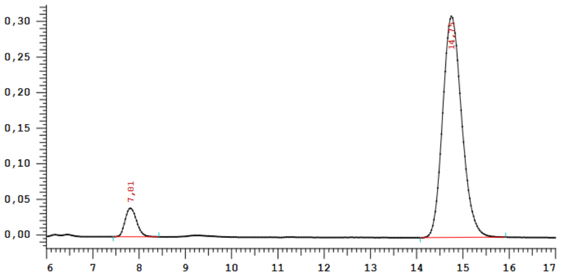

No.	RT	Area	Conc 1	BC
1	7,81	345310	7,162	MC
2	14,75	4476127	92,838	MC
		4821437	100,000	

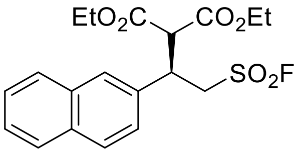

Under conditions of the general procedure conversion of sulfonyl fluoride **1r** was low (45%, **2r** was formed with 85% ee), and the reaction mixture remained inhomogeneous (due to a poor solubility of **1r**). Thus, product **2r** was not isolated.

<u>Alternative preparations of **2r**</u>:

According to general procedure, using THF as a solvent, from sulfonyl fluoride **1r** (118 mg, 0.50 mmol) to obtain product **2r** (164 mg, 0.41 mmol, 83%, 60% ee), as a white solid.

According to general procedure, but using more diluted toluene solution (homogeneous) and different proportion of components:

A 3 ml Teflon vial was loaded with catalyst **3d** (21 mg, 45 µmol, 10 mol%), sulfonyl fluoride **1r** (106 mg, 0.45 mmol), diethyl malonate (216 mg, 1.35 mmol, 3 equiv), and filled up with toluene (c_{1r} = 0.15 M). The teflon vial was then subjected to 9 kbar of hydrostatic pressure for 20h at rt. After decompression, reaction mixture was purified by column chromatography at silica gel (hexanes : ethyl acetate 100 : 0 to 85 : 15) to obtain product **2r** (160 mg, 0.40 mmol, 90%, 87% ee), as a white solid.

^{1}H NMR (400 MHz, $CDCl_3$) δ 7.86-7.75 (m, 4H), 7.52-7.46 (m, 2H), 7.38 (dd, *J*=8.5, 1.9 Hz, 1H), 4.28-4.17 (m, 4H), 4.10 (ddd, *J*=14.9, 10.5, 1.7 Hz, 1H), 4.01 (q, *J*=7.2 Hz, 2H), 3.87 (d, *J*=8.0 Hz, 1H), 1.23 (t, *J*=7.1 Hz, 3H), 1.03 (t, *J*=7.1 Hz, 3H).

^{19}F NMR (376 MHz, $CDCl_3$) δ 59.3 (dd, *J*=6.7, 1.9 Hz).

^{13}C NMR (100 MHz, $CDCl_3$) δ 167.4, 166.6, 133.9, 133.2, 133.0, 128.9, 127.9, 127.7, 127.7, 126.5, 126.5, 125.1, 62.2, 62.0, 56.5 (d, *J*=1.5 Hz), 53.8 (d, *J*=14.9 Hz), 40.7, 13.9, 13.7.

HRMS (ESI) $C_{19}H_{21}FO_6S$ calcd 397.1116 [M+H], found 397.1116 [M+H].

Enantiomeric excess was determined by HPLC analysis using a Chiralpak® IC column (hexane : i-PrOH 90 : 10, flow rate 1.0 mL/min, λ = 219 nm): ent-(S) t_r = 9.09 min and ent-(R) t_r = 11.23 min.

Racemic sample

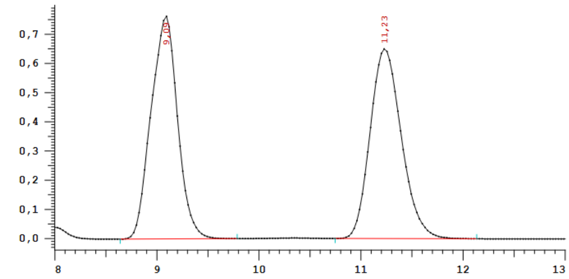

No.	RT	Area	Conc 1	BC
1	9,09	6888833	49,906	MC
2	11,23	6914892	50,094	MC
		13803725	100,000	

Reaction conducted in THF (0.5 M)

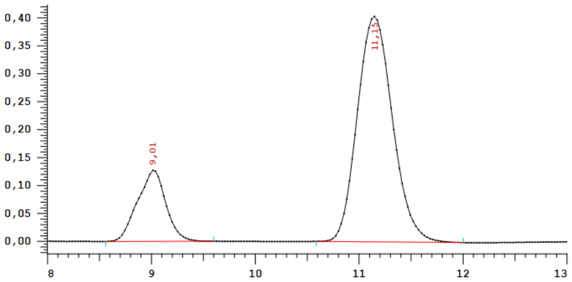

No.	RT	Area	Conc 1	BC
1	9,01	1200541	20,145	MC
2	11,15	4758885	79,855	MC
		5959426	100,000	

Reaction conducted in toluene (0.15 M)

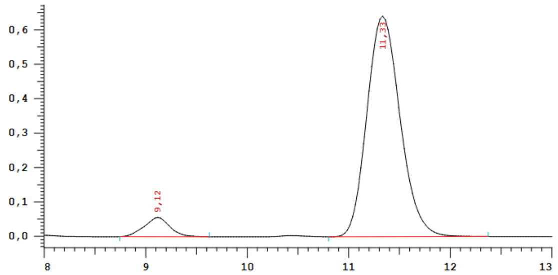

No.	RT	Area	Conc 1	BC
1	9,12	485648	6,258	MC
2	11,33	7275112	93,742	MC
		7760760	100,000	

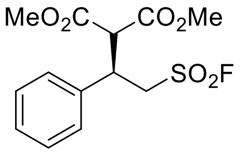

Product **4** (316 mg, 0.99 mmol, 95%, 92% ee) was prepared according to the general procedure from sulfonyl fluoride **1a** (195 mg, 1.05 mmol) and dimethyl malonate, and isolated using column chromatography on silica gel (hexanes : ethyl acetate 100 : 0 to 85 : 15), as an yellow oil.

^{1}H NMR (400 MHz, $CDCl_3$) δ 7.39-7.24 (m, 5H), 4.14 (ddd, *J*=13.9, 6.6, 2.6 Hz, 1H), 4.09-3.94 (m, 2H), 3.82 (d, *J*=7.7 Hz, 1H), 3.75 (s, 3H), 3.58 (s, 3H).

^{19}F NMR (376 MHz, $CDCl_3$) δ 59.2 (dd, *J*=6.0, 1.0 Hz).

^{13}C NMR (100 MHz, $CDCl_3$) δ 167.8, 167.1, 136.5, 129.0, 128.6, 127.9, 56.2 (d, *J*=1.6 Hz), 53.7 (d, *J*=14.9 Hz), 53.0, 52.9, 40.6.

HRMS (ESI) $C_{13}H_{15}FO_6S$ calcd 319.0646 [M+H], found 319.0646 [M+H].

Enantiomeric excess was determined by HPLC analysis using a Chiralpak® IC column (hexane : i-PrOH 90 : 10, flow rate 1.0 mL/min, λ = 219 nm): ent-(S) t_r = 9.31 min and ent-(R) t_r = 13.15 min.

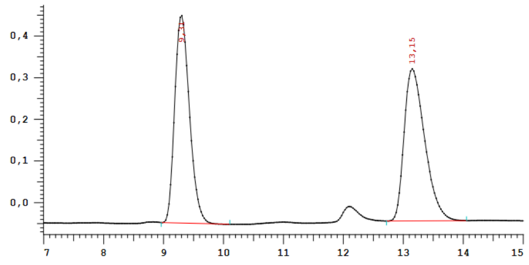

No.	RT	Area	Conc 1	BC
1	9,31	4253568	49,412	MC
2	13,15	4354812	50,588	MC

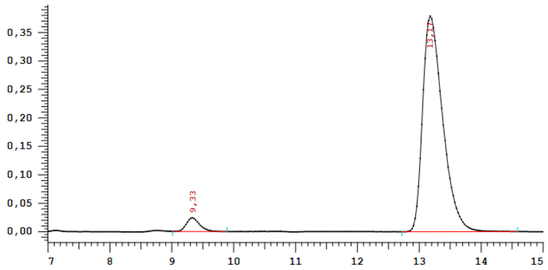

No.	RT	Area	Conc 1	BC
1	9,33	177505	3,991	MC
2	13,17	4269584	96,009	MC
		4447089	100,000	

Products of further transformations of adduct 2a

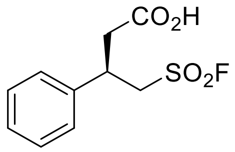

Compound **5a**

In a 10 ml screw-capped test tube sulfonyl fluoride **2a** (130 mg, 0.376 mmol) was dissolved in 6 M HCl_{aq} : AcOH mixture (1 : 1, 4 ml) and heated at 100 °C overnight. After cooling to rt, reaction mixture was extracted with ethyl acetate, organic phase was dried with Na_2SO_4, evaporated and purified using column chromatography on silica gel (hexanes : ethyl acetate 90 : 10 to 50 : 50) to obtain sulfonyl fluoride **5a** (52 mg, 0.21 mmol, 56%), as a white solid.

^{1}H NMR (400 MHz, DMSO-d_6) δ 12.32 (s, 1H), 7.39-7.30 (m, 4H), 7.28-7.23 (m, 1H), 4.35 (dd, *J*=7.3, 4.7 Hz, 2H), 3.61 (dq, *J*=9.2, 6.8 Hz, 1H), 2.84 (dd, *J*=16.3, 5.7 Hz, 1H), 2.65 (dd, *J*=16.2, 9.0 Hz, 1H).

^{19}F NMR (376 MHz, DMSO-d_6) δ 59.2 (t, *J*=4.9 Hz).

^{13}C NMR (100 MHz, DMSO-d_6) δ 172.2, 140.5, 128.9, 128.2, 127.8, 55.3 (d, *J*=11.8 Hz), 40.1 (d, *J*=2.0 Hz), 37.8.

HRMS (ESI) $C_{10}H_{11}FO_4S$ calcd 245.0278 [M-H], found 245.0285 [M-H].

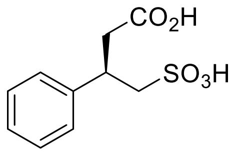

Compound **5b**

Aqueous layer from the above procedure was evaporated, to obtain sulfonic acid **5b** (40 mg, 0.164 mmol, 43%), as a white solid.

^{1}H NMR (400 MHz, DMSO-d_6) δ 7.29-7.12 (m, 5H), 3.46 (tt, *J*=10.7, 3.5 Hz, 1H), 3.35 (dd, *J*=16.1, 3.8 Hz, 1H), 2.85 (dd, *J*=13.6, 10.4 Hz, 1H), 2.57 (dd, *J*=13.6, 3.2 Hz, 1H), 2.49-2.44 (m, 1H).

^{13}C NMR (100 MHz, DMSO-d_6) δ 173.7, 145.1, 128.6, 127.8, 126.6, 57.3, 39.8, 38.8.

HRMS (ESI) $C_{10}H_{12}O_5S$ calcd 243.0322 [M-H], found 243.0329 [M-H].

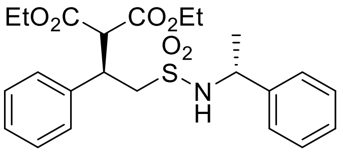

Compound **6a**

To a 10 ml screw-capped test tube sulfonyl fluoride **2a** (70 mg, 0.202 mmol), (*R*)-1-phenylethylamine (42 mg, 0.35 mmol, 1.7 equiv), DIPEA (77 mg, 0.60 mmol, 3 equiv) and acetonitrile (300 μl) were added, and the reaction mixture was heated at 80 °C for 2 d. After cooling to rt, acetonitrile was evaporated, residue was suspended in toluene and purified by column chromatography on silica gel (hexanes : ethyl acetate gradient 100 : 0 to 60 : 40) to obtain product **6a** (88 mg, 0.197 mmol, 97%, ca. 94 : 6 dr), as a white solid.

The product was formed, as a mixture of diastereoisomers. The diastereoisomeric ratio corresponded to enantiomeric excess of the starting material (**2a**). Only NMR resonances of major diastereoisomer are listed below.

^{1}H NMR (400 MHz, $CDCl_3$) δ 7.35-7.27 (m, 3H), 7.21-7.16 (m, 5H), 7.04-7.01 (m, 2H), 4.51 (p, *J*=7.0 Hz, 1H), 4.33 (d, *J*=7.2 Hz, 1H), 4.19 (q, *J*=7.1 Hz, 2H), 3.90-3.81 (m, 3H), 3.74 (d, *J*=9.1 Hz, 1H), 3.42 (dd, *J*=14.5, 5.9 Hz, 1H), 3.04 (dd, *J*=14.5, 7.9 Hz, 1H), 1.43 (d, *J*=6.9 Hz, 3H), 1.24 (t, *J*=7.1 Hz, 3H), 0.95 (t, *J*=7.1 Hz, 3H).

^{13}C NMR (100 MHz, $CDCl_3$) δ 168.1, 167.1, 142.3, 138.1, 128.9, 128.5, 128.4, 127.9, 127.7, 126.3, 62.0, 61.5, 57.0, 56.6, 53.8, 40.6, 24.0, 14.0, 13.7.

HRMS (ESI) $C_{23}H_{29}NO_6S$ calcd 448.1788 [M+H], found 448.1787 [M+H].

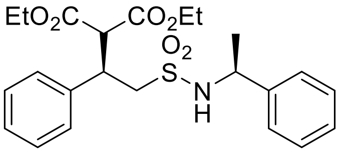

Compound **6b**

To a 10 ml screw-capped test tube sulfonyl fluoride **2a** (90 mg, 0.260 mmol), (*S*)-1-phenylethylamine (34 mg, 0.28 mmol, 1.1 equiv), DIPEA (67 mg, 0.52 mmol, 2 equiv) and acetonitrile (300 μl) were added, and the reaction mixture was heated at 80 °C for 2 d. After cooling to rt, acetonitrile was evaporated, residue was suspended in toluene and purified by column chromatography on silica gel (hexanes : ethyl acetate gradient 100 : 0 to 60 : 40) to obtain product **6b** (105 mg, 0.235 mmol, 90%, ca. 94 : 6 dr), as a colourless oil.

The product was formed, as a mixture of diastereoisomers. The diastereoisomeric ratio corresponded to enantiomeric excess of the starting material (**2a**). Only NMR resonances of major diastereoisomer are listed below.

^{1}H NMR (400 MHz, $CDCl_3$) δ 7.34-7.22 (m, 8H), 7.19-7.15 (m, 2H), 4.45 (p, *J*=6.9 Hz, 1H), 4.20 (q, *J*=7.2 Hz, 1H), 4.19 (q, *J*=7.1 Hz, 1H), 4.11 (d, *J*=6.5 Hz, 1H), 3.99 (td, *J*=9.1, 4.3 Hz, 1H), 3.94 (q, *J*=7.1 Hz, 2H), 3.78 (d, *J*=9.1 Hz, 1H), 3.52 (dd, *J*=14.4, 4.2 Hz, 1H), 3.36 (dd, *J*=14.4, 9.1 Hz, 1H), 1.27 (d, *J*=6.9 Hz, 3H), 1.23 (t, *J*=7.1 Hz, 3H), 1.01 (t, *J*=7.1 Hz, 3H).

^{13}C NMR (100 MHz, $CDCl_3$) δ 167.7, 167.0, 142.6, 138.5, 128.8, 128.7, 128.6, 127.9, 127.7, 126.1, 62.0, 61.6, 57.0, 56.8, 53.5, 40.9, 23.3, 14.0, 13.7.

HRMS (ESI) $C_{23}H_{29}NO_6S$ calcd 448.1788 [M+H], found 448.1786 [M+H].

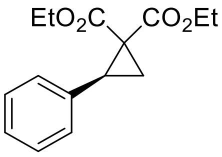

Compound **7**

A 30 ml schlenk flask, equipped with stirring bar, was charged with sulfonyl fluoride **2a** (453 mg, 1.31 mmol, 89% ee) and argonated. Then, dry THF (6.5 ml) and DBU (400 mg, 2.63 mmol, 2 equiv) were added, and the mixture was stirred in oil bath at 55 °C for 18 h. The mixture was evaporated and purified by column chromatography on silica gel (cyclohexane : AcOEt 85 : 15) to obtain diethyl 2-phenylcyclopropane-1,1-dicarboxylate (**7**, 284 mg, 1.08 mmol, 83%, 89% ee), as a pale yellowish liquid.

Reaction at racemic substrate was carried out accordingly.

^{1}H NMR (400 MHz, $CDCl_3$) δ 7.29-7.17 (m, 5H), 4.24 (qq, *J*=10.8, 7.1 Hz, 2H), 3.84 (q, *J*=7.0 Hz, 2H), 3.21 (t, *J*=8.6 Hz, 1H), 2.17 (dd, *J*=8.0, 5.2 Hz, 1H), 1.70 (dd, *J*=9.2, 5.2 Hz, 1H), 1.29 (t, *J*=7.1 Hz, 3H), 0.86 (t, *J*=7.1 Hz, 3H).

^{13}C NMR (100 MHz, $CDCl_3$) δ 169.9, 166.6, 134.6, 128.5, 128.1, 127.3, 61.7, 61.1, 37.4, 32.1, 18.7, 14.1, 13.6.

$[\alpha]_D^{25}$ = –113.5 (c=1.08, $CHCl_3$, 89% ee) (lit. $[\alpha]_D^{20}$ = –45 (c=1.13 , $CHCl_3$, 34% ee), D. Marcoux, S. R. Goudreau, A. B. Charette. *J. Org. Chem.* **2009**, *74*, 8939-8955.

Enantiomeric excess was determined by HPLC analysis using a Chiralpak® IC column (hexane : i-PrOH 95 : 5, flow rate 1.0 mL/min, λ = 219 nm): ent-(R) t_r = 9.52 min and ent-(S) t_r = 11.07 min.

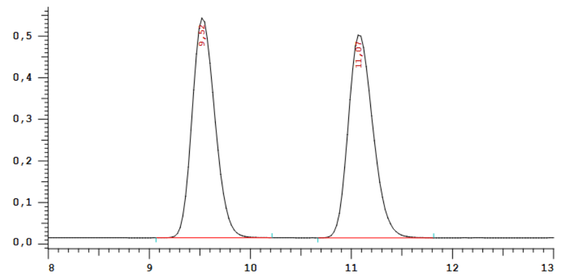

No.	RT	Area	Conc 1	BC
1	9,52	4128014	49,901	MC
2	11,07	4144312	50,099	MC
		8272326	100,000	

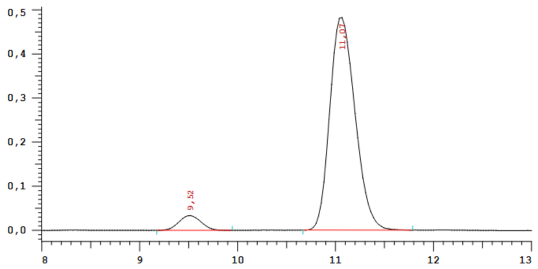

No.	RT	Area	Conc 1	BC
1	9,52	258878	5,649	MC
2	11,07	4323580	94,351	MC
		4582458	100,000	

Single-crystal X-Ray diffraction analysis of 2g (CCDC 2252207)

Sample of adduct **2g** was dissolved in a hexane : DCM mixture (ca. 9 : 1) and left for slow evaporation at rt. Good quality single-crystal was selected for the X-ray diffraction experiment at *T* = 100(2) K. Diffraction data were collected on the Agilent Technologies SuperNova Dual Source diffractometer with Cu*K*α radiation (λ = 1.54184 Å) using CrysAlis Pro software [CrysAlis CCD and CrysAlis RED, Oxford Diffraction, Oxford Diffraction Ltd: Yarnton, 2008]. The analytical absorption correction using a multifaceted crystal model based on expressions derived by R. C. Clark & J. S. Reid was applied [R. C. Clark, J. S. Reid. *Acta Cryst. Sect. A* **1994**, *51*, 887-897]. The structural determination procedure was carried out using the SHELX package [G. M. Sheldrick, *Acta Cryst. Sect. A* **2015**, *71*, 3-8]. The structures were solved with intrinsic phasing method and then successive least-square refinement was carried out based on the full-matrix least-squares method on F^2 using the SHELXL program [G. M. Sheldrick, *Acta Cryst. Sect. A* **2015**, *71*, 3-8]. All H-atoms were positioned geometrically with C–H bond length equal to 0.93, 0.96, 0.97 and 0.98 Å for the aromatic, methyl, methylene and methine H-atoms, respectively, and constrained to ride on their parent atoms with U_{iso}(H) = xU_{eq}(C), were x = 1.5 for the methyl H-atoms, and 1.2 for the aromatic, methylene and methine H-atoms, respectively. The figure below was prepared using CCDC Mercury 3.8 (C. F. Macrae, P. R. Edgington, P. McCabe, E. Pidcock, G. P. Shields, R. Taylor, M. Towler, J. Van De Streek. Mercury: Visualization and Analysis of Crystal Structures. *J. Appl. Crystallogr.* **2006**, *39*, 453-457).

Table 1. Crystal data and structure refinement for **2g**.

Identification code	**2g**
Empirical formula	$C_{15}H_{18}ClFO_6S$
Formula weight	380.80
Temperature/K	100(2)
Crystal system	monoclinic
Space group	$P2_1$
a/Å	10.1525(2)
b/Å	5.72515(15)
c/Å	15.0227(4)
α/°	90
β/°	98.389(2)
γ/°	90
Volume/Å^3	863.84(4)
Z	2
ρ_{calc}g/cm^3	1.464
μ/mm^{-1}	3.448
F(000)	396.0
Crystal size/mm^3	0.61 × 0.11 × 0.05
Radiation	CuKα (λ = 1.54184)
2Θ range for data collection/°	8.804 to 134.098
Index ranges	$-12 \le h \le 9$, $-6 \le k \le 6$, $-16 \le l \le 17$
Reflections collected	6097
Independent reflections	3039 [R_{int} = 0.0421, R_{sigma} = 0.0562]
Data/restraints/parameters	3039/1/219
Goodness-of-fit on F^2	1.063

Final R indexes [I>=2σ (I)]	R_1 = 0.0581, wR_2 = 0.1528
Final R indexes [all data]	R_1 = 0.0609, wR_2 = 0.1562
Largest diff. peak/hole / e Å^{-3}	0.60/-0.41
Flack parameter	0.00(3)

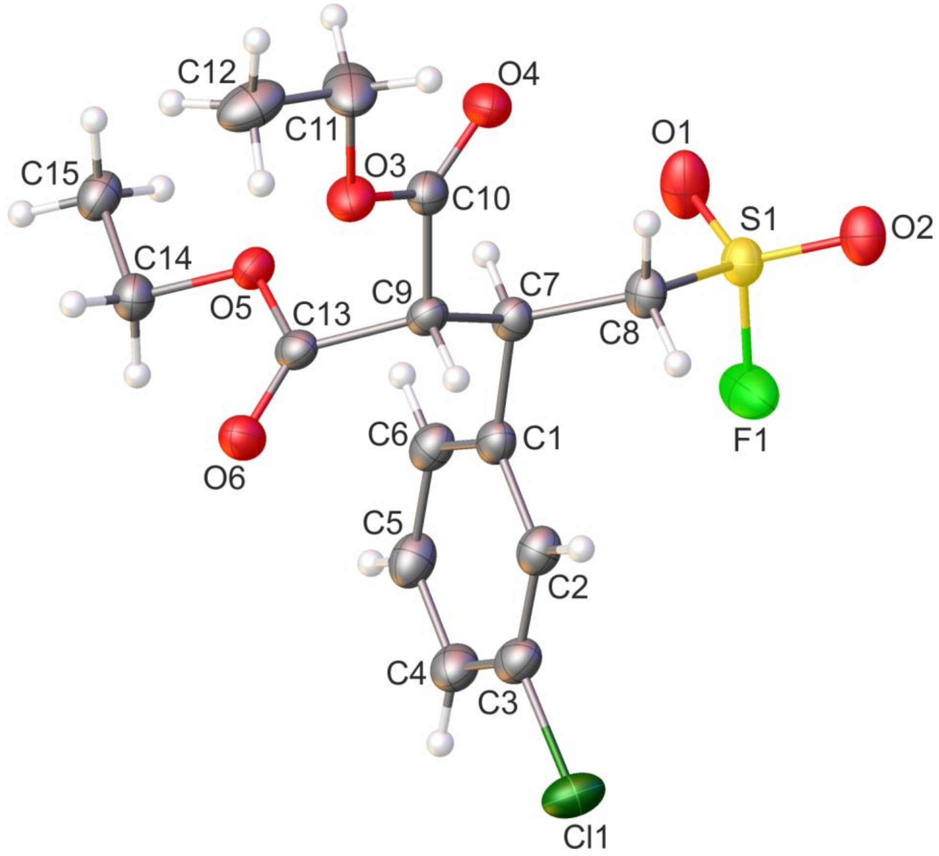

Figure 1. X-ray structure of compound *(R)*-**2g** with crystallographic atom numbering. Thermal ellipsoids were drawn at 50% probability level.

Reproductions of ^{1}H, ^{13}C, and ^{19}F NMR spectra

^{1}H NMR (400 MHz, $CDCl_3$)

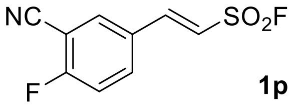

1p

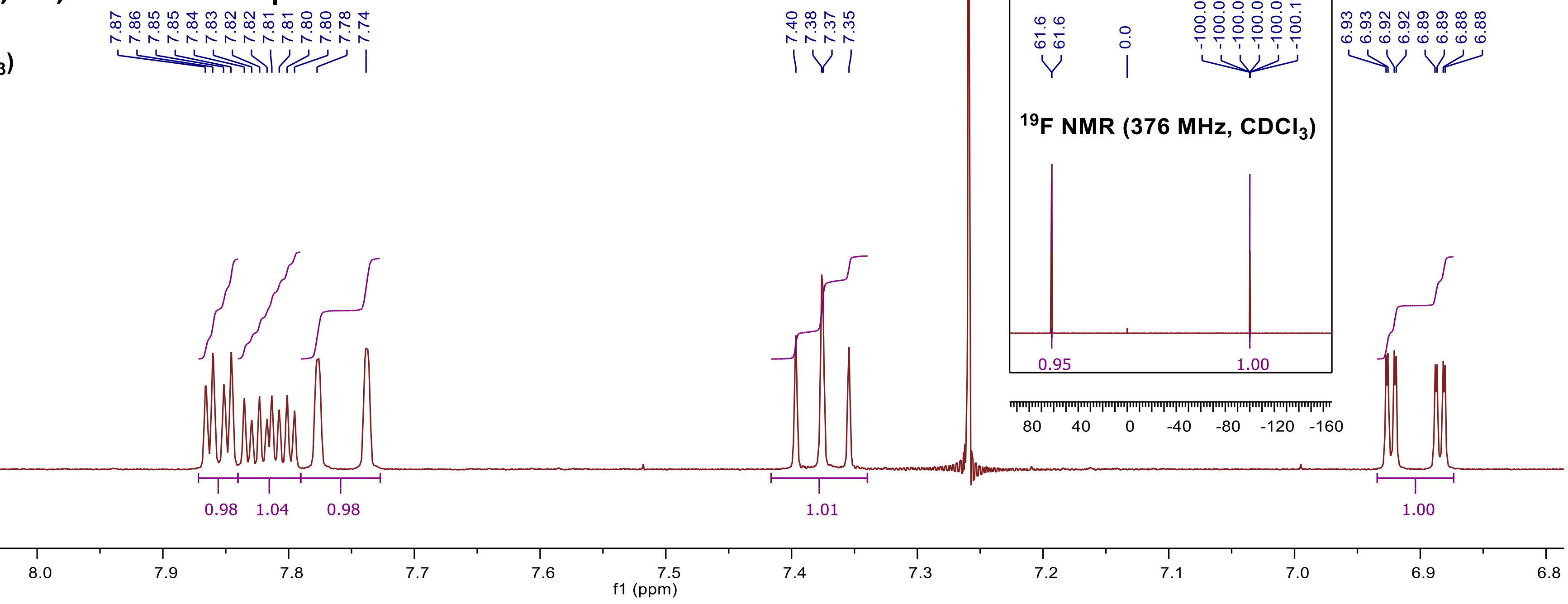

^{13}C NMR (100 MHz, $CDCl_3$)

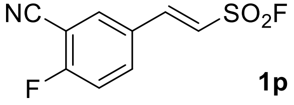

1p

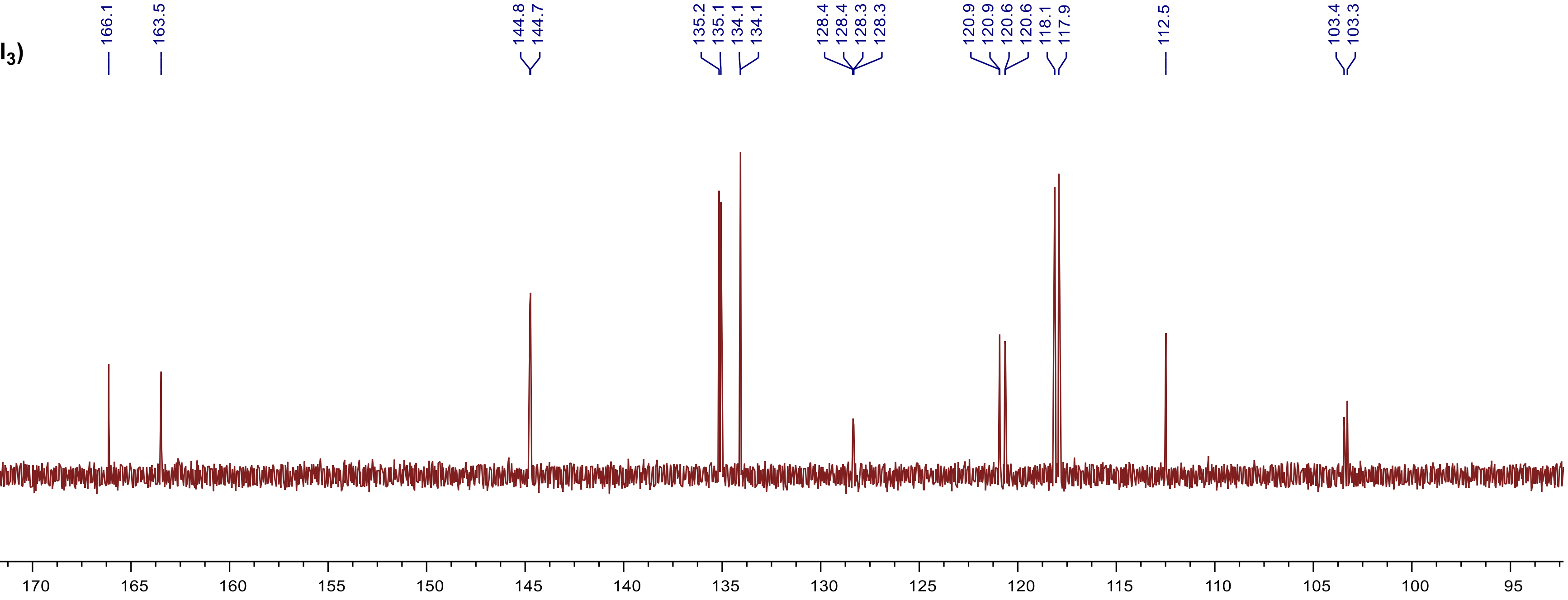

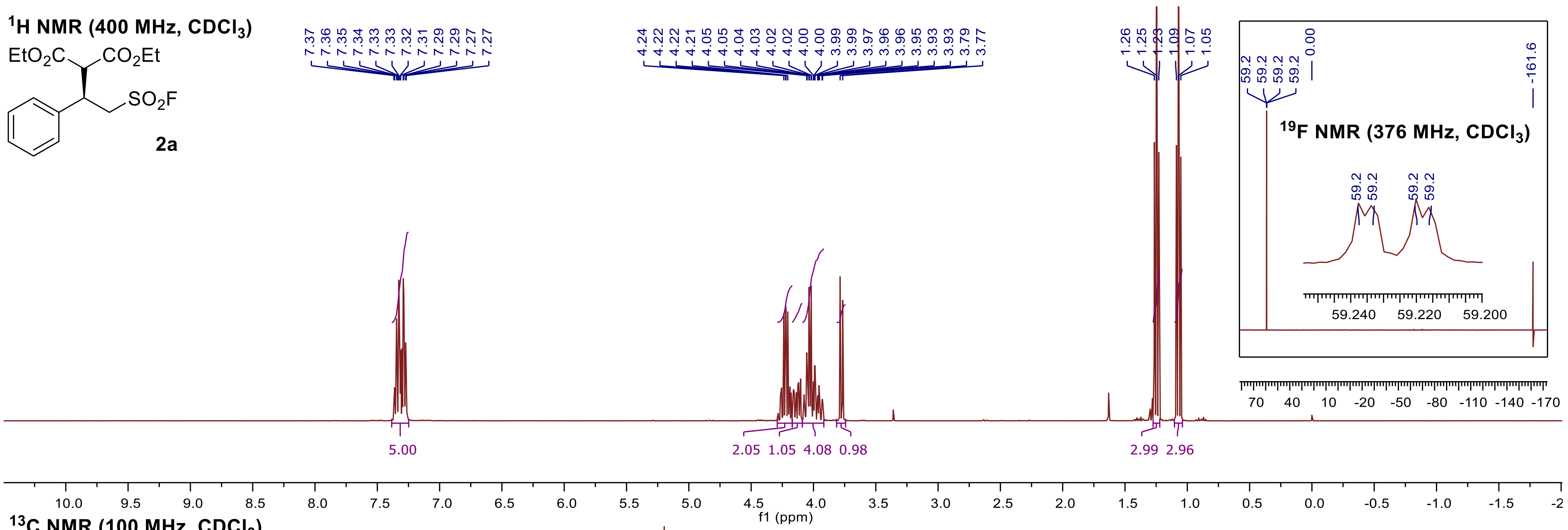

1H NMR (400 MHz, CDCl3)
EtO2C CO2Et
SO2F
2a
7.37 7.36 7.35 7.34 7.33 7.33 7.32 7.31 7.29 7.29 7.27 7.27
4.24 4.22 4.22 4.21 4.05 4.05 4.04 4.03 4.02 4.02 4.00 4.00 3.99 3.97 3.96 3.96 3.95 3.93 3.93 3.79 3.77
1.26 1.25 1.23 1.09 1.07 1.05
5.00
2.05 1.05 4.08 0.98
2.99 2.96
f1 (ppm)
19F NMR (376 MHz, CDCl3)
59.2 59.2 59.2 59.2
0.00
-161.6
59.240 59.220 59.200

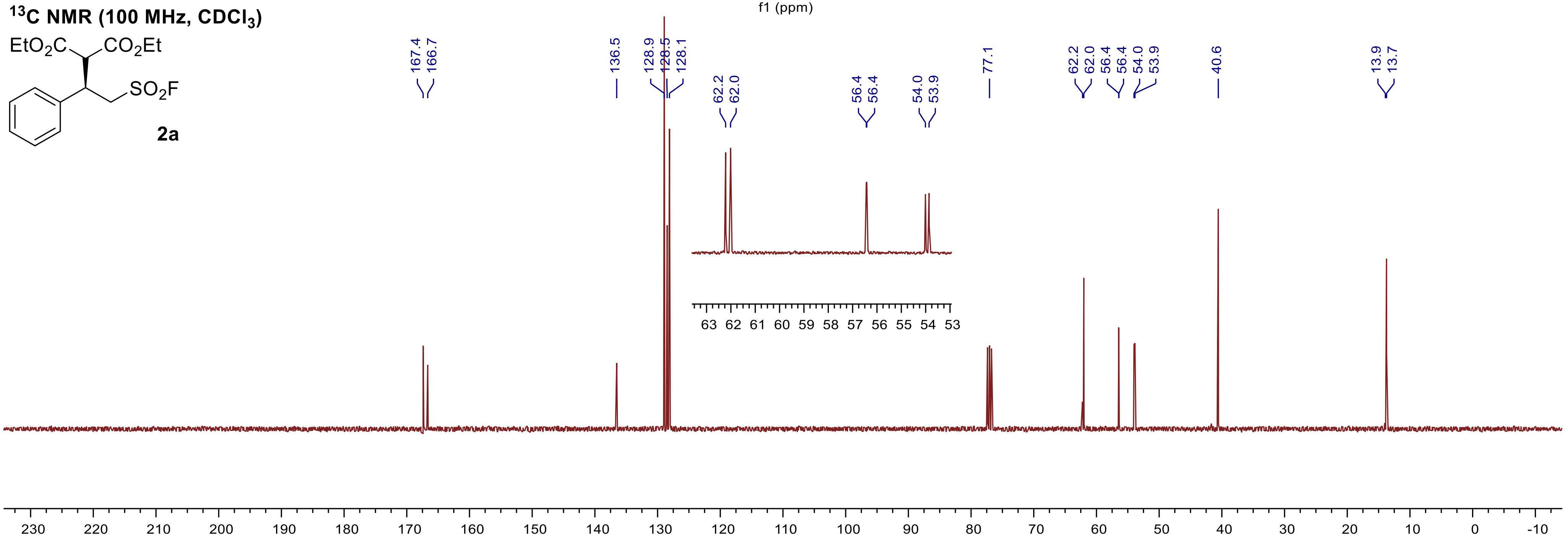

13C NMR (100 MHz, CDCl3)
EtO2C CO2Et
SO2F
2a
167.4 166.7
136.5
128.9 128.5 128.1
62.2 62.0
56.4 56.4
54.0 53.9
77.1
62.2 62.0 56.4 56.4 54.0 53.9
40.6
13.9 13.7
63 62 61 60 59 58 57 56 55 54 53

^{1}H NMR (400 MHz, $CDCl_3$)

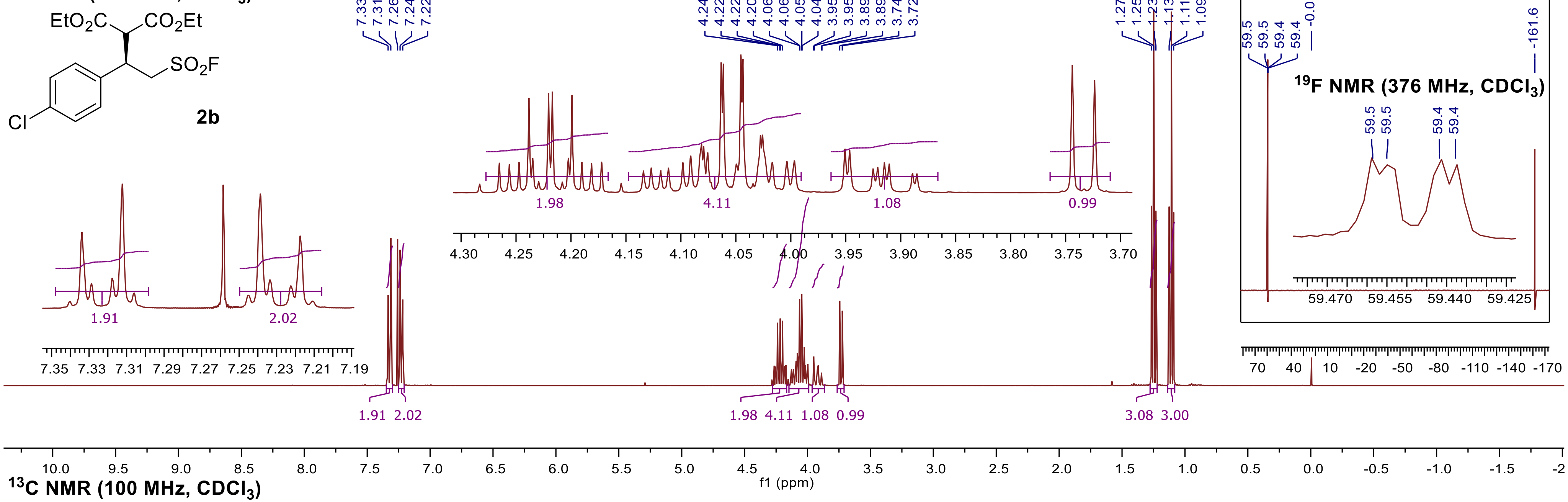

^{13}C NMR (100 MHz, $CDCl_3$)

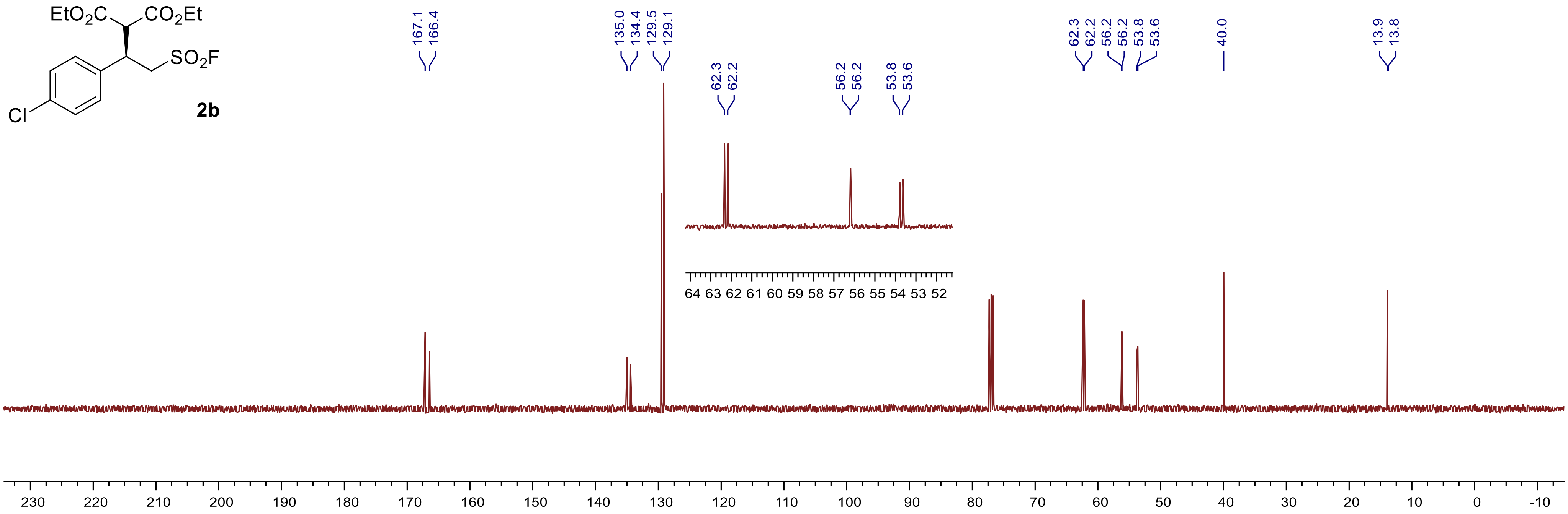

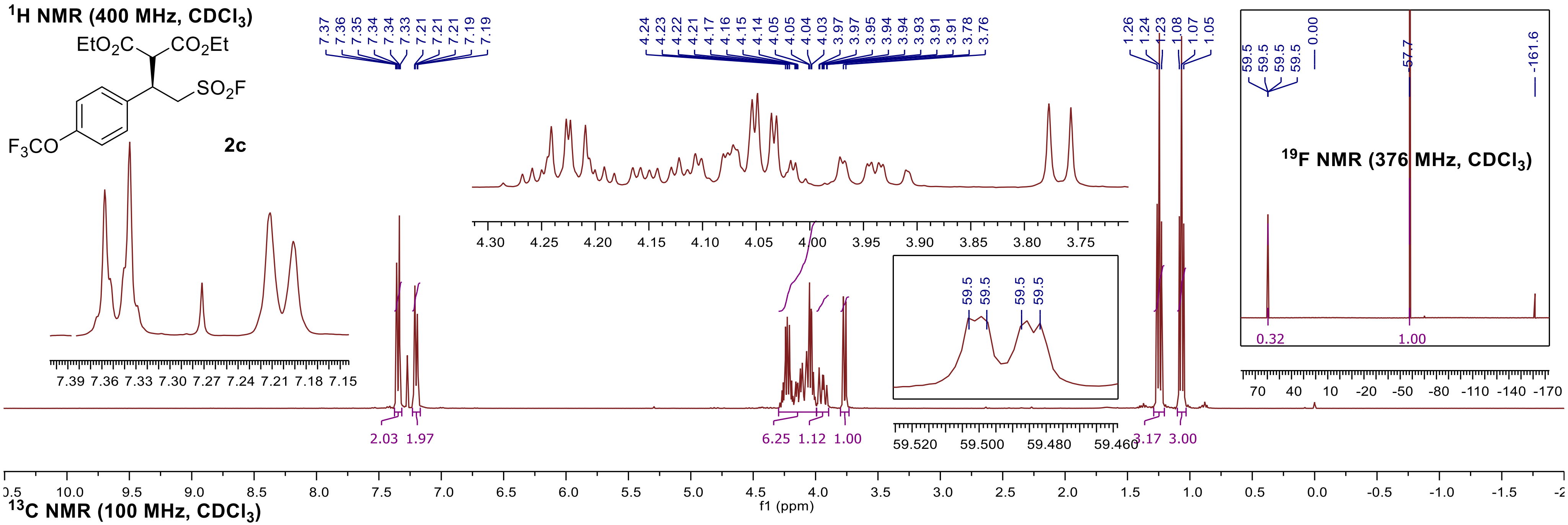

^{1}H NMR (400 MHz, CDCl$_3$)
2c
^{19}F NMR (376 MHz, CDCl$_3$)
f1 (ppm)

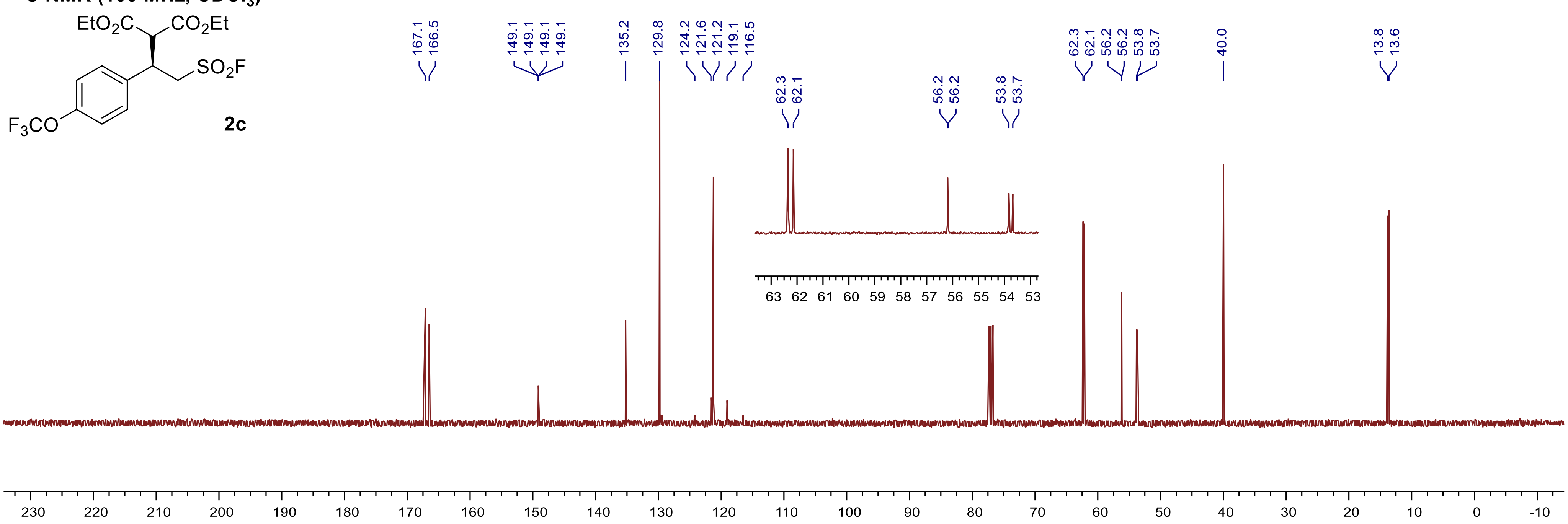

^{13}C NMR (100 MHz, CDCl$_3$)
2c

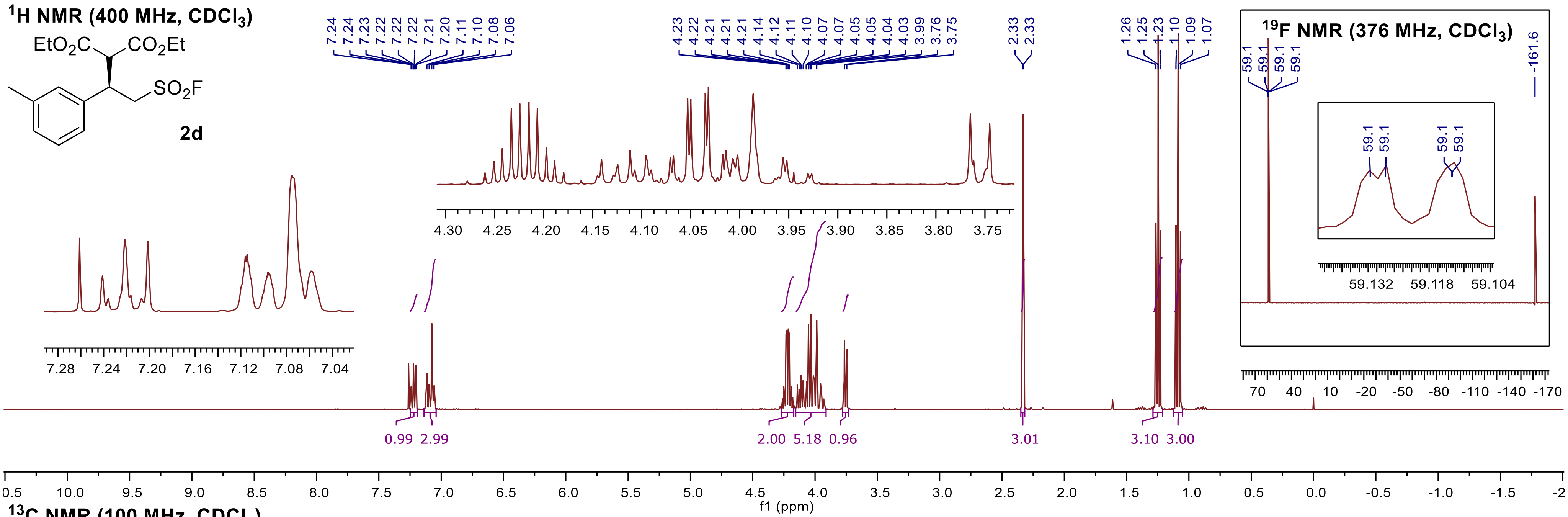
^{1}H NMR (400 MHz, $CDCl_3$)
EtO_2C
CO_2Et
SO_2F
2d
^{19}F NMR (376 MHz, $CDCl_3$)
59.1
-161.6
0.99 2.99
2.00 5.18 0.96
3.01
3.10 3.00
f1 (ppm)

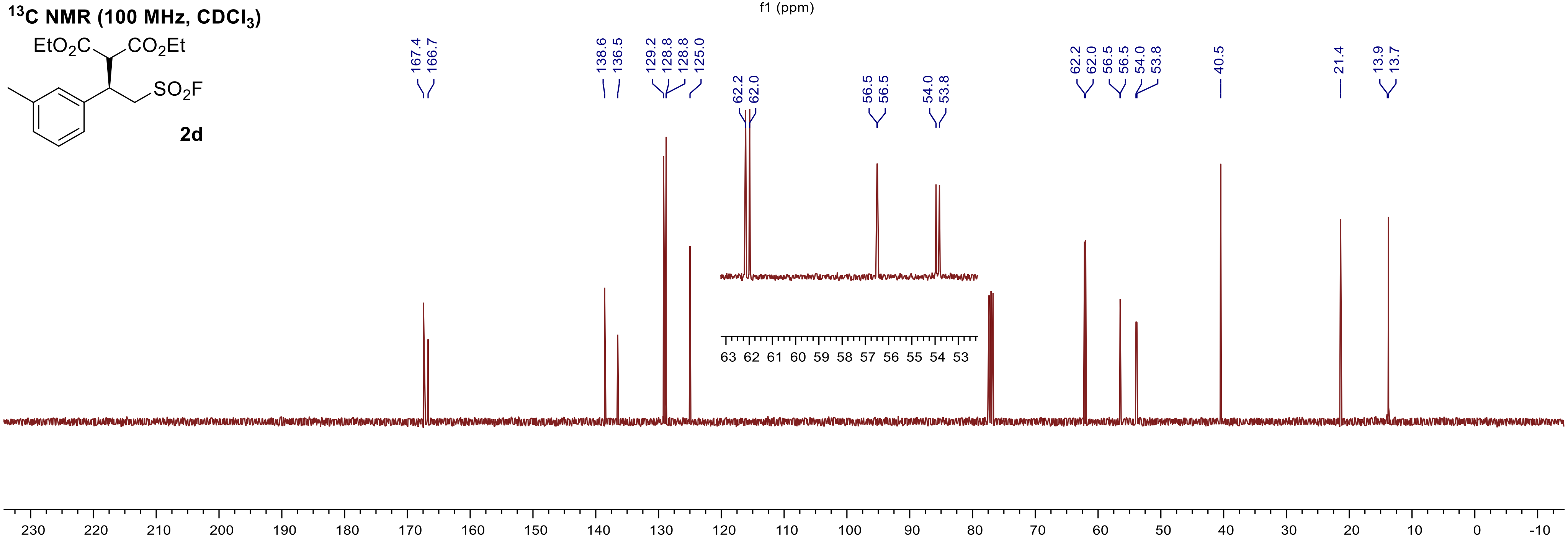
^{13}C NMR (100 MHz, $CDCl_3$)
EtO_2C
CO_2Et
SO_2F
2d
167.4
166.7
138.6
136.5
129.2
128.8
128.8
125.0
62.2
62.0
56.5
56.5
54.0
53.8
40.5
21.4
13.9
13.7

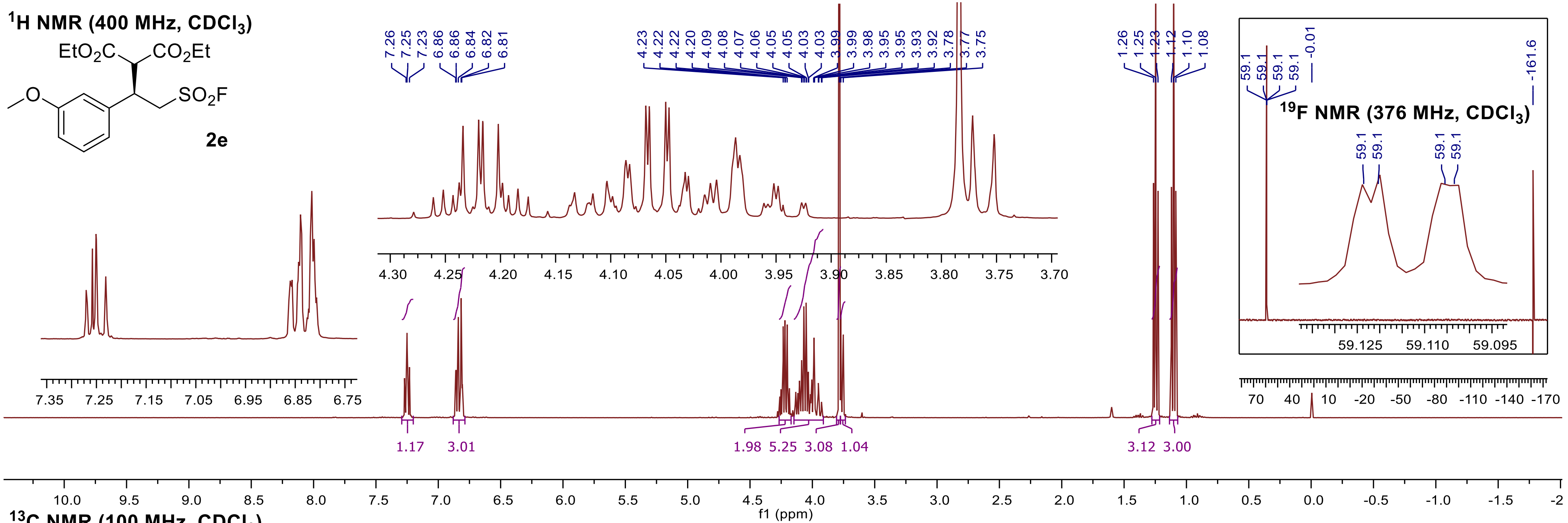

1H NMR (400 MHz, CDCl3)
2e
19F NMR (376 MHz, CDCl3)
f1 (ppm)

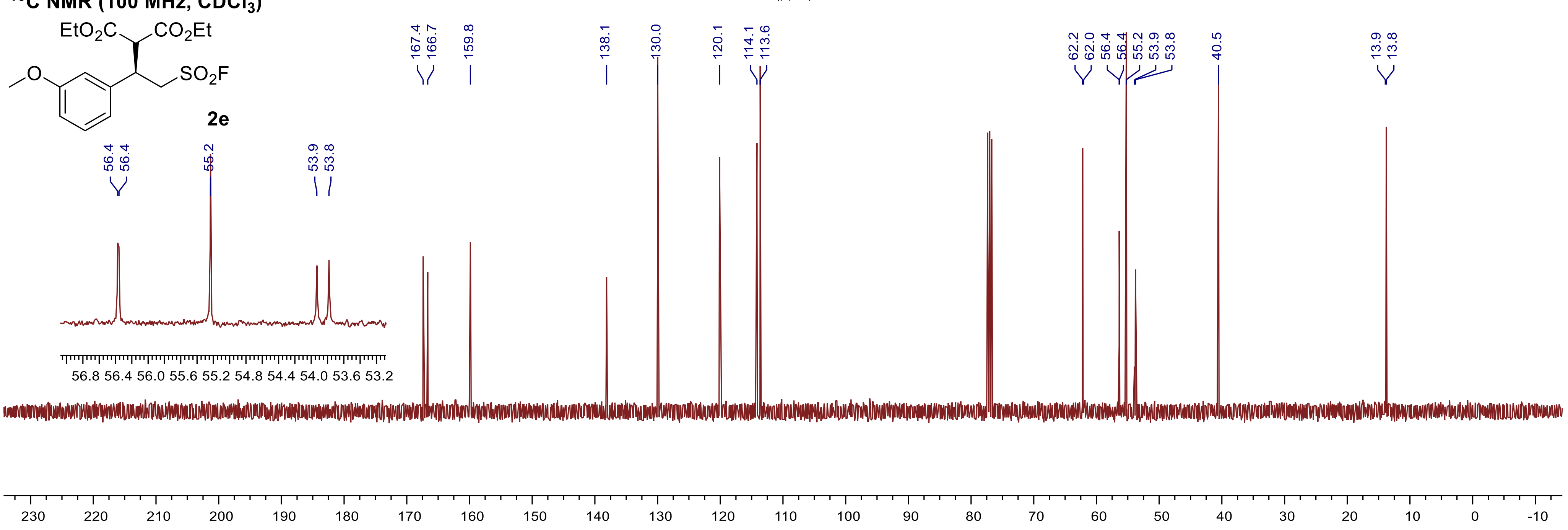

13C NMR (100 MHz, CDCl3)
2e

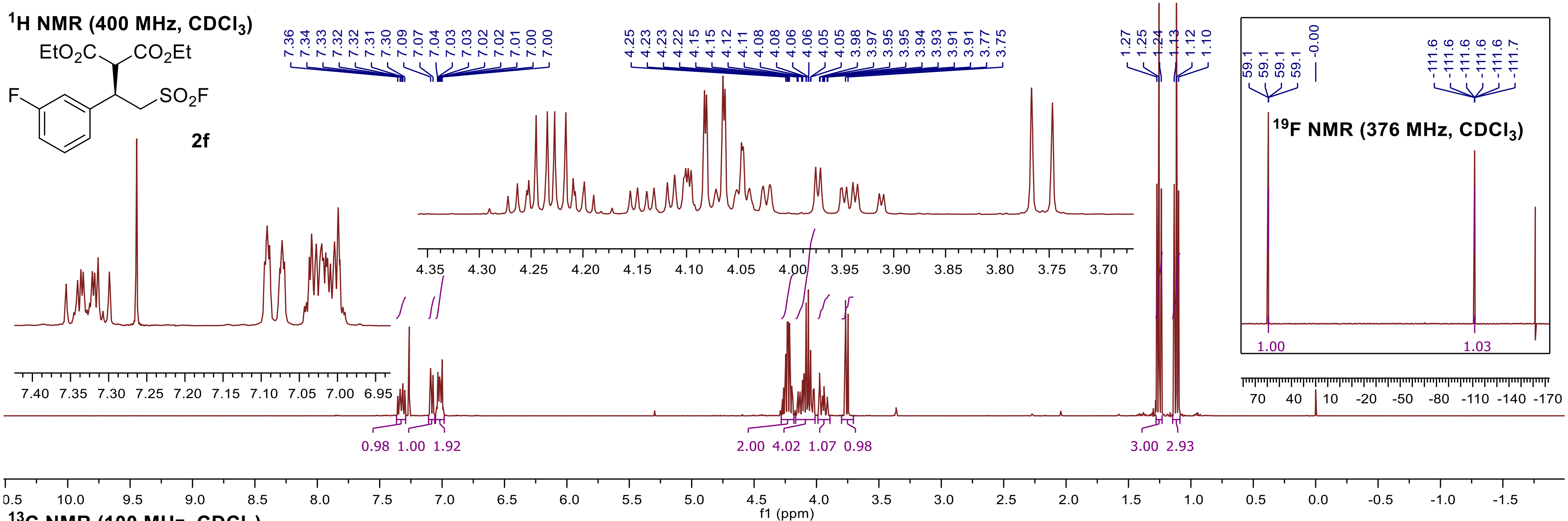
1H NMR (400 MHz, CDCl3)
EtO2C CO2Et
F
SO2F
2f
19F NMR (376 MHz, CDCl3)
1.00
1.03
0.98 1.00 1.92
2.00 4.02 1.07 0.98
3.00 2.93
f1 (ppm)

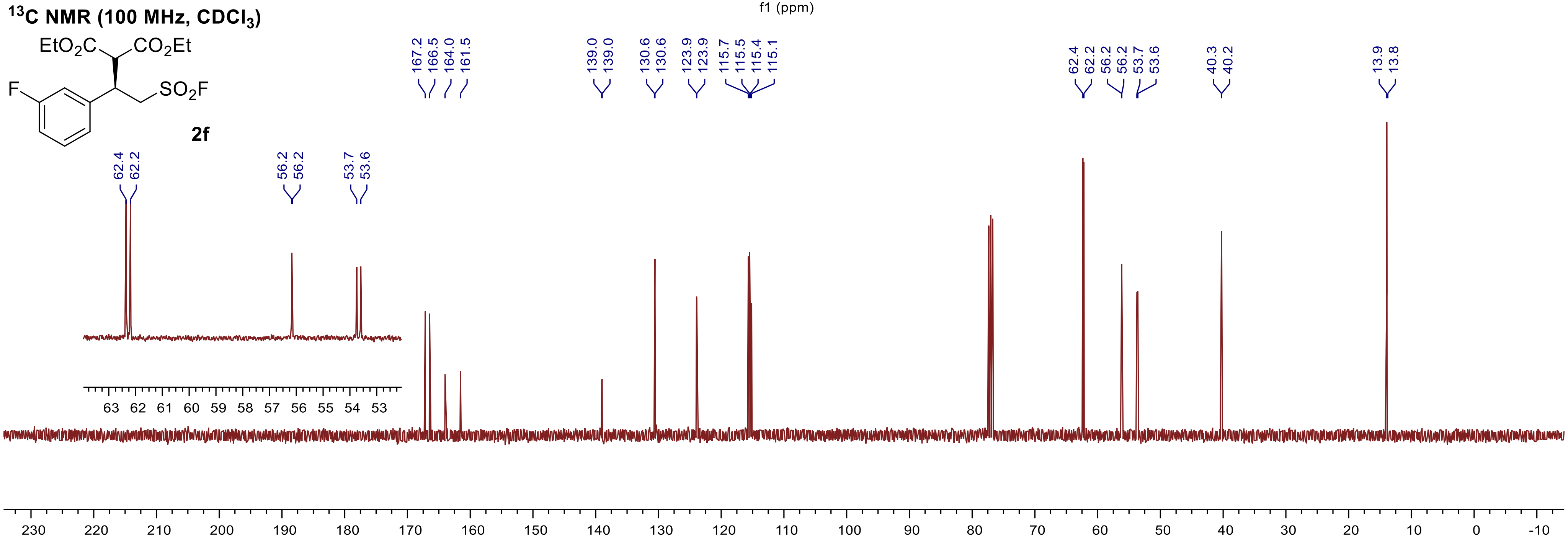
13C NMR (100 MHz, CDCl3)
EtO2C CO2Et
F
SO2F
2f

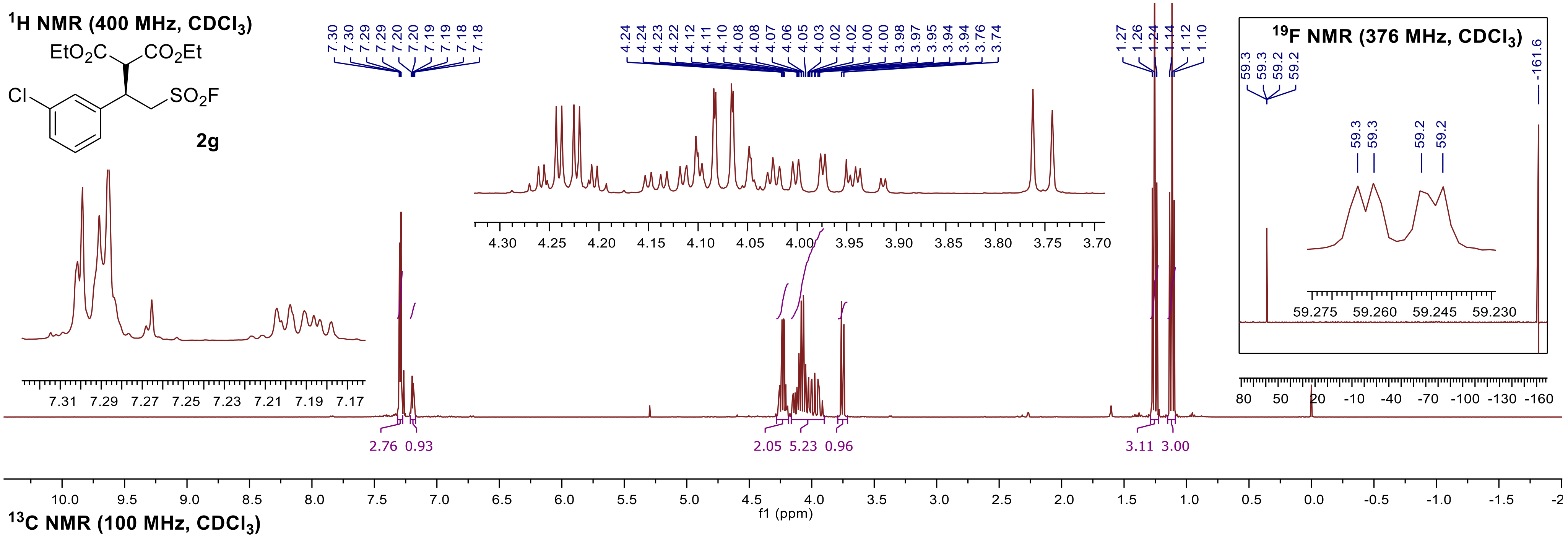

1H NMR (400 MHz, CDCl3)
EtO2C
CO2Et
Cl
SO2F
2g
19F NMR (376 MHz, CDCl3)
-161.6
59.3
59.3
59.2
59.2
2.76 0.93
2.05 5.23 0.96
3.11 3.00
f1 (ppm)

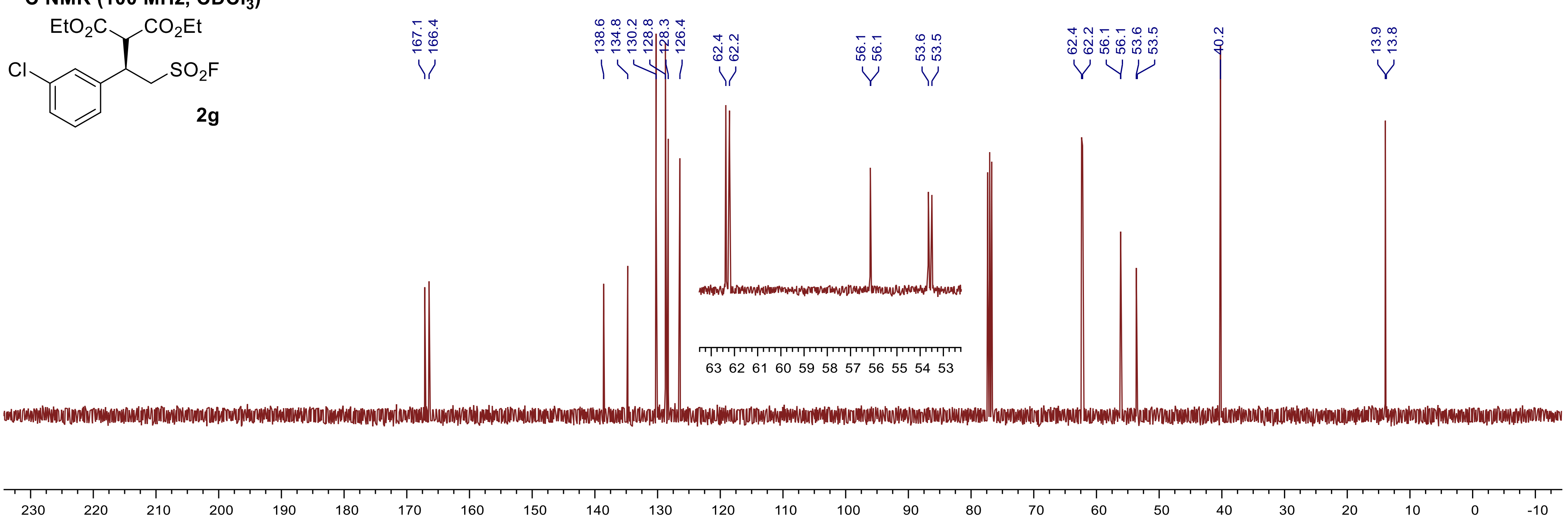

13C NMR (100 MHz, CDCl3)
EtO2C
CO2Et
Cl
SO2F
2g
167.1
166.4
138.6
134.8
130.2
128.8
128.3
126.4
62.4
62.2
56.1
56.1
53.6
53.5
40.2
13.9
13.8

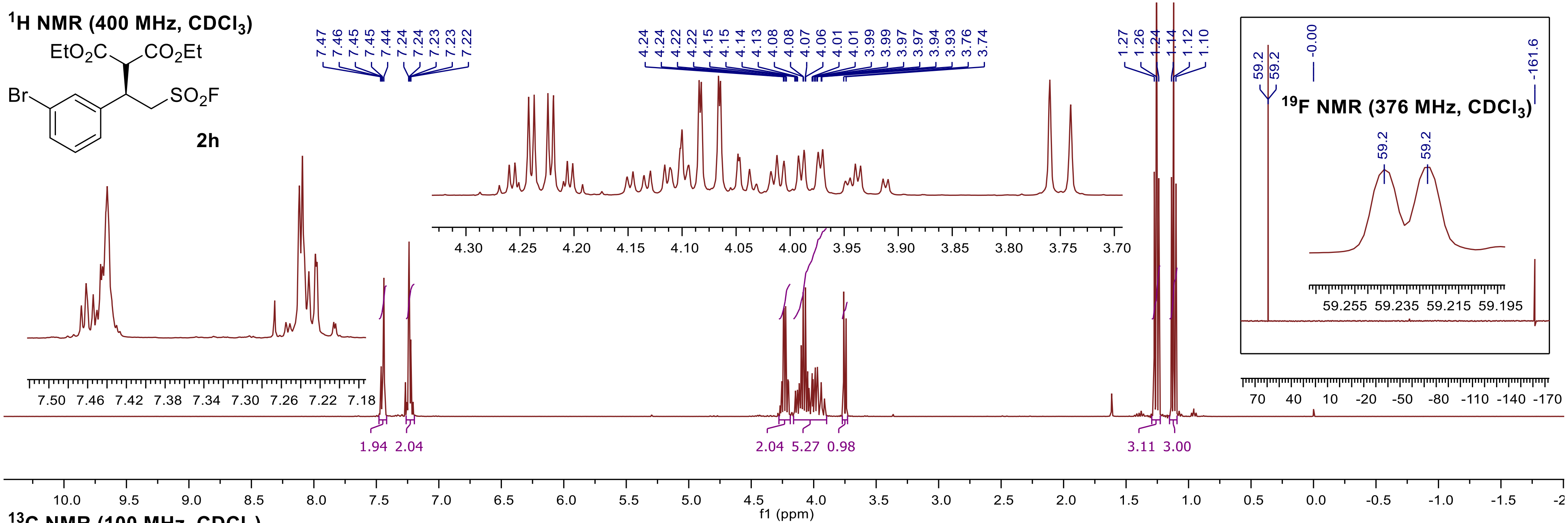
1H NMR (400 MHz, CDCl3)
EtO2C CO2Et
Br SO2F
2h
7.47 7.46 7.45 7.45 7.44 7.24 7.24 7.23 7.23 7.22
4.24 4.24 4.22 4.22 4.15 4.15 4.14 4.13 4.08 4.08 4.07 4.06 4.01 4.01 3.99 3.99 3.97 3.97 3.94 3.93 3.76 3.74
1.27 1.26 1.24 1.14 1.12 1.10
1.94 2.04
2.04 5.27 0.98
3.11 3.00
f1 (ppm)
19F NMR (376 MHz, CDCl3)
59.2 59.2
-0.00
-161.6

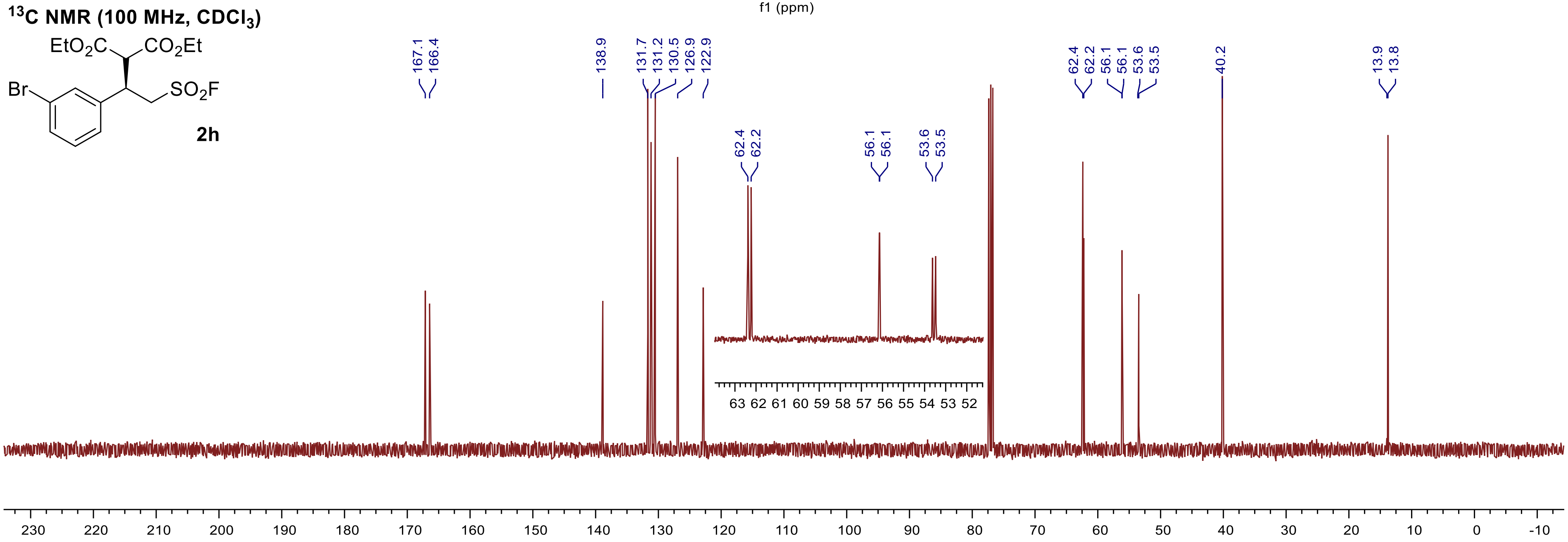
13C NMR (100 MHz, CDCl3)
EtO2C CO2Et
Br SO2F
2h
167.1 166.4
138.9
131.7 131.2 130.5 126.9 122.9
62.4 62.2 56.1 56.1 53.6 53.5
40.2
13.9 13.8

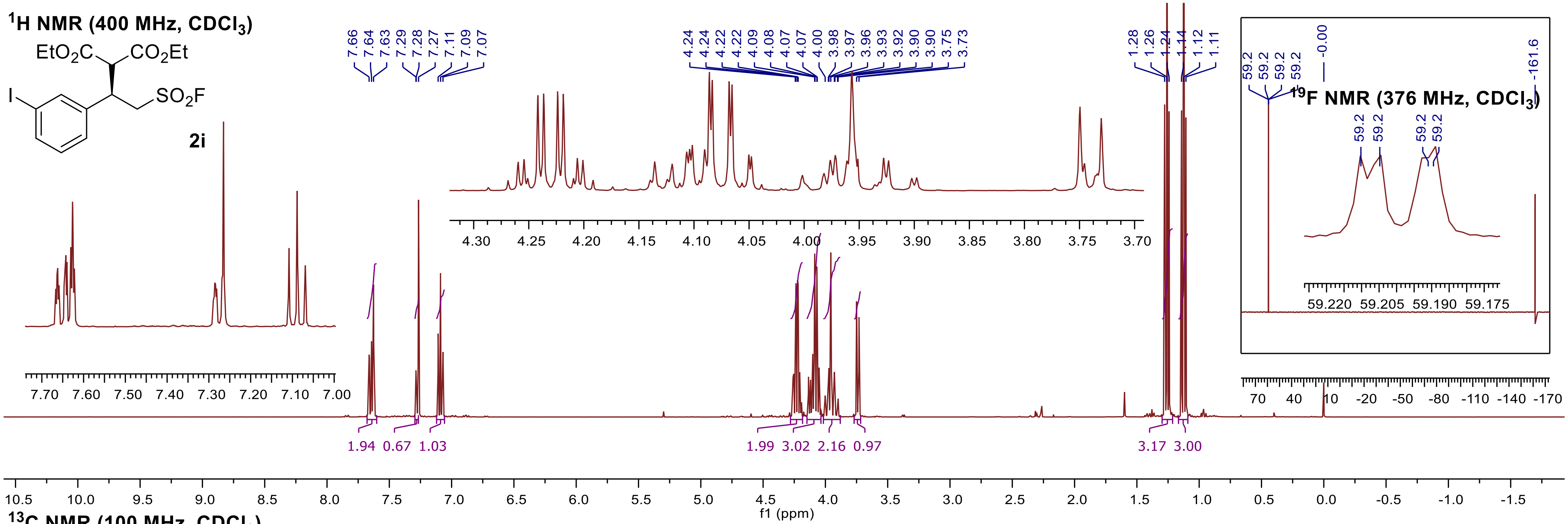
1H NMR (400 MHz, CDCl3)
EtO2C CO2Et
I SO2F
2i
7.66 7.64 7.63 7.29 7.28 7.27 7.11 7.09 7.07
4.24 4.24 4.22 4.22 4.09 4.08 4.07 4.07 4.00 3.98 3.97 3.96 3.93 3.92 3.90 3.90 3.75 3.73
1.28 1.26 1.24 1.14 1.12 1.11
1.94 0.67 1.03
1.99 3.02 2.16 0.97
3.17 3.00
19F NMR (376 MHz, CDCl3)
59.2 59.2 59.2 59.2 -0.00 -161.6
f1 (ppm)

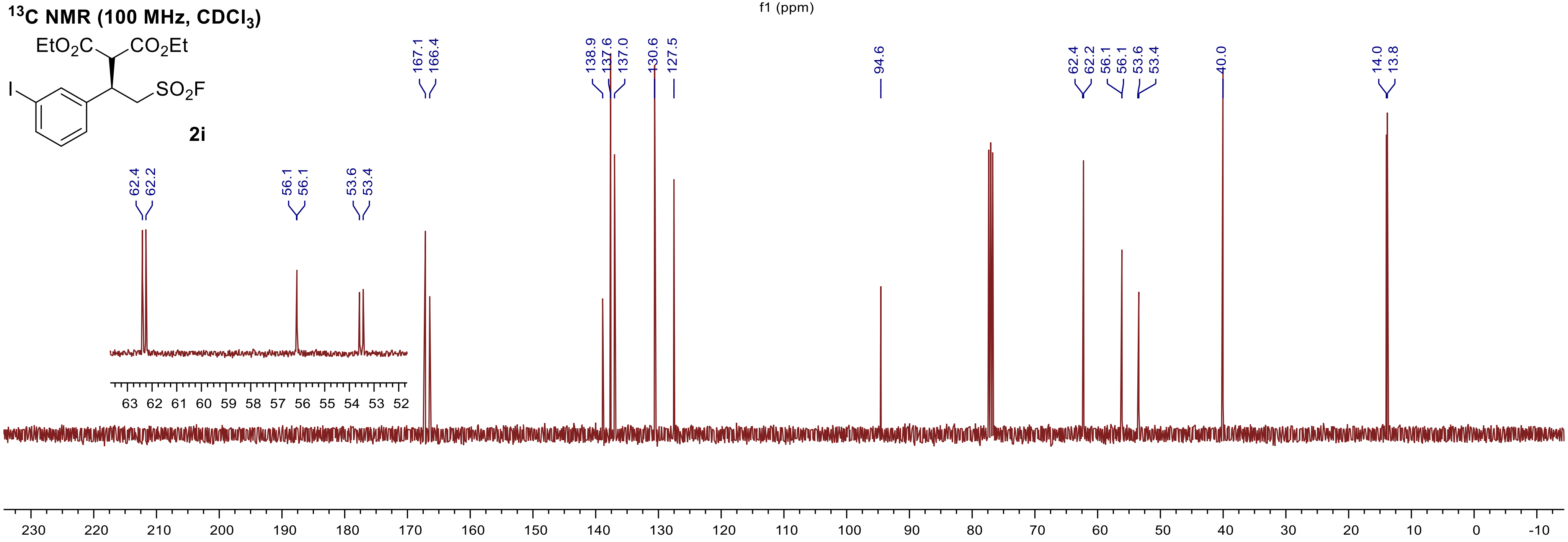
13C NMR (100 MHz, CDCl3)
EtO2C CO2Et
I SO2F
2i
167.1 166.4
138.9 137.6 137.0 130.6 127.5
94.6
62.4 62.2 56.1 56.1 53.6 53.4
40.0
14.0 13.8

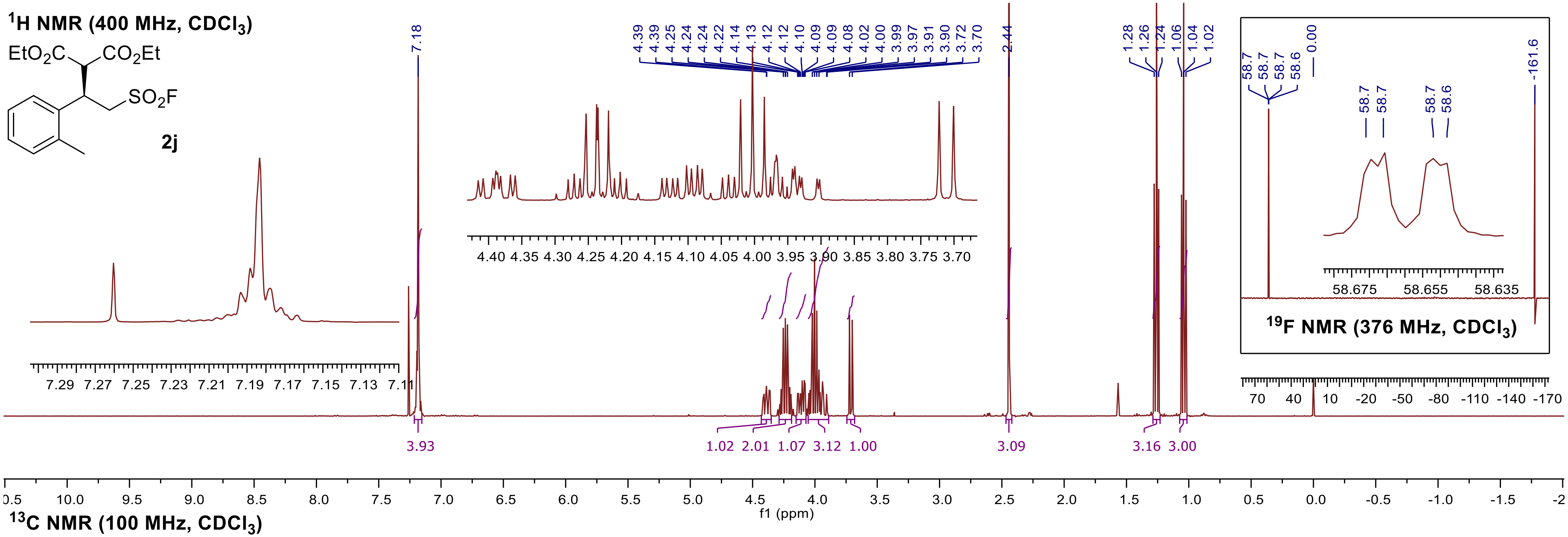

^{1}H NMR (400 MHz, CDCl$_3$)
EtO$_2$C
CO$_2$Et
SO$_2$F
2j
7.18
4.39 4.39 4.25 4.24 4.24 4.22 4.14 4.13 4.12 4.12 4.10 4.09 4.09 4.08 4.02 4.00 3.99 3.97 3.91 3.90 3.72 3.70
2.44
1.28 1.26 1.24 1.06 1.04 1.02
3.93
1.02 2.01 1.07 3.12 1.00
3.09
3.16 3.00
f1 (ppm)
^{19}F NMR (376 MHz, CDCl$_3$)
58.7 58.7 58.7 58.6
0.00
-161.6

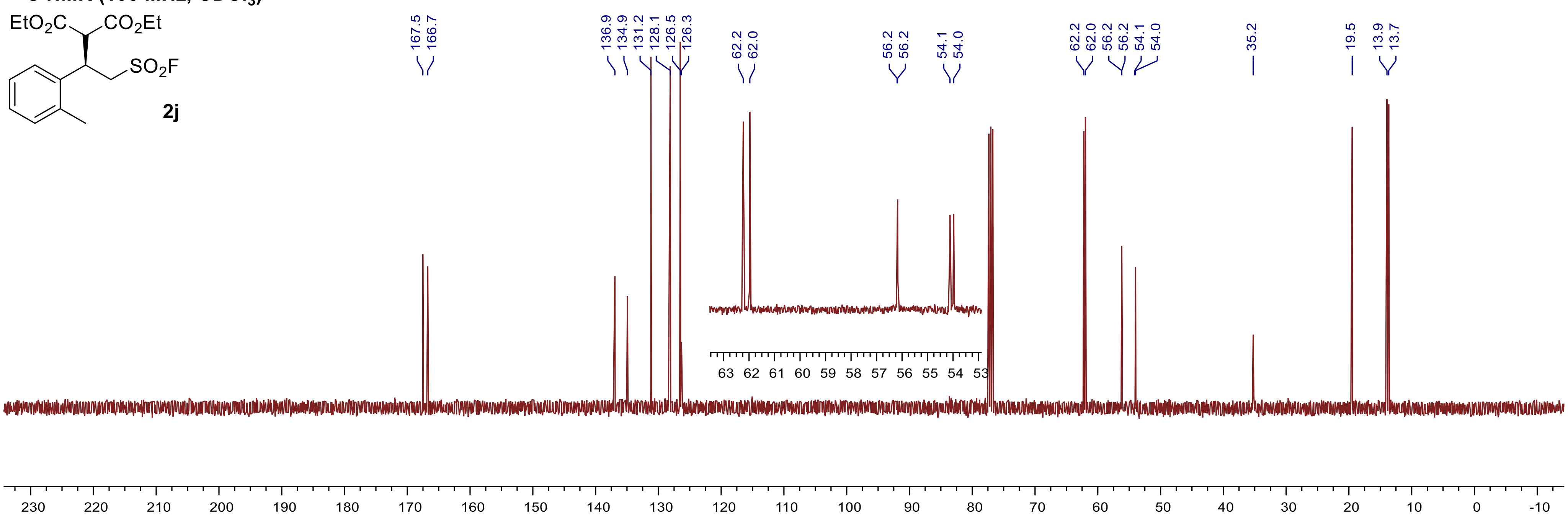

^{13}C NMR (100 MHz, CDCl$_3$)
EtO$_2$C
CO$_2$Et
SO$_2$F
2j
167.5 166.7
136.9 134.9 131.2 128.1 126.5 126.3
62.2 62.0
56.2 56.2
54.1 54.0
62.2 62.0 56.2 56.2 54.1 54.0
35.2
19.5
13.9 13.7

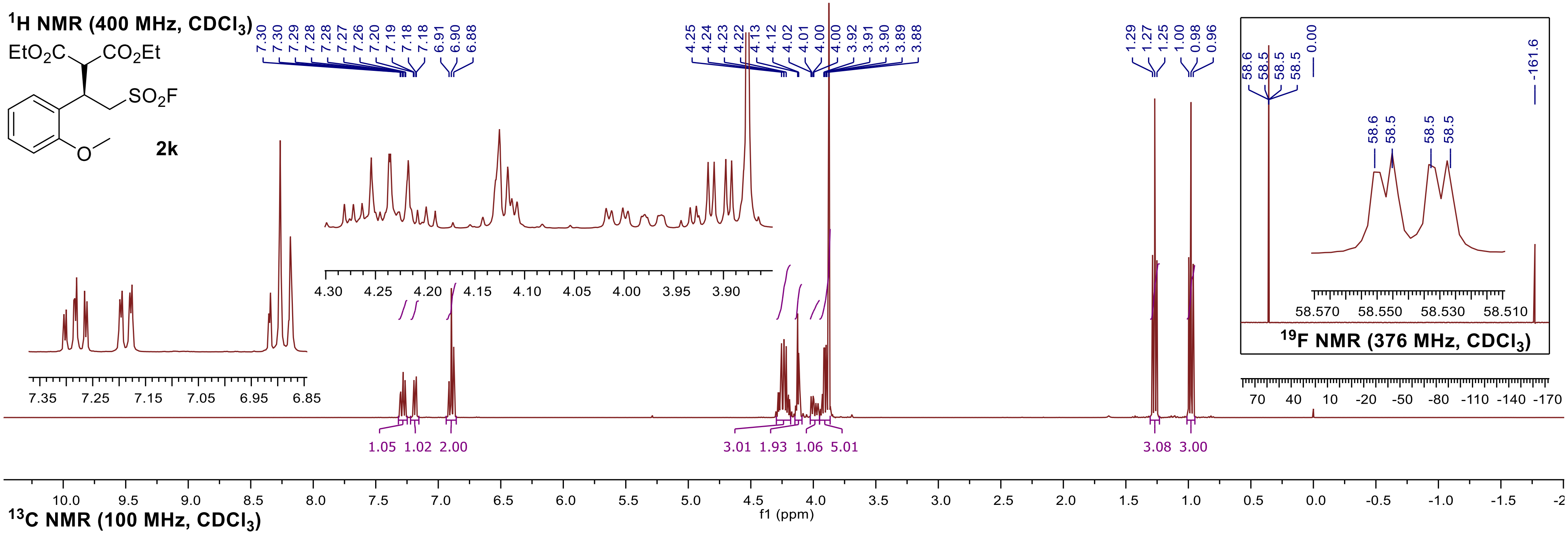
1H NMR (400 MHz, CDCl3)
EtO2C CO2Et
SO2F
O
2k
7.30 7.30 7.29 7.28 7.28 7.27 7.26 7.20 7.19 7.18 7.18 6.91 6.90 6.88
4.25 4.24 4.23 4.22 4.13 4.12 4.02 4.01 4.00 4.00 3.92 3.91 3.90 3.89 3.88
1.29 1.27 1.25 1.00 0.98 0.96
1.05 1.02 2.00
3.01 1.93 1.06 5.01
3.08 3.00
f1 (ppm)
19F NMR (376 MHz, CDCl3)
58.6 58.5 58.5 58.5 0.00 -161.6

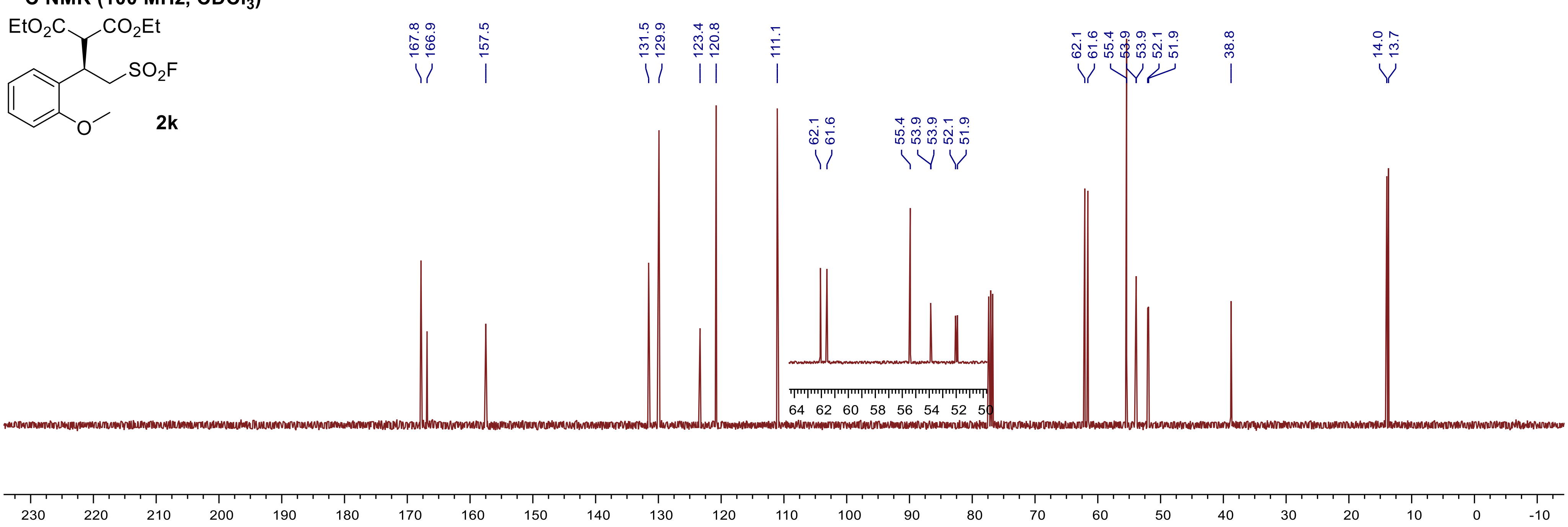
13C NMR (100 MHz, CDCl3)
EtO2C CO2Et
SO2F
O
2k
167.8 166.9 157.5 131.5 129.9 123.4 120.8 111.1
62.1 61.6 55.4 53.9 53.9 52.1 51.9 38.8 14.0 13.7

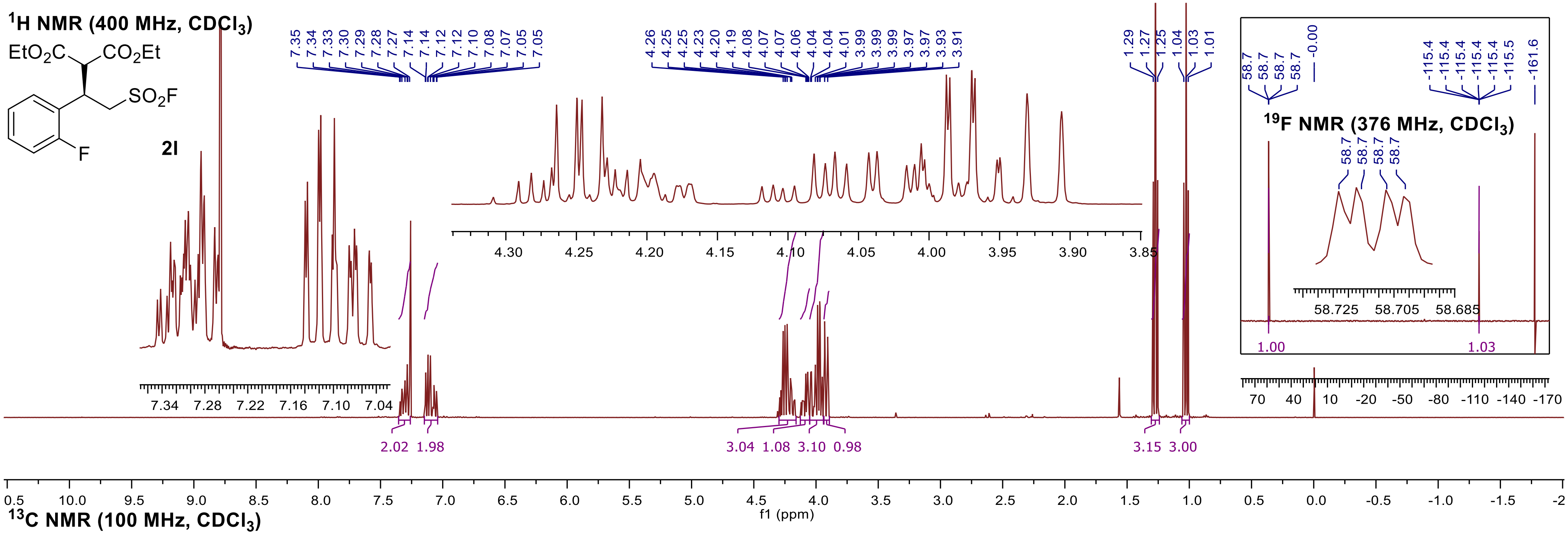

^{1}H NMR (400 MHz, CDCl$_3$)
EtO$_2$C CO$_2$Et
SO$_2$F
F
2l
^{19}F NMR (376 MHz, CDCl$_3$)
f1 (ppm)

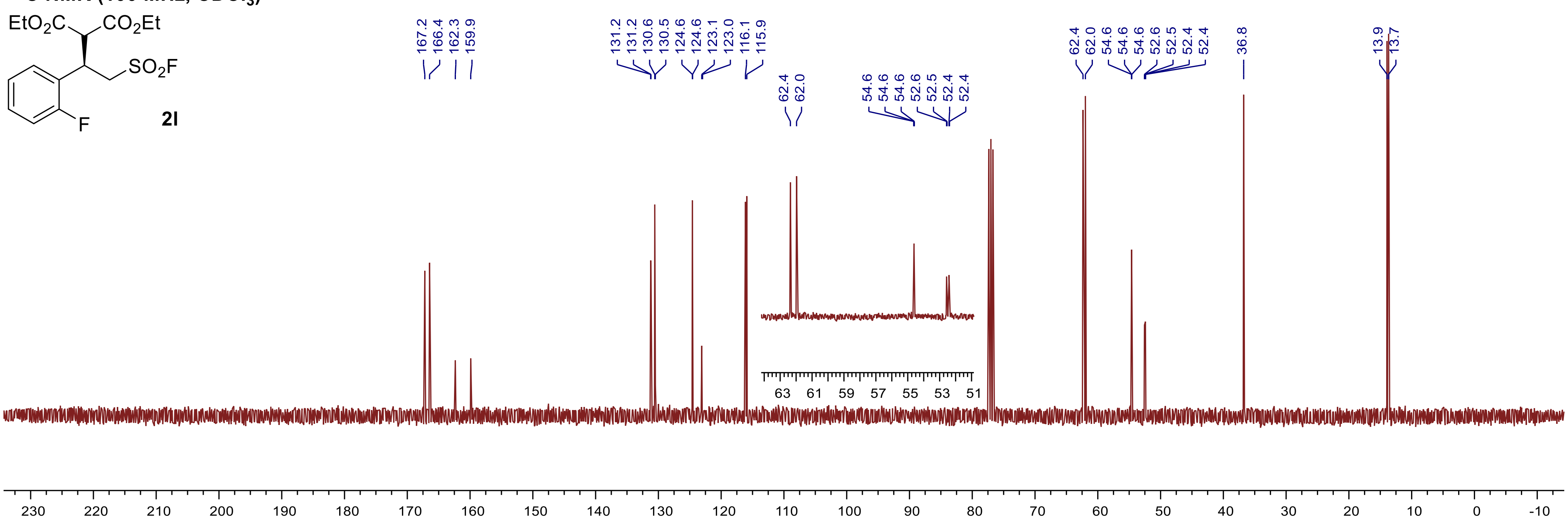

^{13}C NMR (100 MHz, CDCl$_3$)
EtO$_2$C CO$_2$Et
SO$_2$F
F
2l

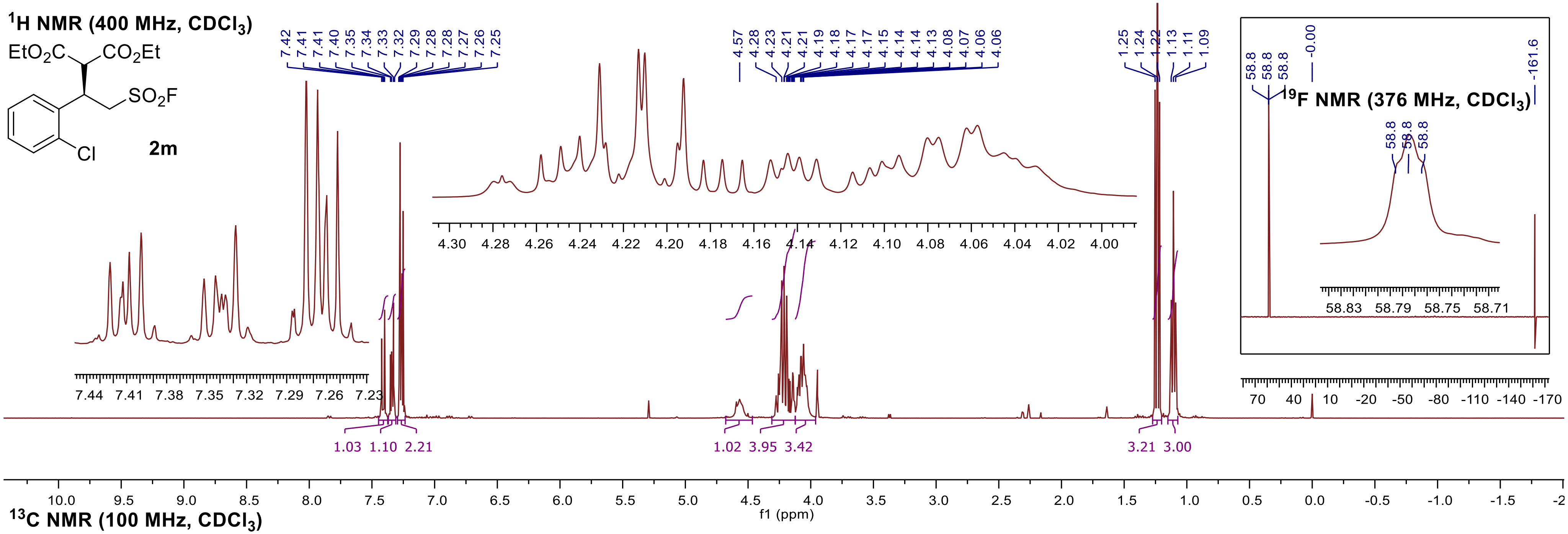

^{1}H NMR (400 MHz, CDCl3)
EtO2C CO2Et
SO2F
Cl
2m
7.42 7.41 7.41 7.40 7.35 7.34 7.33 7.32 7.29 7.28 7.28 7.27 7.26 7.25
4.57 4.28 4.23 4.21 4.21 4.19 4.18 4.17 4.17 4.15 4.14 4.14 4.13 4.08 4.07 4.06 4.06
1.25 1.24 1.22 1.13 1.11 1.09
1.03 1.10 2.21
1.02 3.95 3.42
3.21 3.00
f1 (ppm)
58.8 58.8 58.8 -0.00 -161.6
^{19}F NMR (376 MHz, CDCl3)

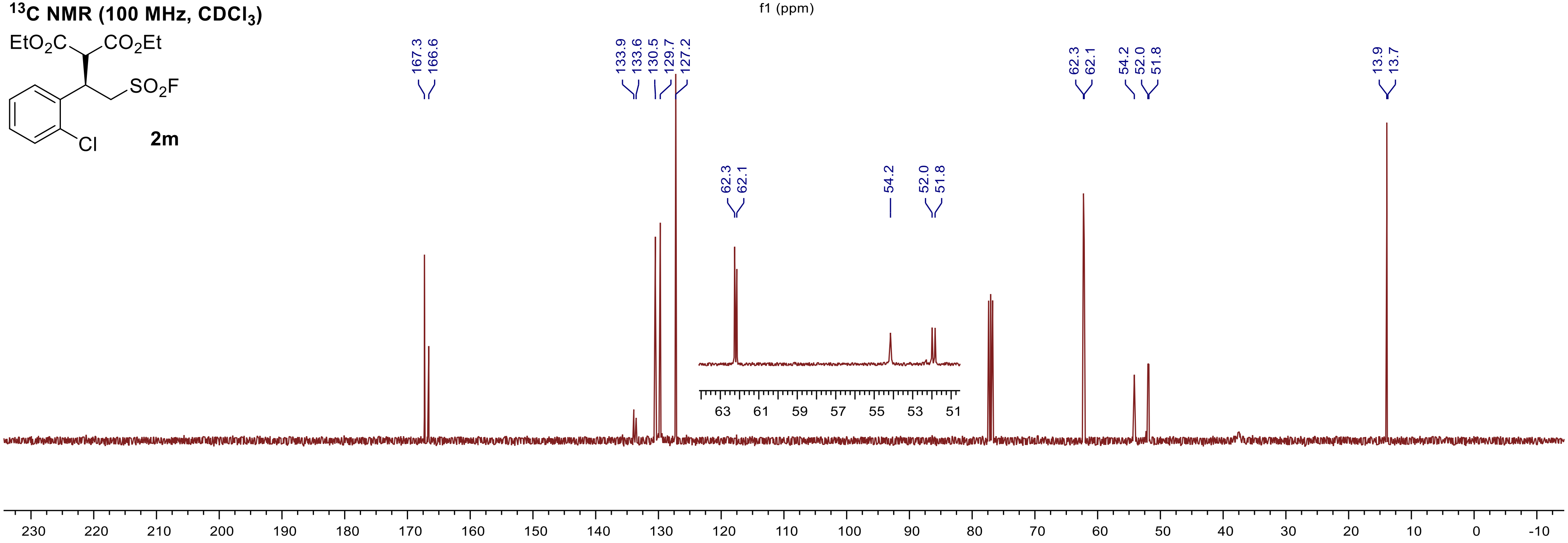

^{13}C NMR (100 MHz, CDCl3)
EtO2C CO2Et
SO2F
Cl
2m
167.3 166.6
133.9 133.6 130.5 129.7 127.2
62.3 62.1
54.2 52.0 51.8
13.9 13.7

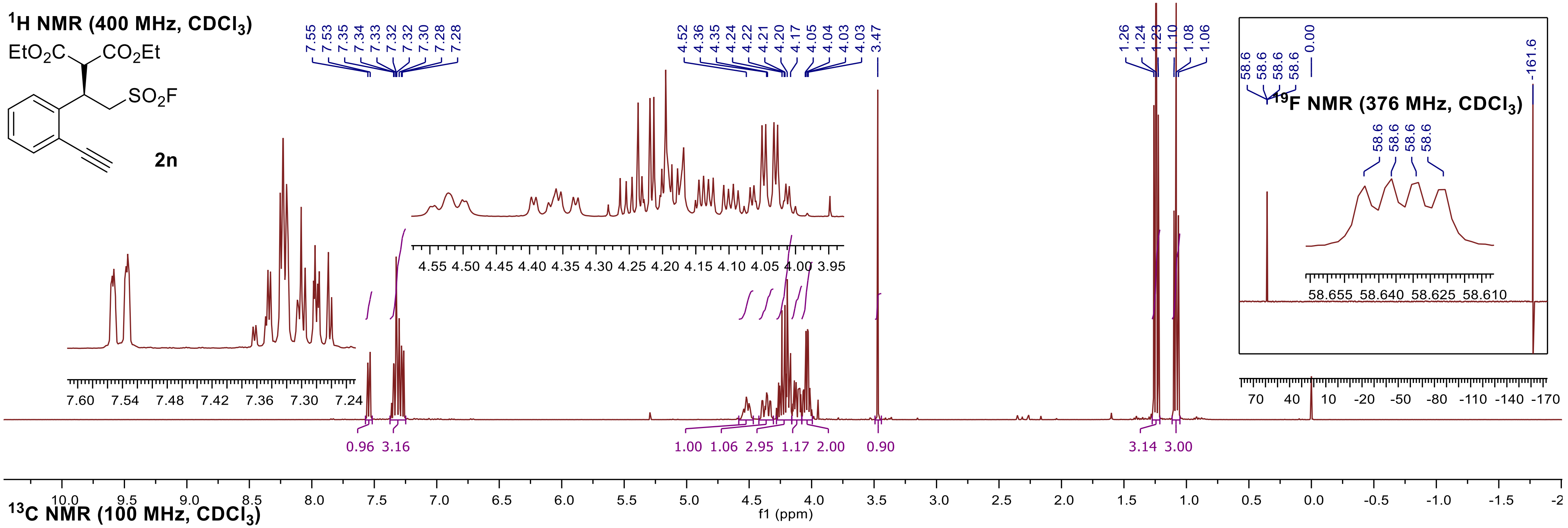

^{1}H NMR (400 MHz, CDCl$_3$)
2n
^{19}F NMR (376 MHz, CDCl$_3$)
f1 (ppm)

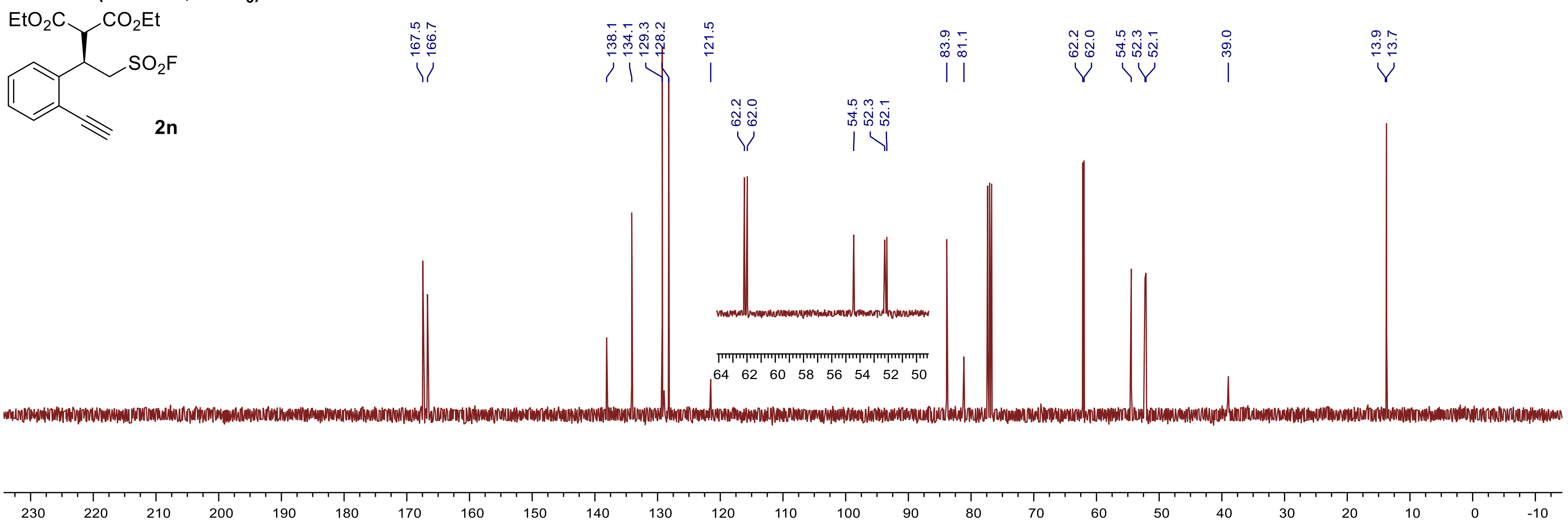

^{13}C NMR (100 MHz, CDCl$_3$)
2n

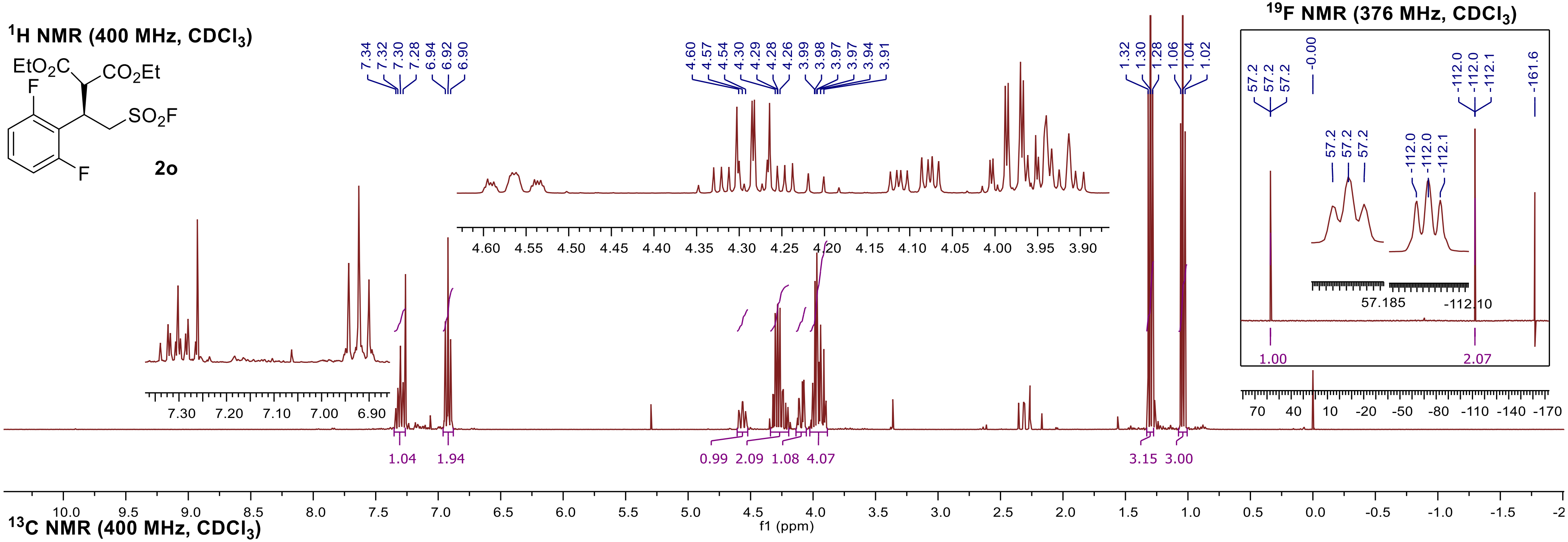
^{1}H NMR (400 MHz, CDCl$_3$)
^{19}F NMR (376 MHz, CDCl$_3$)
2o
f1 (ppm)

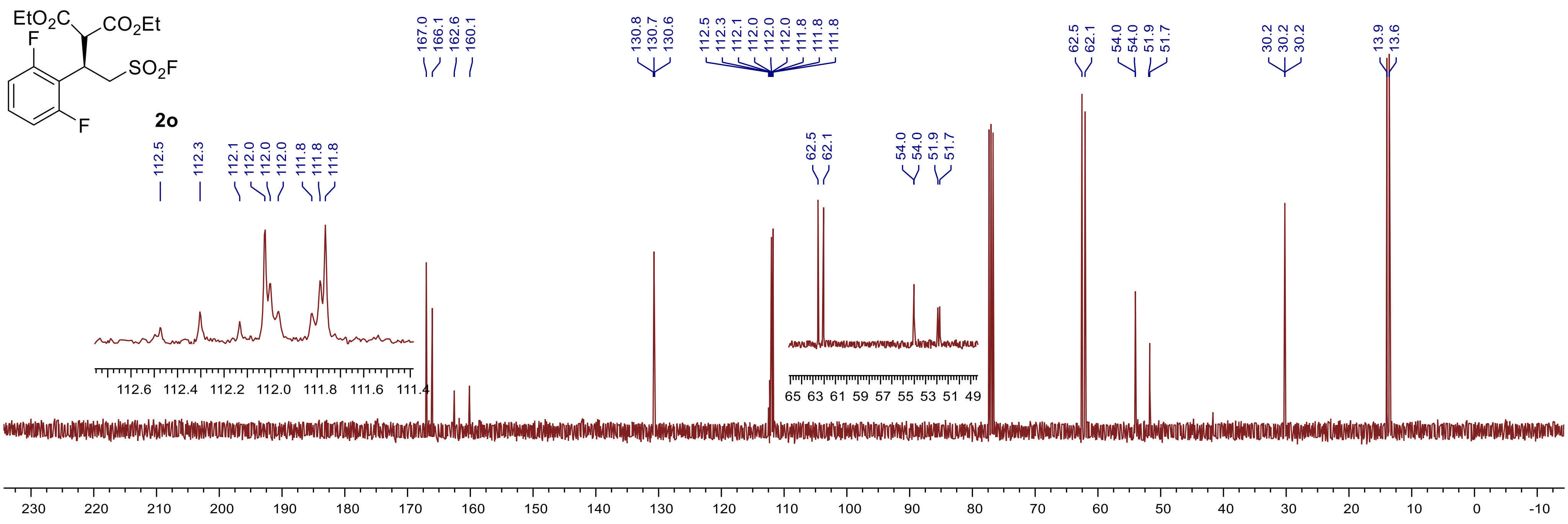
^{13}C NMR (400 MHz, CDCl$_3$)
2o

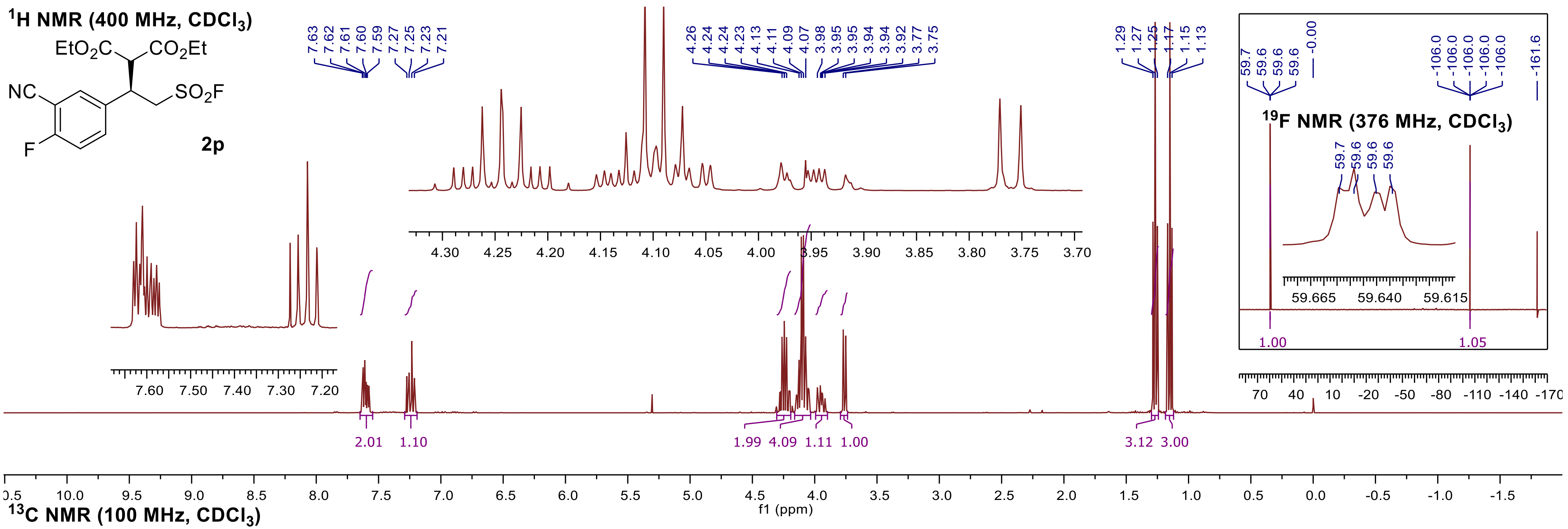

1H NMR (400 MHz, CDCl3)
EtO2C CO2Et
NC
SO2F
F
2p
19F NMR (376 MHz, CDCl3)
f1 (ppm)

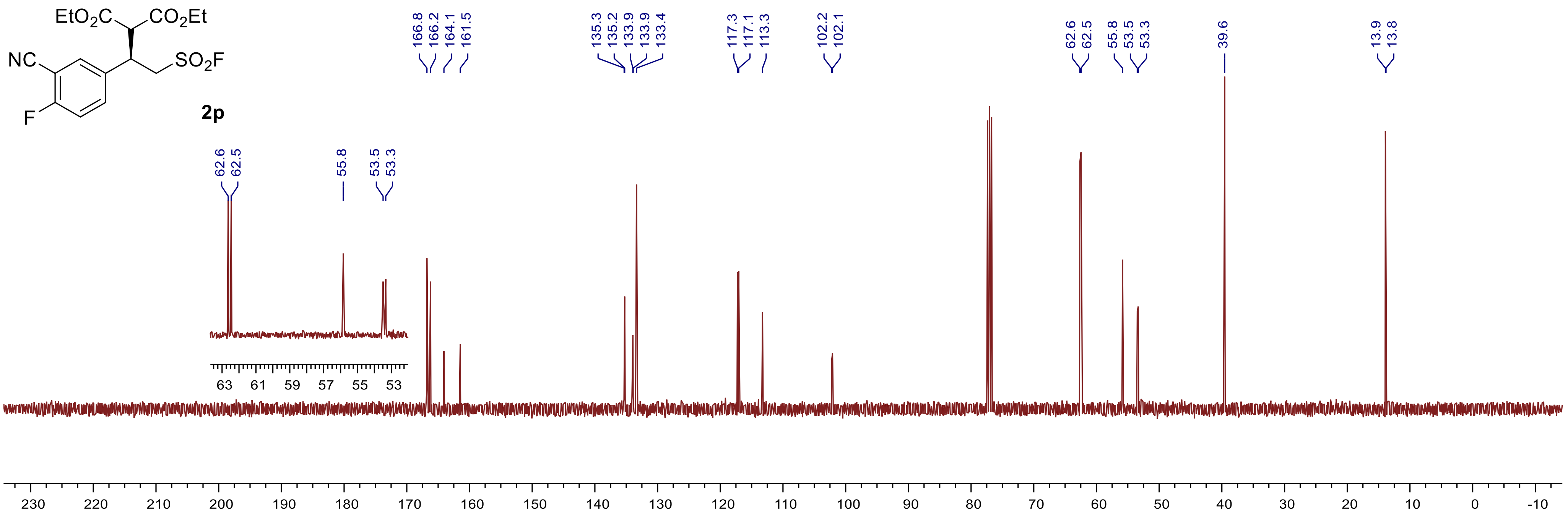

13C NMR (100 MHz, CDCl3)
EtO2C CO2Et
NC
SO2F
F
2p

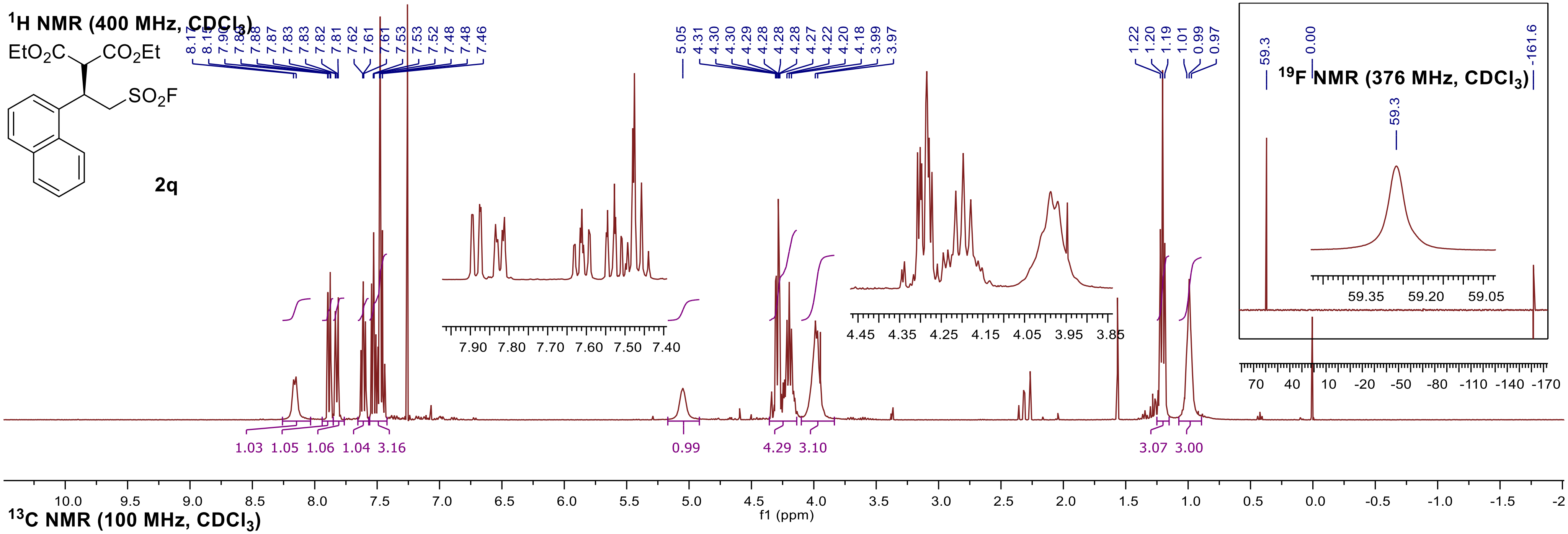
1H NMR (400 MHz, CDCl3)
EtO2C
CO2Et
SO2F
2q
5.05
4.31
4.30
4.30
4.29
4.28
4.28
4.28
4.27
4.22
4.20
4.18
3.99
3.97
1.22
1.20
1.19
1.01
0.99
0.97
1.03 1.05 1.06 1.04 3.16
0.99
4.29 3.10
3.07 3.00
19F NMR (376 MHz, CDCl3)
59.3
0.00
-161.6
f1 (ppm)

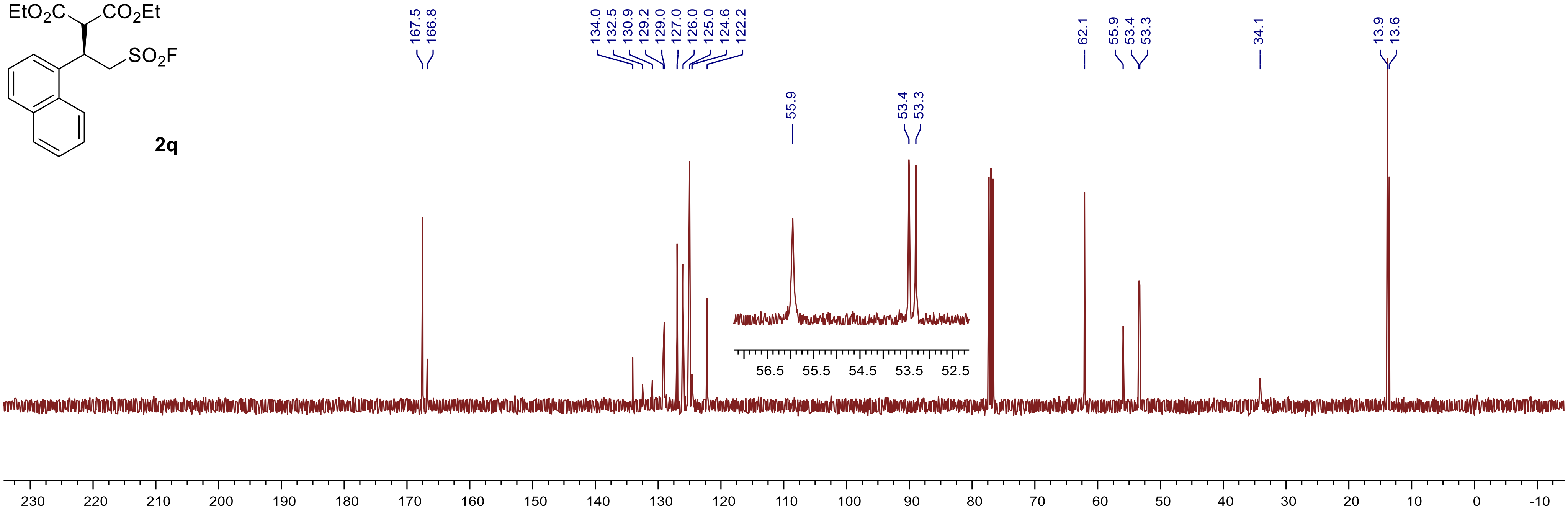
13C NMR (100 MHz, CDCl3)
EtO2C
CO2Et
SO2F
2q
167.5
166.8
134.0
132.5
130.9
129.2
129.0
127.0
126.0
125.0
124.6
122.2
62.1
55.9
53.4
53.3
34.1
13.9
13.6

^{1}H NMR (400 MHz, $CDCl_3$)

^{19}F NMR (376 MHz, $CDCl_3$)

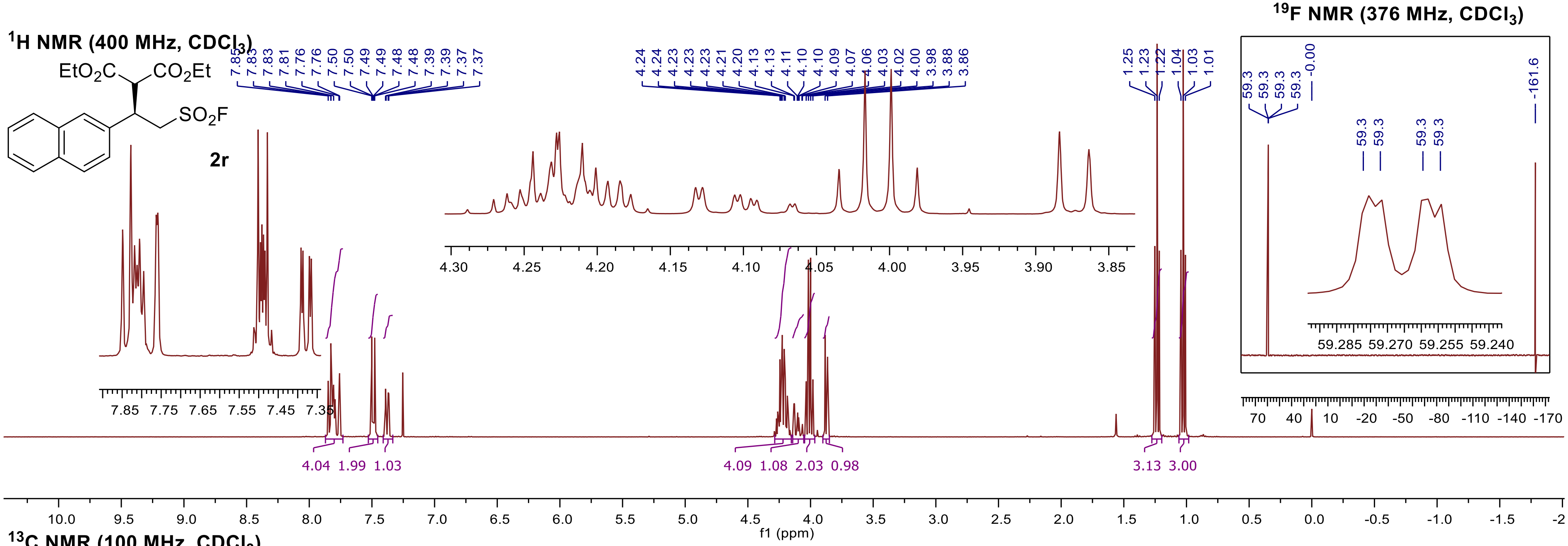

^{13}C NMR (100 MHz, $CDCl_3$)

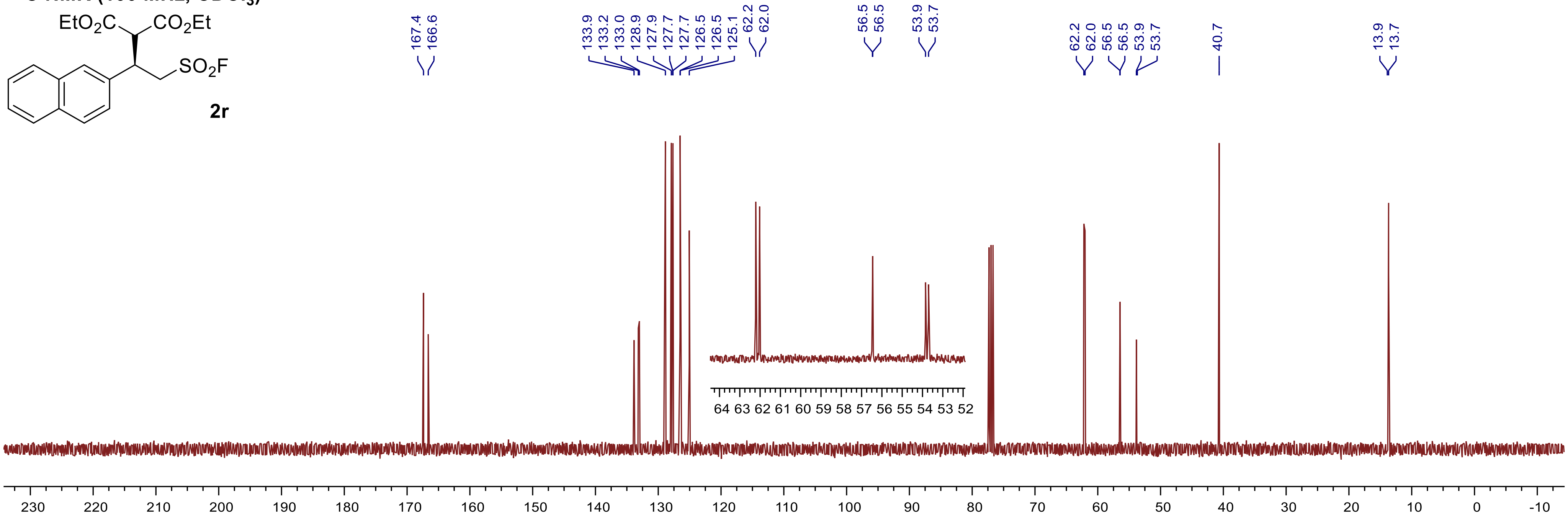

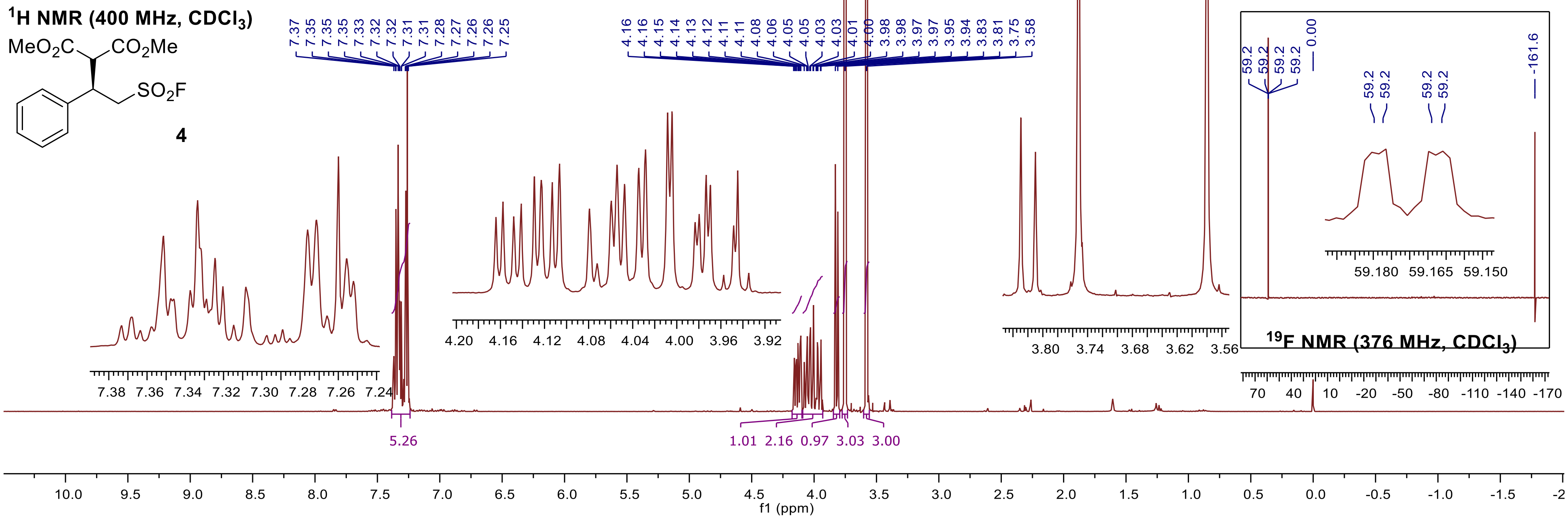

1H NMR (400 MHz, CDCl3)
MeO2C
CO2Me
SO2F
4
19F NMR (376 MHz, CDCl3)
5.26
1.01 2.16 0.97 3.03 3.00
f1 (ppm)

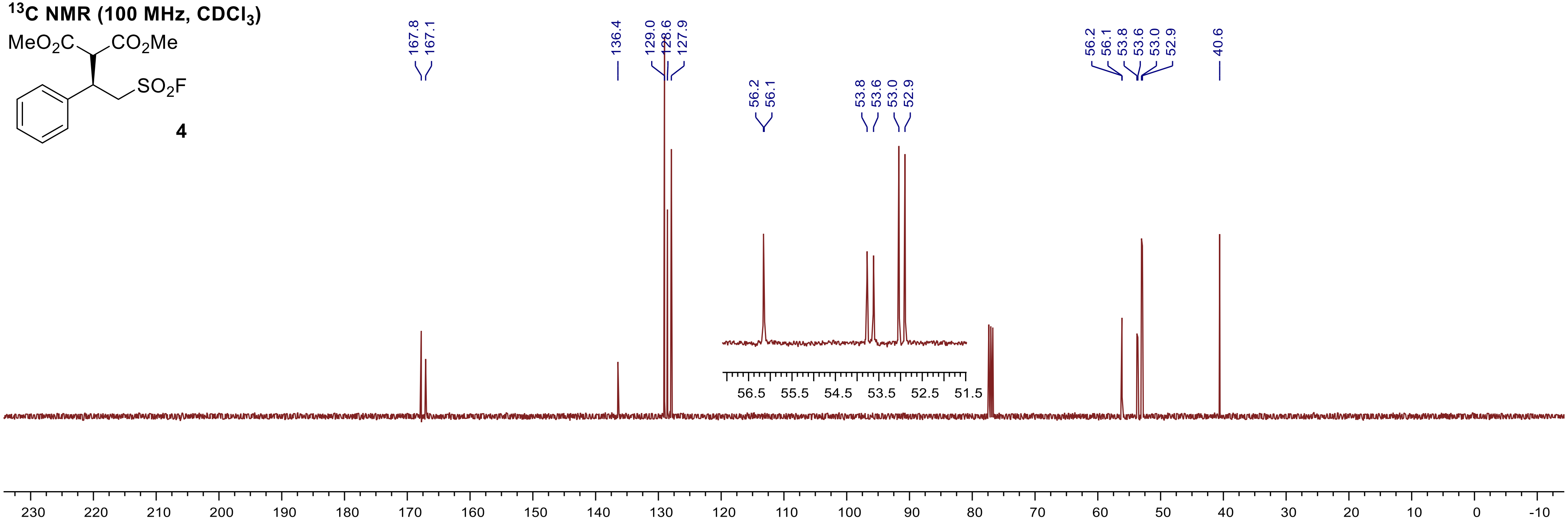

13C NMR (100 MHz, CDCl3)
MeO2C
CO2Me
SO2F
4
167.8
167.1
136.4
129.0
128.6
127.9
56.2
56.1
53.8
53.6
53.0
52.9
40.6

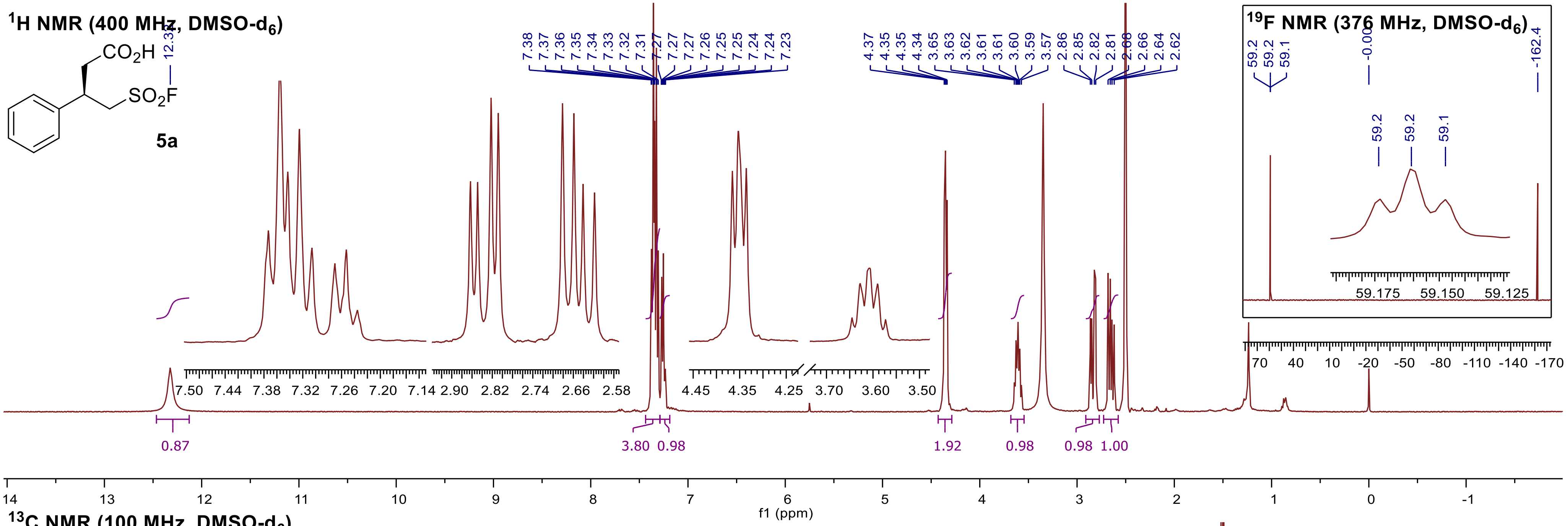

1H NMR (400 MHz, DMSO-d6)
CO2H
SO2F
5a
19F NMR (376 MHz, DMSO-d6)
f1 (ppm)

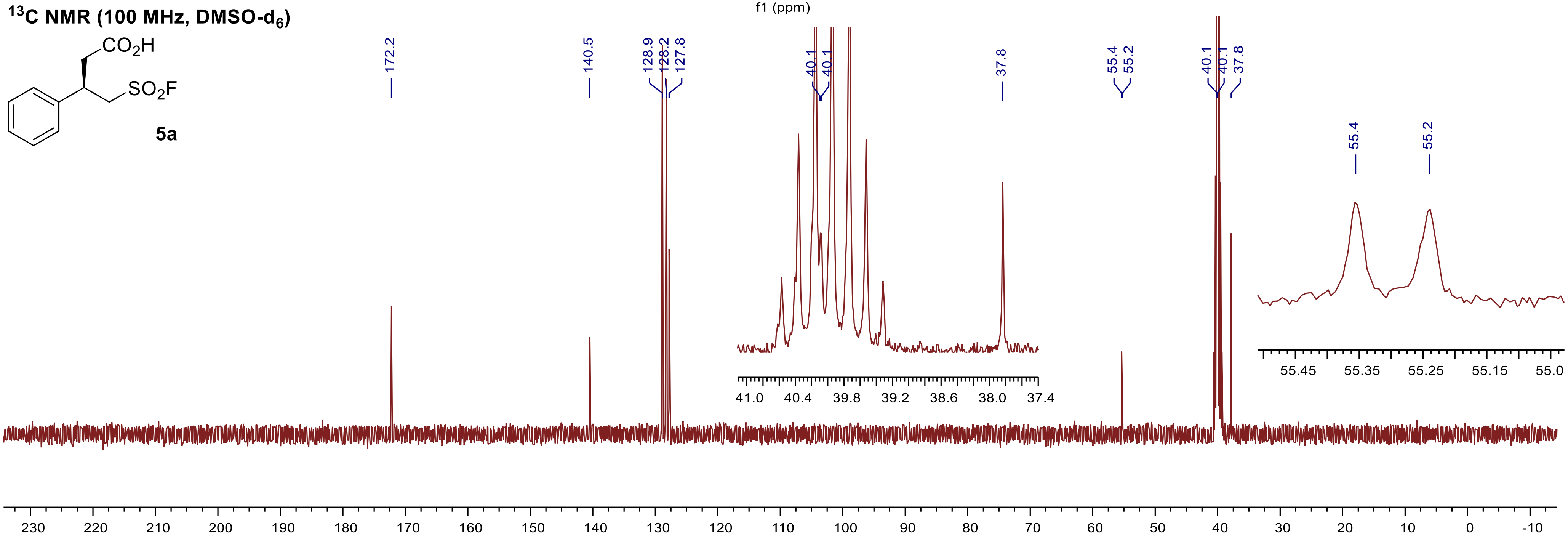

13C NMR (100 MHz, DMSO-d6)
CO2H
SO2F
5a

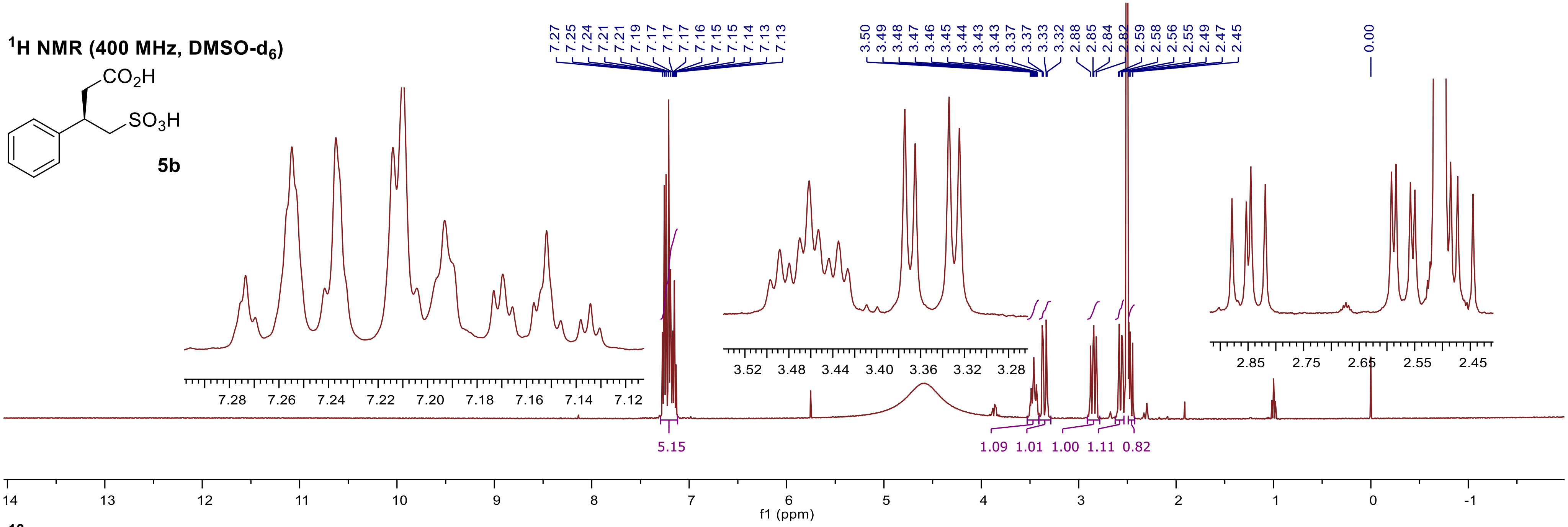
1H NMR (400 MHz, DMSO-d6)
CO2H
SO3H
5b
7.27 7.25 7.24 7.21 7.21 7.19 7.17 7.17 7.17 7.16 7.15 7.15 7.14 7.13 7.13
3.50 3.49 3.48 3.47 3.46 3.45 3.44 3.43 3.43 3.37 3.37 3.33 3.32 2.88 2.85 2.84 2.82 2.59 2.58 2.56 2.55 2.49 2.47 2.45
0.00
5.15
1.09 1.01 1.00 1.11 0.82
f1 (ppm)

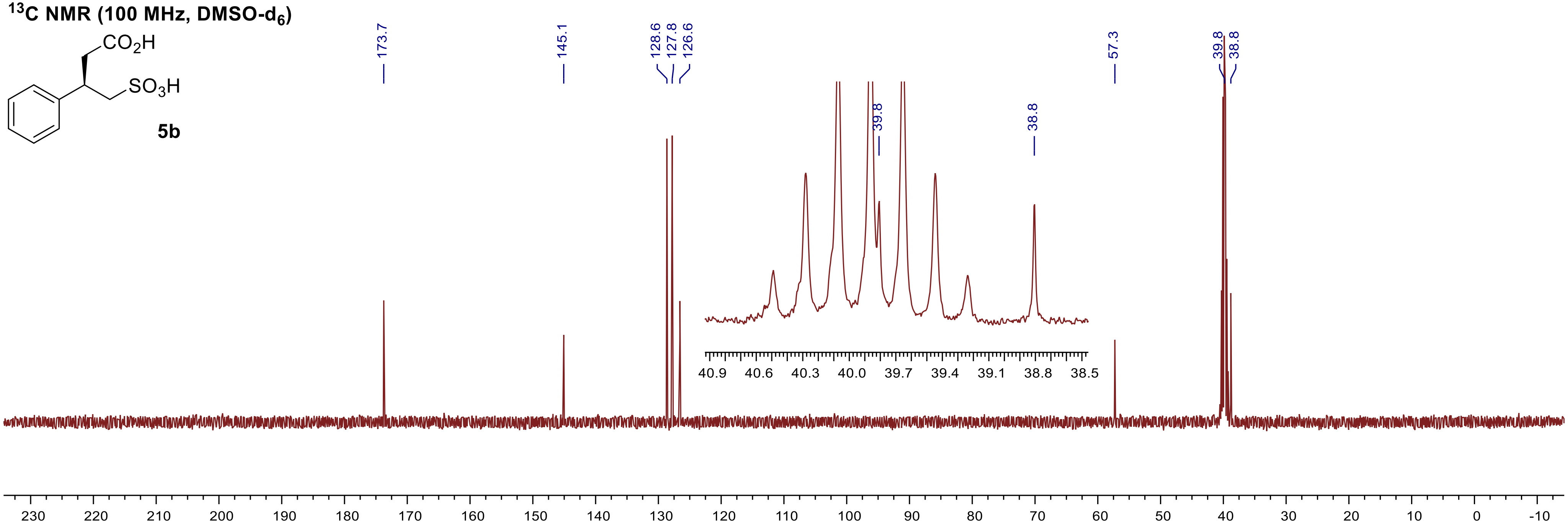
13C NMR (100 MHz, DMSO-d6)
CO2H
SO3H
5b
173.7
145.1
128.6 127.8 126.6
57.3
39.8 38.8

^{1}H NMR (400 MHz, DMSO-d$_6$)

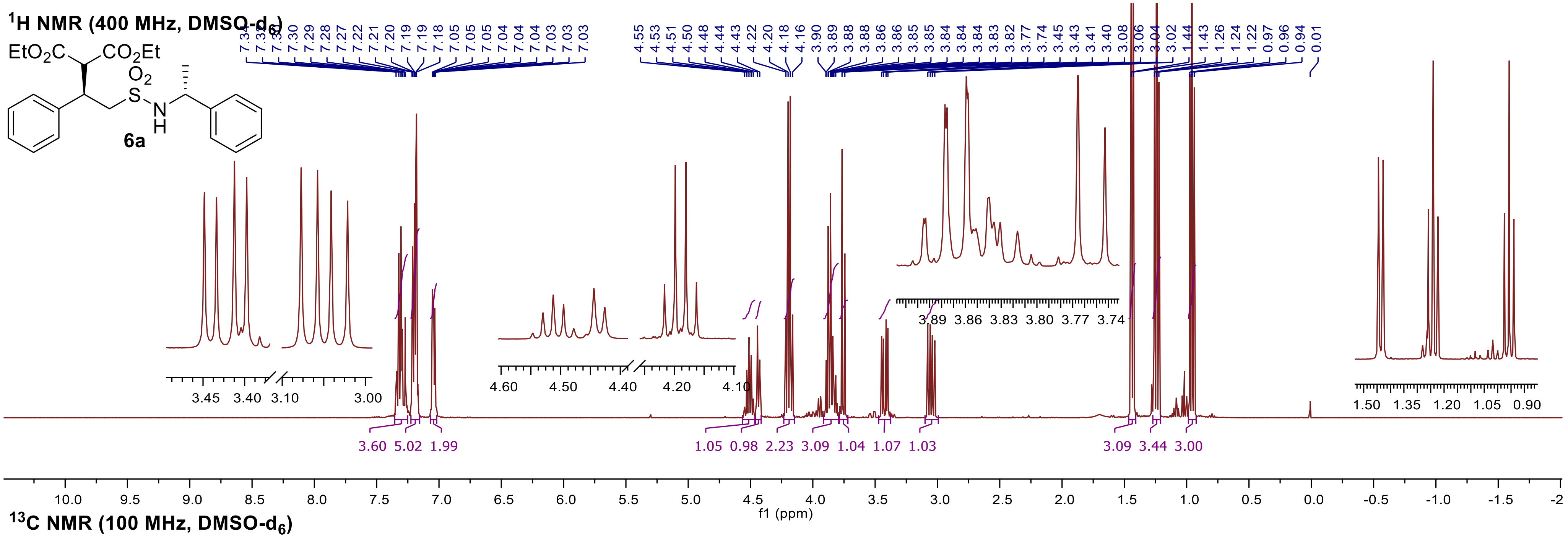

^{13}C NMR (100 MHz, DMSO-d$_6$)

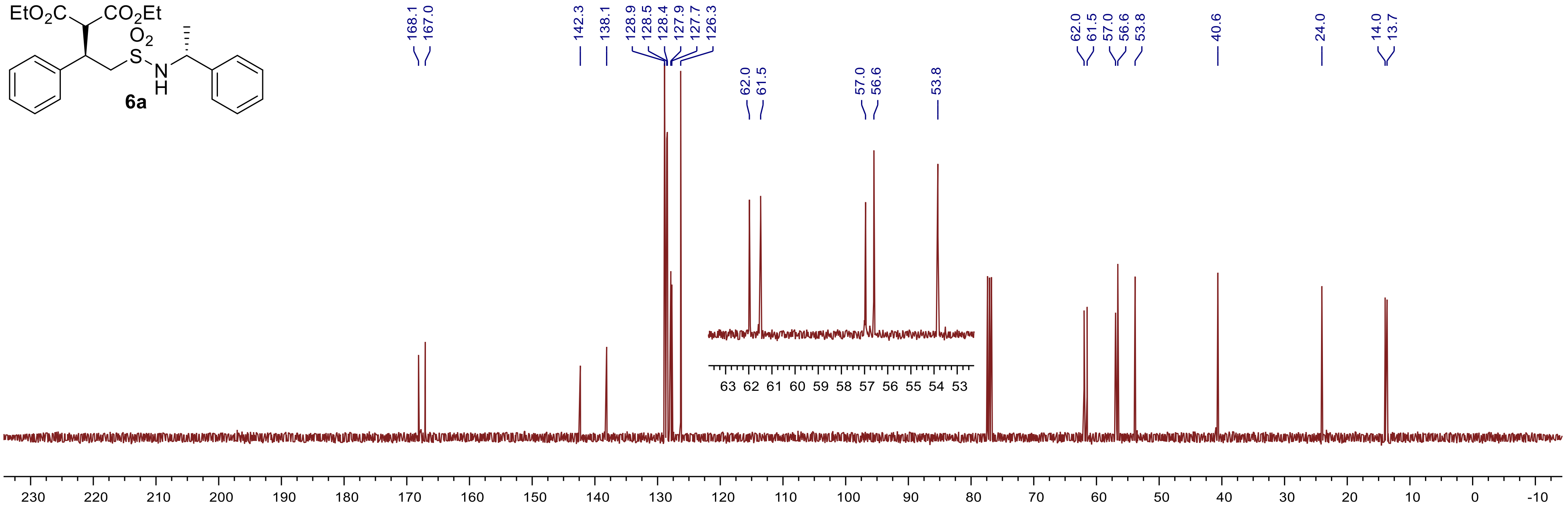

^{1}H NMR (400 MHz, DMSO-d$_6$)

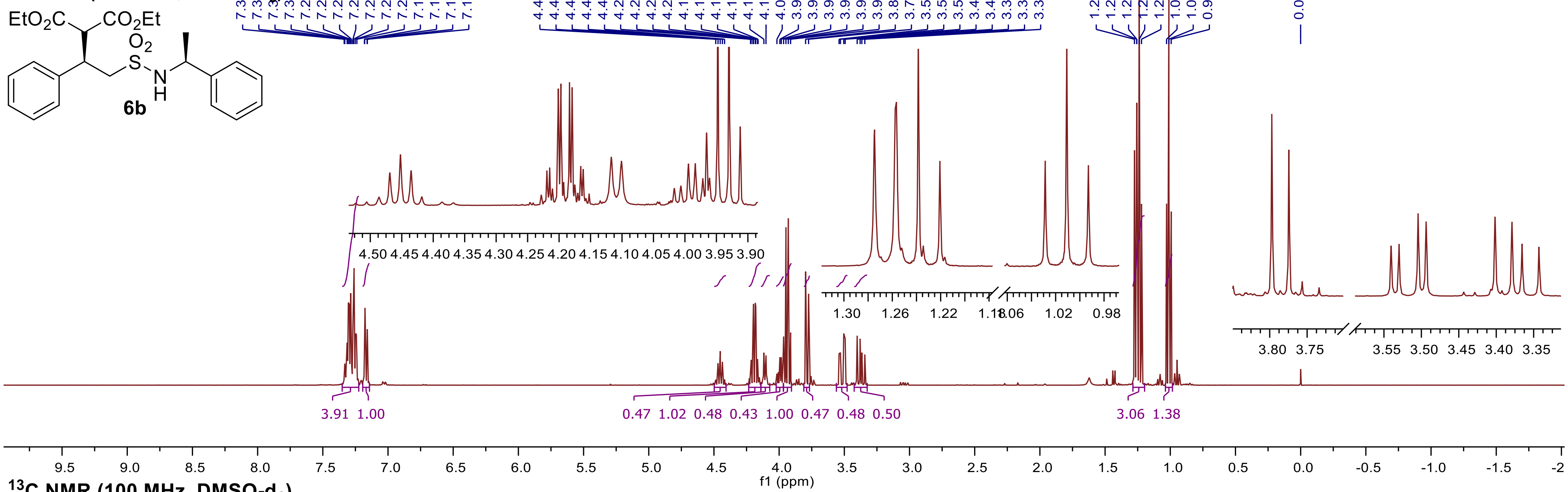

^{13}C NMR (100 MHz, DMSO-d$_6$)

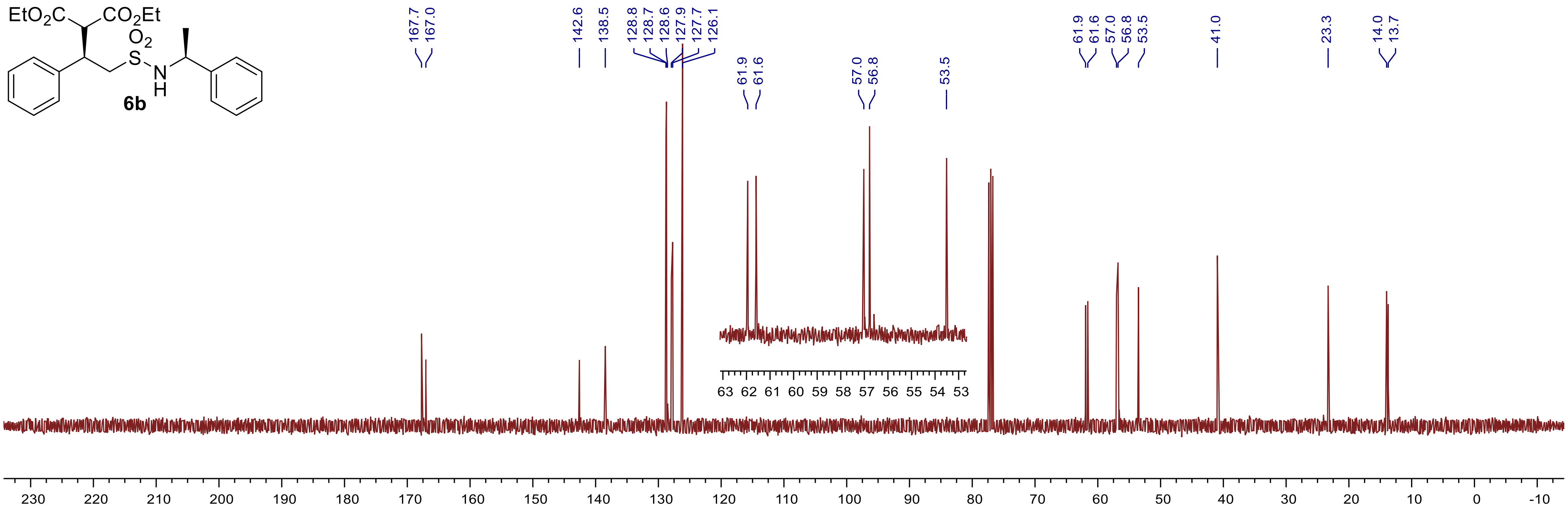

^{1}H NMR (400 MHz, $CDCl_3$)

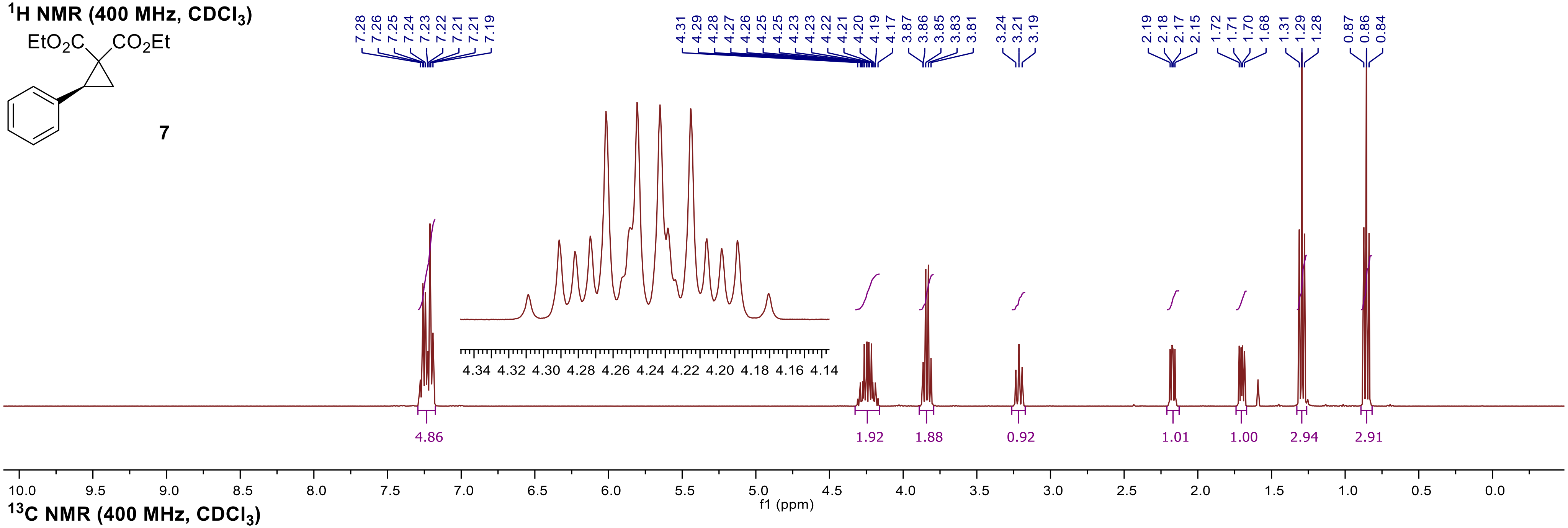

^{13}C NMR (400 MHz, $CDCl_3$)

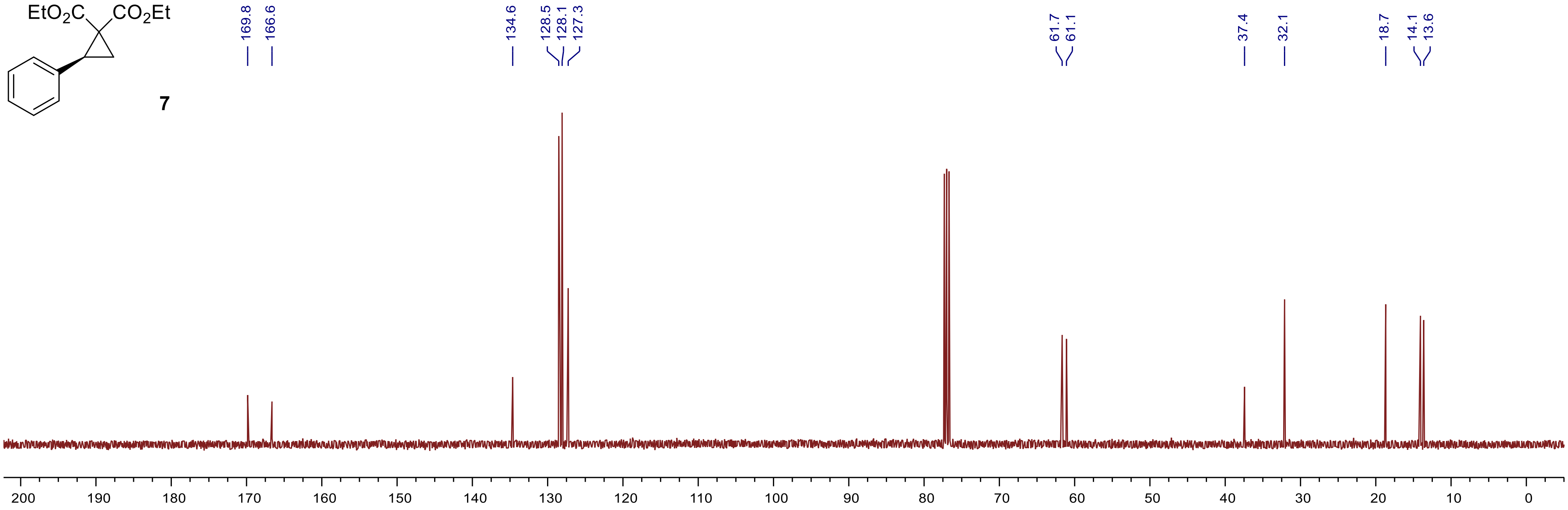

Supplement: Supplementary file 1 — ol3c02302_si_001.pdf [file ol3c02302_si_001.pdf]
